# Total Synthesis of Hemerocallisamine I Paved by Gram-Scale Synthesis of (2*S*,4*S*)-4-Hydroxyglutamic Acid Lactone

**DOI:** 10.3390/molecules28052177

**Published:** 2023-02-26

**Authors:** Lucia Pinčeková, Eva Jančiová, Dušan Berkeš, Róbert Gyepes, Andrej Kolarovič, Oľga Caletková

**Affiliations:** 1 Institute of Organic Chemistry, Catalysis and Petrochemistry, Slovak University of Technology, Radlinského 9, 812 37 Bratislava, Slovakia; 2 Department of Inorganic Chemistry, Faculty of Science, Charles University, Hlavova 2030, 128 40 Prague, Czech Republic; 3 Department of Chemistry, Faculty of Education, Trnava University, Priemyselná 4, 918 43 Trnava, Slovakia

**Keywords:** pyrrole, alkaloid, Maillard reaction, crystallization-induced diastereomer transformation, glutamine, hydroxy amino acid, diketopiperazine

## Abstract

Total synthesis of the 2-formylpyrrole alkaloid hemerocallisamine I is presented, both in racemic and enantiopure form. Our synthetic strategy involves (2*S*,4*S*)-4-hydroxyglutamic acid lactone as the key intermediate. Starting from an achiral substrate, the target stereogenic centers were introduced by means of crystallization-induced diastereomer transformation (CIDT) in a highly stereoselective fashion. A Maillard-type condensation was crucial to constructing the desired pyrrolic scaffold.

## 1. Introduction

Daylilies (genus *Hemerocallis*) are beautiful flowering plants, now counting 16 species and over 98,000 cultivars, widely domesticated in much of the Northern Hemisphere [1,2]. Mentioned in poems compiled by Confucius more than 2500 years ago, in addition to ornamental function, they have a long history of culinary [3] and medicinal use in Eastern Asia. The flowers, buds, leaves, and roots of daylilies are edible and have been reported to be utilized, e.g., in the treatment of sleep disorders, depression, inflammation, jaundice, schistosomiasis, and chronic rheumatism [4]. In an attempt to elucidate the structures of the active ingredients, the chemical constitution of daylilies has been investigated in a number of studies. As of 31 December 2020, a total of 266 secondary metabolites have been identified in *Hemerocallis* plants, primarily focusing on species *H. citrina*, *H. fulva*, and *H. minor* [4,5].

**Figure 1:**
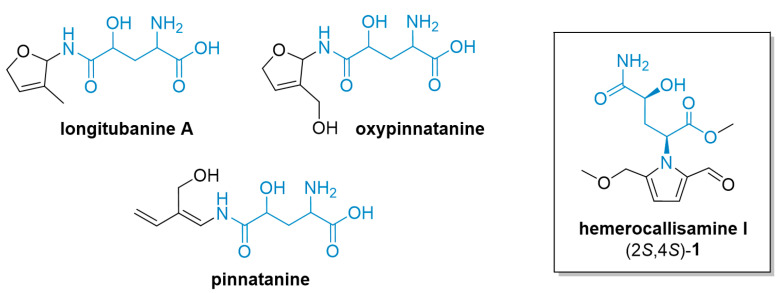
Structures of 4-hydroxyglutamine derivatives isolated from daylily *Hemerocallis fulva*.

In a search to identify sedative amino acid derivatives from *H. fulva* flower buds, in 2014, Matsuda et al. isolated hemerocallisamine I (**1**) as a novel 4-hydroxyglutamine metabolite [6] (Figure 1). The initially assigned (2*R*,4*R*)-**1** configuration, on the basis of the Flack parameter, corresponded to the unnatural D-glutamine and was puzzling. Three years later, the first total synthesis of hemerocallisamine I (**1**) was reported by Brimble et al. and resulted in a revision of the previously proposed absolute configuration from (2*R*,4*R*)-**1** to (2*S*,4*S*)-**1 [7]**. Until now, no other total synthesis of hemerocallisamine I (**1**) has been communicated.

The structure of hemerocallisamine I (**1**) features a 4-hydroxyglutamine moiety anchored in a 2-formylpyrrole ring (Figure 1). Both of these subunits are abundant in nature and of synthetic interest [8,9,10]. In addition to hemerocallisamine I (**1**), several other 4-hydroxyglutamine derivatives, e.g., longitubanine A, oxypinnatanine, and pinnatanine (Figure 1), as well as their *N*-glycosides, have been isolated directly from *H. fulva* species [11,12,13]. Interestingly, the findings of Ogawa and coworkers indicate that oxypinnatanine might be the compound responsible for the sleep-promoting effect of *H. fulva* [14,15].

**Scheme 1:**
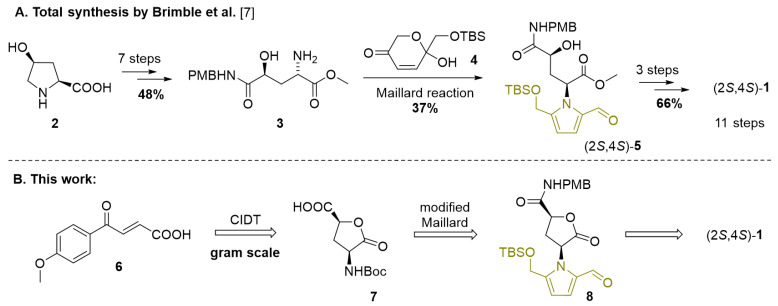
(**A**) The first total synthesis of hemerocallisamine I (**1**), featuring Maillard-type condensation as the key step. (**B**) Our alternative synthetic approach to hemerocallisamine I (**1**). PMB = *p*-methoxybenzyl, TBS = *tert*-butyldimethylsilyl, Boc = *tert*-butyloxycarbonyl.

With the aim of smoothly constructing the 4-hydroxyglutamine framework, the published synthesis of (2*S*,4*S*)-**1** started with commercially available *cis*-4-hydroxy-L-proline (**2**) to deliver amine **3** in 48% yield over 7 steps [7] (Scheme 1A). The critical step, Maillard-type condensation with dihydropyranone **4**, provided the functionalized pyrrole (2*S*,4*S*)-**5** only in a moderate yield of 37%. After the final deprotections and selective *O*-methylation, an 11-step synthesis of hemerocallisamine I (**1**) was successfully concluded with an 11.7% overall yield.

Herein we describe an alternative synthetic access to hemerocallisamine I (**1**), starting from the achiral substrate **6** (Scheme 1B). Given the importance of the 4-hydroxyglutamic motif in the chemistry of daylilies, we opted for facile stereoselective access to lactone **7** as a key intermediate.

## 2. Results and Discussion

Along with procedures employing *trans-* and *cis*-4-hydroxy-L-proline derivatives [16,17,18], utilized by Brimble et al. (Scheme 1A), synthetic strategies leading to non-racemic 4-hydroxyglutamic acids include enzymatic [19] and classic resolution [20], C4-hydroxylations of L-glutamic substrates [21,22,23], and cycloadditions [24,25]. Since the number of synthetic methods available for the preparation of enantiopure 4-hydroxyglutamates is rather limited [26], we wished to develop a new, scalable, and configurationally flexible protocol based on crystallization-induced diastereomer transformations (CIDT) [27].

### 2.1. Synthesis of (±)-hemerocallisamine I and Optimization of the Maillard Reaction Step

Considering that the synthesis of racemic hemerocallisamine I remains undescribed and that this material might be of value, e.g., for biological activity investigation, our synthetic approach was first evaluated in its achiral variant.

**Scheme 2:**
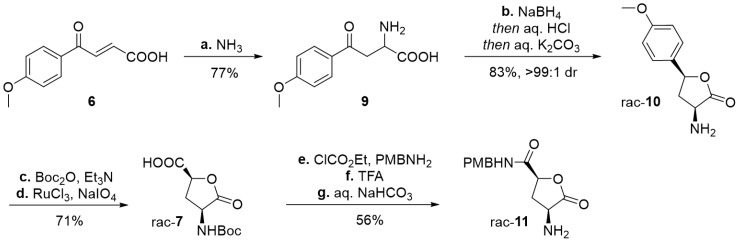
Preparation of lactone rac-**11**. Reagents and conditions: (**a**) 25–27% aq. NH_3_ (*ca* 4.5 equiv), MeOH, rt, 16 h; (**b**) NaBH_4_ (3.5 equiv), MeOH/H_2_O 5:1, rt, 90 min; then conc. HCl/H_2_O 1:2, rt, 4 h; then 10% aq. K_2_CO_3_/H_2_O 5:4; (**c**) Boc_2_O (1.1 equiv), Et_3_N (2.2 equiv), THF, rt, 4 h (94%); (**d**) 6 mol% RuCl_3_, NaIO_4_ (19.4 equiv), H_2_O/MeCN/AcOEt 3:1:1, 10 °C → rt, 3 h (75%); (**e**) ClCO_2_Et (2.0 equiv), Et_3_N (2.5 equiv), dry THF, 15 min, 0 °C; then PMBNH_2_ (2.7 equiv), 0 °C, 2 h (66%); (**f**) TFA (10.6 equiv), CH_2_Cl_2_, 0 °C → rt, 3 h (95%); (**g**) 10% aq. NaHCO_3_, CH_2_Cl_2_, (90%). Boc = *tert*-butyloxycarbonyl, PMB = *p*-methoxybenzyl, TFA = trifluoroacetic acid.

The studies commenced with a routine construction of amino acid **9** by means of aza-Michael addition (Scheme 2). Shortly after mixing an excess of aq. NH_3_ with aroylacrylic substrate **6** in MeOH, adduct **9** started to precipitate out of the reaction mixture and was filtered off in 77% yield. A consequent reduction step delivered a diastereomeric mixture of the corresponding γ-aryl-γ-hydroxy-α-amino acids, in a ratio *ca* 2:1. However, our one-pot CIDT protocol exploited configurational lability of the benzylic γ-hydroxy group in dilute aqueous HCl [28,29] and provided crystalline *cis*-lactone salt rac-**10**·HCl and then free amine rac-**10** in excellent diastereomeric purity (>99:1 dr). After its *N*-Boc protection, the adjacent electron-rich anisole ring was oxidatively cleaved employing a catalytic amount of in situ prepared RuO_4_, with NaIO_4_ as a reoxidant [30,31]. Our modified procedure furnished the 4-hydroxyglutamic acid lactone rac-**7** in 71% yield, over two steps. When approaching the critical Maillard reaction step, we were inspired by the studies of Brimble et al. [7] and chose to introduce the *p*-methoxybenzyl (PMB) protected amide group. The coupling with PMBNH_2_ was mediated by ethyl chloroformate and a subsequent Boc-removal provided amine rac-**11** as a synthetic alternative to amine **3** (Scheme 1A). Pleasingly, each of the steps in the **6**–**11** sequence delivered crystalline compounds and are convenient to purify, isolate, and store.

The Maillard condensations targeting the 2-formylpyrrole framework have often proved challenging [9,10]. The reported Maillard protocol towards hemerocallisamine I harnessed an open glutamine chain **3** (Scheme 1A). We hypothesized that the application of lactone **11** instead might reduce the complexity of the amine building block and provide better yields. Seeing that dihydropyranone **4** was frequently reported as a suitable sugar surrogate in Maillard reactions [7,32,33,34,35,36,37], it was picked as a reaction partner for our optimization studies (Table 1). In the published procedures, amines were usually used in 1.5- to 4-fold excess for the sake of better yields [32,33,35,36]. In our case, considering that amine **11** is not an effortless building block, it was reasonable to screen reaction conditions with equimolar amounts of rac-**11** and **4**. Somewhat confusingly, the currently recorded optimized protocols vary in the suggested reaction conditions from case to case. Pyridine [7], undistilled dioxane [32], THF/H_2_O [33,34,35], and MeCN [36] were used as solvents and Et_3_N [32], AcOH [35], and pyridinium *p*-toluenesulfonate [7,36] as additives, at reaction temperatures ranging from rt to 60 °C. Dihydropyranone **4** was occasionally observed to remain partially unreacted and was recovered in 8–35% yields [33,34]. Taking into account all these data, we performed a broader investigation (selected examples are in Table 1, for details, see the Supplementary Materials), screening diverse solvents and additives (entries 2–12). As found, simple heating of rac-**11** and **4** in dry toluene at 70 °C gave the best outcome, and the target 2-formylpyrrole rac-**8** was isolated in 43% yield (entry 1). Interestingly, 2,5-diketopiperazine rac-**12** was repeatedly detected as a side product in the reaction mixtures, in yields of up to 35% with respect to amine rac-**11 [38]**. These findings provide an important argument when explaining why Maillard condensations so often fail to deliver better yields and should be reflected in future synthetic strategies.

**Scheme 3:**
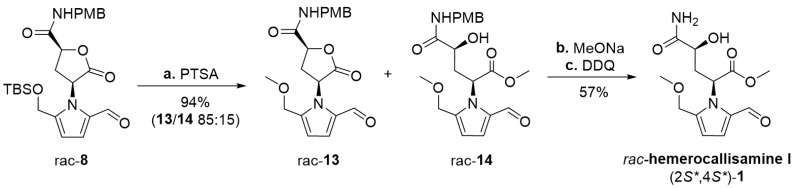
Synthesis of racemic hemerocallisamine I (**1**). Reagents and conditions: a) PTSA·H_2_O (2.0 equiv), CH_2_Cl_2_/MeOH 3:1, rt, 4 h. b) MeONa (0.3 equiv), dry MeOH, 0 °C, under argon, 30 min (quant); c) DDQ (3.0 equiv), CH_2_Cl_2_/phosp. buffer (pH 7) 5:1, rt, 24 h (57%). PMB = *p*-methoxybenzyl, TBS = *tert*-butyldimethylsilyl, PTSA = *p*-toluenesulfonic acid, DDQ = 2,3-dichloro-5,6-dicyano-1,4-benzoquinone.

Having 2-formylpyrrole rac-**8** in hand, the synthesis of racemic hemerocallisamine I (**1**) could proceed to the concluding steps (Scheme 3). Under conditions developed by Okada et al. [39] and later tailored by Brimble et al. [7], rac-**8** was simultaneously desilylated and converted into a mixture of methyl ethers rac-**13** and rac-**14** in a ratio of 85:15. The crude product was directly used in a subsequent lactone opening and oxidative cleavage of the PMB group, thus providing (±)-hemerocallisamine I, (2S*,4S*)-**1** (Scheme 3).

### 2.2. Synthesis of (–)-hemerocallisamine I

**Scheme 4:**
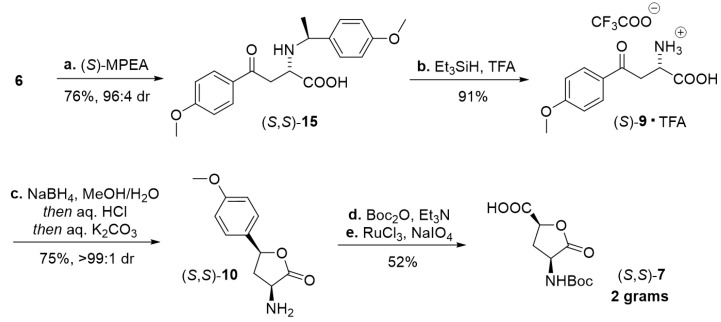
Preparation of 4-hydroxyglutamic acid lactone (*S*,*S*)-**7**. Reagents and conditions: (**a**) (*S*)-MPEA (1.1 equiv), MeOH, 40 °C, 5 d; (**b**) Et_3_SiH (1.0 equiv), TFA (10 equiv), 60 °C, 20 h; (**c**) NaBH_4_ (3.5 equiv), MeOH/H_2_O 5:1, rt, 90 min; then conc. HCl/H_2_O 1:2, rt, 4 h; then 10% aq. K_2_CO_3_/H_2_O 5:4; (**d**) Boc_2_O (1.1 equiv), Et_3_N (2.2 equiv), THF, rt, 4 h (81%); (**e**) 6 mol% RuCl_3_, NaIO_4_ (19.4 equiv), H_2_O/MeCN/AcOEt 3:1:1, 10 °C → rt, 3 h (64%). MPEA = 1-(4-methoxyphenyl)ethan-1-amine, TFA = trifluoroacetic acid, Boc = *tert*-butyloxycarbonyl.

With lessons learned from successfully completing the racemic sequence, we moved towards synthesizing the natural (–)-hemerocallisamine I. The initial steps were devoted to a stereoselective assembly of 4-hydroxyglutamic acid lactone (*S*,*S*)-**7** (Scheme 4).

Crystallization-induced diastereomer transformations (CIDT) built around aza-Michael additions have been proven to provide convenient access to diversely functionalized α-amino acids in a highly stereoselective fashion [27]. (*S*)-/(*R*)-Phenylethan-1-amine has frequently been reported as a chiral auxiliary. Herein we chose to slightly alter the original procedure [40] and utilize a suitably cleavable (*S*)-1-(4-methoxyphenyl)ethan-1-amine ((*S*)-MPEA) as a chiral amine carrier. CIDT with 1.1 equiv of (*S*)-MPEA was monitored by means of HPLC, proceeded over 5 days, and crystalline amino acid (*S*,*S*)-**15** was collected in 96:4 dr. The ensuing TFA-mediated acidolytic *N*-debenzylation in the presence of a silane scavenger [41] smoothly provided crystalline salt of (*S*)-**9**. At this point, the synthetic sequence previously elaborated on the racemic substrate **9** (Scheme 2) came into play, and after the reaction steps **c.**–**e.**, the 4-hydroxyglutamic acid lactone (*S*,*S*)-**7** was obtained in gram quantities. (Scheme 4). Considering that this five-step route could be readily repurposed to deliver the unnatural (*R*,*R*)-configuration, it makes a valuable addition to the available approaches [19,20,21,22,23,24,25,26].

**Scheme 5:**
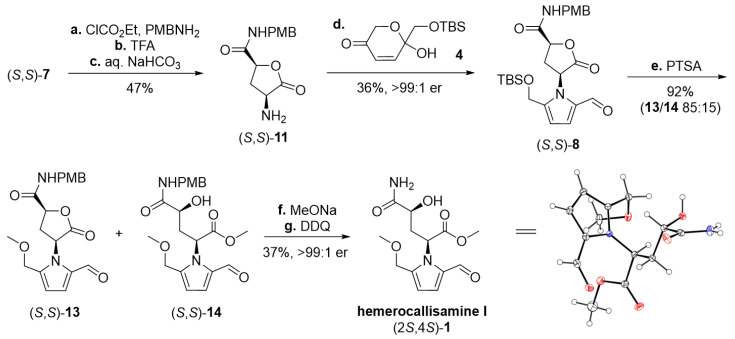
Synthesis of (–)-hemerocallisamine I (2*S*,4*S*)-(**1**). Reagents and conditions: (**a**) ClCO_2_Et (2.0 equiv), Et_3_N (2.5 equiv), dry THF, 15 min, 0 °C; then PMBNH_2_ (2.7 equiv), 0 °C, 2 h (51%); (**b**) TFA (10.6 equiv), CH_2_Cl_2_, 0 °C → rt, 3 h (95%); (**c**) 10% aq. NaHCO_3_, CH_2_Cl_2_, (96%); (**d**) **4** (1.0 equiv), dry toluene (0.2 M), 70 °C, under argon, 14 h; (**e**) PTSA·H_2_O (2.0 equiv), CH_2_Cl_2_/MeOH 3:1, rt, 4 h. (**f**) MeONa (0.4 equiv), dry MeOH, 0 °C, under argon, 30 min (93%); (**g**) DDQ (3.0 equiv), CH_2_Cl_2_/phosp. buffer (pH 7) 6:1, rt, 24 h (40%). PMB = *p*-methoxybenzyl, TFA = trifluoroacetic acid, TBS = *tert*-butyldimethylsilyl, PTSA = *p*-toluenesulfonic acid, DDQ = 2,3-dichloro-5,6-dicyano-1,4-benzoquinone.

Continuing with (*S*,*S*)-**7** and following the verified procedures (Table 1, Scheme 3), (–)-hemerocallisamine I (*S*,*S*)-(**1**) was obtained, albeit in lower yields (Scheme 5). As confirmed both for the Maillard product (*S*,*S*)-**8** and the target (*S*,*S*)-**1** itself, the stereochemical integrity of these species remained uncompromised (chiral HPLC traces of (*S*,*S*)-**8** and (*S*,*S*)-**1** are available in the Supplementary Materials). The relative configuration of (*S*,*S*)-**1** was confirmed by means of X-ray analysis^#^ (#: Crystallographic data for (–)-hemerocallisamine I (2S,4S)-(1) have been deposited with the Cambridge Crystallographic Data Centre (CCDC 2236255). Copies of the data can be obtained, free of charge, via https://www.ccdc.cam.ac.uk/structures/(accessed on 31 January 2023) (Scheme 5). The specific rotation of the synthesized (–)-hemerocallisamine I ([α]^25^_D_ -34.5 (*c* 0.12, MeOH)) matched exactly that of the natural product ([α]^25^_D_ -34.6 (MeOH)) [6] and was lower than that reported by Brimble et al. ([α]^21^_D_ -44.0 (*c* 0.1, MeOH)) [7]. Contrariwise, the mp value of our (*S*,*S*)-(**1**) sample (171.2–172.8 °C) was considerably higher than documented in the same paper (153.0–154.6 °C) [7].

## 3. Materials and Methods

### 3.1. General Experimental Details

Unless otherwise noted, all chemicals were purchased from commercial sources and used without further purifications. Column chromatography was carried out using silica 60 Å, Davisil, purchased from Fisher Chemicals. Reactions were monitored by thin-layer chromatography (TLC) using Macherey-Nagel’s pre-coated TLC sheets POLYGRAM SIL G/UV254, which were visualized under UV light (254 nm) or by staining with aqueous basic potassium permanganate or cerium molybdate solutions, as appropriate. HPLC analyses were performed on a Varian system using a Macherey-Nagel EC 250/4 Nucleodur Phenyl-Hexyl 5 μm, CHIRAL ART, Amylose-SA, 250 mm × 4.6 mm, 5 µm and Astec CHIROBIOTIC ^®^T, 250 mm × 4.6 mm, 5 µm column. All ^1^H and ^13^C NMR (Supplementary Materials) spectra were recorded using Bruker Avance NEO 400 MHz and/or Varian 400 MR spectrometers. Chemical shifts (δ) are given in parts per million (ppm). The ^1^H NMR chemical shift scale is referenced to the TMS internal standard (δ = 0 ppm) or solvent residual peak (δ = 2.50 ppm for DMSO-*d_6_* and δ = 7.26 ppm for CDCl_3_). The ^13^C NMR chemical shift scale is referenced to the solvent residual peak (δ = 39.52 ppm for DMSO-*d_6_* and δ = 77.16 ppm for CDCl_3_). Coupling constants (*J*) are given in hertz (Hz). The multiplicity of ^1^H NMR signals is reported as follows: s = singlet, d = doublet, t = triplet, q = quartet, m = multiplet, bs = broad singlet, “t” for dd with two identical or similar coupling constants, “dt” or “td” for ddd with two identical or similar coupling constants, and “q” for ddd with three identical or similar coupling constants. Optical rotations were recorded using the JASCO P-2000 polarimeter, with [α]_D_ values measured at 589 nm and the concentration (*c*) given in g/100 mL. High-resolution mass spectra were measured using a Thermo Scientific mass spectrometer with an Orbitrap analyzer and HESI ionization.

### 3.2. Synthesis and Characterization of Compounds

#### 3.2.1. 2-Amino-4-(4-methoxyphenyl)-4-oxobutanoic acid (**9**)

Acid **6** (14.7 g, 71.3 mmol) was dissolved in MeOH (120 mL) and aqueous ammonia (25–27% NH_3_ in H_2_O, 24 mL) was added. The mixture was stirred at rt, and the reaction progress was monitored by HPLC. After 16 h, the precipitated product was collected by filtration, washed with a small amount of Et_2_O, and dried in vacuo to provide amino acid **9** (12.3 g, 55.1 mmol, 77%) as a white powder. Mp 191.9–192.7 °C; ^1^H NMR (400 MHz, DMSO-*d_6_*): δ = 7.94 (d, *J* = 8.9 Hz, 2H), 7.60 (bs, 3H), 7.05 (d, *J* = 8.9 Hz, 2H), 3.85 (s, 3H), 3.62 (dd, *J* = 8.8, 3.4 Hz, 1H), 3.52 (dd, *J* = 18.2, 3.4 Hz, 1H), 3.27 (dd, *J* = 18.2, 8.8 Hz, 1H); ^13^C NMR (100 MHz, DMSO-*d_6_* + DCl): δ = 194.64, 170.32, 163.99, 130.82, 128.59, 114.41, 55.95, 47.92, 38.10; HRMS–HESI (*m*/*z*): calcd for C_11_H_14_NO_4_ [M + H]^+^, 224.09173, found 224.09174.

#### 3.2.2. (3S*,5S*)-3-Amino-5-(4-methoxyphenyl)dihydrofuran-2(3H)-one (rac-**10**)

Acid **9** (12.3 g, 55.1 mmol) was suspended in a mixture of MeOH (200 mL) and H_2_O (40 mL). NaBH_4_ (7.30 g, 0.193 mol, 3.5 equiv) was added portionwise over 30 min at rt. The reaction progress was monitored by HPLC. After 1 h, the reaction mixture was concentrated in vacuo, providing the crude hydroxy acid. The product was suspended in H_2_O (120 mL), and conc. HCl (36%, 60 mL) was added. The reaction mixture was stirred at rt for 4 h. After completion of the reaction, the insoluble white precipitate was collected by filtration and washed with 1 M HCl (15 mL) and Et_2_O (2 × 30 mL). The white solid was then suspended in H_2_O (80 mL), and 10% aqueous K_2_CO_3_ solution (100 mL) was added. The resulting mixture was extracted with CH_2_Cl_2_ (3 × 150 mL). The combined organic layers were dried over anhydrous Na_2_SO_4_ and concentrated in vacuo, yielding amino lactone rac-**10** (9.5 g, 45.8 mmol, 83%, *cis*:*trans* > 99:1) as a white powder. Rf 0.36 (EtOAc:MeOH, 8:2); mp 102.4–105.4 °C; ^1^H NMR (400 MHz, CDCl_3_): δ = 7.31–7.27 (m, 2H), 6.94–6.90 (m, 2H), 5.30 (dd, *J* = 11.0, 5.2 Hz, 1H), 3.90 (dd, *J* = 12.1, 8.0 Hz, 1H), 3.82 (s, 3H), 2.89 (ddd, *J* = 13.0, 8.0, 5.2 Hz, 1H), 2.02 (“td”, *J* = 12.4, 11.0 Hz, 1H), 1.67 (s, 2H); ^13^C NMR (100 MHz, DMSO-*d_6_*): δ = 178.74, 159.39, 130.85, 128.01, 113.87, 77.01, 55.12, 52.36, 39.73; HRMS–HESI (*m*/*z*): calcd for C_11_H_14_NO_3_ [M + H]^+^, 208.09682, found 208.09689.

#### 3.2.3. (2S*,4S*)-4-((tert-Butoxycarbonyl)amino)-5-oxotetrahydrofuran-2-carboxylic acid (rac-**7**)

Step **c**: Lactone rac-**10** (8.4 g, 40.5 mmol) was dissolved in THF (405 mL) and Et_3_N (12.4 mL, 89.2 mmol, 2.2 equiv) was added. Boc_2_O (9.73 g, 44.6 mmol, 1.1 equiv) was dissolved in a small amount of THF (15 mL) and the resulting solution was added to the first one. The reaction mixture was stirred at rt and monitored by TLC. After 4 h, the reaction mixture was cooled to 0 °C and the pH was adjusted to 2–3 with 2 M HCl, followed by extraction with EtOAc (3 × 140 mL). The combined organic layers were washed with brine (200 mL), dried over anhydrous Na_2_SO_4,_ and concentrated in vacuo. The crude product was crystallized from a mixture of EtOAc and heptane, yielding the corresponding *N*-Boc lactone (11.7 g, 38.1 mmol, 94%) as a white solid. Rf 0.69 (EtOAc); mp 175.6–176.2 °C; ^1^H NMR (400 MHz, DMSO-*d_6_*): δ = 7.39 (d, *J* = 8.6 Hz, 1H), 7.34 (d, *J* = 8.7 Hz, 2H), 6.98 (d, *J* = 8.7 Hz, 2H), 5.37 (dd, *J* = 11.0, 5.6 Hz, 1H), 4.56 (“dt”, *J* = 11.9, 8.6 Hz, 1H), 3.76 (s, 3H), 2.70 (ddd, *J* = 11.9, 8.6, 5.6 Hz, 1H), 2.20 (“q”, *J* = 11.7 Hz, 1H), 1.41 (s, 9H); ^13^C NMR (100 MHz, DMSO-*d_6_*): δ = 174.88, 159.53, 155.03, 130.82, 128.04, 114.00, 78.56, 77.26, 55.18, 50.90, 36.19, 28.13; HRMS–HESI (*m*/*z*): calcd for C_16_H_21_NO_5_Na [M + Na]^+^, 330.13119, found 330.13140.

Step **d**: The *N*-Boc lactone (4.0 g, 13.0 mmol) was dissolved in a mixture of CH_3_CN (65 mL) and EtOAc (65 mL). In parallel, NaIO_4_ (54.1 g, 252.7 mmol, 19.4 equiv) was dissolved in H_2_O (184 mL) and both solutions were combined. Consequently, RuCl_3_ (162 mg, 0.782 mmol, 0.06 equiv) was added and the reaction mixture was stirred at 10 °C in a water bath. After 1 h, the bath was removed, and the reaction mixture was stirred for an additional 2 h at rt. Then the resulting thick suspension was decanted, and the white residue was washed with EtOAc (4 × 100 mL). Et_2_O (50 mL) was added to the combined solutions and the resulting mixture was stirred for 30 min at rt. Afterwards, the resulting black suspension was filtered through a pad of Celite, and the pad was washed with EtOAc (3 × 50 mL). The combined filtrate was washed with 20% aqueous NaCl (3 × 100 mL) and dried over anhydrous Na_2_SO_4_. The filtrate volume was reduced in vacuo to *ca* 10 mL, and Et_2_O (30 mL) was added. The resulting suspension was placed in a freezer for 30 min. The white precipitate was collected by filtration, washed with a small amount of Et_2_O, and dried in vacuo*,* yielding the desired acid rac-**7** (2.4 g, 9.79 mmol, 75%) as a white solid. Rf 0.22 (EtOAc:MeOH:AcOH, 7:3:0.1); mp 166.9–169.5 °C; ^1^H NMR (400 MHz, DMSO-*d_6_*): δ = 13.35 (bs, 1H), 7.36 (d, *J* = 8.5 Hz, 1H), 4.89 (dd, *J* = 10.9, 6.5 Hz, 1H), 4.50 (“dt”, *J* = 11.8, 8.7 Hz, 1H), 2.66 (ddd, *J* = 11.9, 8.9, 6.5 Hz, 1H), 2.12 (“q”, *J* = 11.6 Hz, 1H), 1.38 (s, 9H); ^13^C NMR (100 MHz, DMSO-*d_6_*): δ = 174.20, 170.23, 155.00, 78.71, 72.21, 49.75, 31.21, 28.12; HRMS–HESI (*m*/*z*): calcd for C_10_H_15_NO_6_Na [M + Na]^+^, 268.07916, found 268.07923.

#### 3.2.4. (2S*,4S*)-4-Amino-N-(4-methoxybenzyl)-5-oxotetrahydrofuran-2-carboxamide (rac-**11**)

Step **e**: Acid rac-**7** (1.43 g, 5.83 mmol) was dissolved in dry THF (145 mL) at 0 °C and the solution was treated with Et_3_N (1.48 g, 2.0 mL, 14.6 mmol, 2.5 equiv). Ethyl chloroformate (1.30 g, 1.1 mL, 11.7 mmol, 2.0 equiv) was added dropwise at 0 °C, under argon. After 15 min, *para*-methoxybenzyl amine (2.20 g, 2.1 mL, 15.7 mmol, 2.7 equiv) was added and the reaction mixture was stirred at 0 °C for 2 h, under argon. After completion, the reaction mixture was diluted with EtOAc (100 mL) and washed with 1M HCl (50 mL). The organic layer was dried over anhydrous Na_2_SO_4_ and concentrated under reduced pressure, yielding a pale-yellow solid. The crude product was treated with MeOH (5 mL), resulting in a white suspension that was cooled to 0 °C. The white precipitate was collected by filtration, washed with ice-cold MeOH, and dried in vacuo*,* yielding the corresponding amide (1.4 g, 3.84 mmol, 66%) as a white solid. Rf 0.48 (EtOAc); mp 192.6–194.3 °C; ^1^H NMR (400 MHz, DMSO-*d_6_*): δ = 8.63 (“t”, *J* = 6.1 Hz, 1H), 7.38 (d, *J* = 8.5 Hz, 1H), 7.22–7.17 (m, 2H), 6.90–6.84 (m, 2H), 4.82 (dd, *J* = 10.6, 6.4 Hz, 1H), 4.51 (“dt”, *J* = 11.7, 8.7 Hz, 1H), 4.28 (dd, *J* = 14.7, 6.2 Hz, 1H), 4.20 (dd, *J* = 14.7, 5.9 Hz, 1H), 3.72 (s, 3H), 2.62 (ddd, *J* = 12.0, 8.9, 6.4 Hz, 1H), 2.17–2.06 (m, 1H), 1.39 (m, 9H); ^13^C NMR (100 MHz, DMSO-*d_6_*): δ = 174.31, 168.04, 158.24, 155.02, 130.98, 128.59, 113.65, 78.66, 73.83, 55.04, 49.79, 41.37, 31.50, 28.10; HRMS–HESI (*m*/*z*): calcd for C_18_H_24_N_2_O_6_Na [M + Na]^+^, 387.15266, found 387.15270.

Step **f**: The amide (580 mg, 1.59 mmol) was dissolved in CH_2_Cl_2_ (6.4 mL), the solution was cooled to 0 °C, and trifluoroacetic acid (1.92 g, 1.3 mL, 16.8 mmol, 10.6 equiv) was added. The reaction mixture was stirred at rt for 3 h while being monitored by TLC. The mixture was concentrated in vacuo, Et_2_O (10–15 mL) was added to the residue, and the mixture was kept in the ultrasonic bath for 30 min to form a white suspension. The white solid was filtered off, washed with a small amount of Et_2_O (5 mL), and dried in vacuo*,* providing rac-**11∙**TFA (574 mg, 1.52 mmol, 95%) as an off-white solid. Rf 0.26 (EtOAc:MeOH, 8:2), mp 94.0–96.2 °C; ^1^H NMR (400 MHz, DMSO-*d_6_*): δ = 8.84 (“t”, *J* = 6.0 Hz, 1H), 8.63 (bs, 3H), 7.23–7.17 (m, 2H), 6.91–6.86 (m, 2H), 4.96 (dd, *J* = 10.3, 6.2 Hz, 1H), 4.48 (dd, *J* = 11.5, 8.9 Hz, 1H), 4.29 (dd, *J* = 14.7, 6.0 Hz, 1H), 4.22 (dd, *J* = 14.7, 5.9 Hz, 1H), 3.73 (s, 3H), 2.78 (ddd, *J* = 12.2, 9.0, 6.2 Hz, 1H), 2.22 (“td”, *J* = 11.9, 10.3 Hz, 1H); ^13^C NMR (100 MHz, DMSO-*d_6_*): δ = 172.06, 167.04, 158.37, 130.74, 128.77, 113.75, 74.93, 55.08, 48.41, 41.62, 30.38.

Step **g**: Salt rac-**11∙**TFA (570 mg, 1.51 mmol) was suspended in CH_2_Cl_2_ (15 mL), and the resulting suspension was washed with 10% aqueous NaHCO_3_ (7 mL). The aqueous layer was extracted with CH_2_Cl_2_ (2 × 10 mL). The combined organic layers were dried over anhydrous Na_2_SO_4_ and concentrated in vacuo*,* providing rac-**11** (360 mg, 1.36 mmol, 90%) as a white solid. Rf 0.26 (EtOAc:MeOH, 8:2); mp 105.8–107.5 °C; ^1^H NMR (400 MHz, DMSO-*d_6_*): δ = 8.64 (t, *J* = 6.0 Hz, 1H), 7.21–7.17 (m, 2H), 6.91–6.86 (m, 2H), 4.74 (dd, *J* = 10.1, 6.3 Hz, 1H), 4.23 (d, *J* = 6.0 Hz, 2H), 3.76–3.66 (m, 4H), 2.64 (ddd, *J* = 12.2, 8.5, 6.3 Hz, 1H), 2.01 (bs, 2H), 1.83 (ddd, *J* = 12.2, 11.0, 10.1 Hz, 1H); ^13^C NMR (100 MHz, DMSO-*d_6_*): δ = 178.16, 168.56, 158.28, 130.95, 128.70, 113.71, 73.95, 55.06, 50.98, 41.42, 35.12; HRMS–HESI (*m*/*z*): calcd for C_13_H_17_N_2_O_4_ [M + H]^+^, 265.11828, found 265.11842.

#### 3.2.5. (2S*,4S*)-4-(2-(((tert-Butyldimethylsilyl)oxy)methyl)-5-formyl-1H-pyrrol-1-yl)-N-(4-methoxybenzyl)-5-oxotetrahydrofuran-2-carboxamide (rac-**8**)

Amide rac-**11** (200 mg, 0.757 mmol) was dissolved in dry toluene (3.8 mL) and dihydropyranone **4** (196 mg, 0.757 mmol, 1.0 equiv) was added. The reaction mixture was stirred at 70 °C for 14 h, under argon. The formation of a precipitate was observed. The reaction progress was monitored by TLC. After completion, the reaction mixture was concentrated in vacuo and purified by flash column chromatography (petroleum ether:EtOAc, 1:1), providing rac-**8** (158 mg, 0.325 mmol, 43%) as a pale-yellow oil. Rf 0.42 (petroleum ether:EtOAc, 1:1); ^1^H NMR (400 MHz, CDCl_3_): δ = 9.34 (s, 1H), 7.40 (“t”, *J* = 6.1 Hz, 1H), 7.32–7.27 (m, 2H), 7.00 (d, *J* = 4.0 Hz, 1H), 6.89–6.84 (m, 2H), 6.20 (d, *J* = 4.0 Hz, 1H), 5.45 (dd, *J* = 11.4, 9.4 Hz, 1H), 4.94 (dd, *J* = 10.3, 7.6 Hz, 1H), 4.77 (d, *J* = 13.6 Hz, 1H), 4.69 (d, *J* = 13.6 Hz, 1H), 4.57 (dd, *J* = 14.6, 6.3 Hz, 1H), 4.43 (dd, *J* = 14.7, 5.4 Hz, 1H), 3.80 (s, 3H), 3.06 (ddd, *J* = 12.4, 9.5, 7.6 Hz, 1H), 2.57 (“q”, *J* = 11.4 Hz, 1H), 0.88 (s, 9H), 0.09 (s, 3H), 0.04 (s, 3H); ^13^C NMR (100 MHz, CDCl_3_): δ = 178.93, 171.02, 168.80, 159.15, 142.76, 131.60, 129.78, 129.29, 126.15, 114.16, 110.75, 74.72, 57.52, 55.42, 55.27, 42.94, 33.08, 25.90, 18.33, -5.08, -5.16; HRMS–HESI (*m*/*z*): calcd for C_25_H_44_N_2_O_6_SiNa [M + Na]^+^, 509.20783, found 509.20805.

#### 3.2.6. (2S*,4S*)-4-(2-Formyl-5-(methoxymethyl)-1H-pyrrol-1-yl)-N-(4-methoxybenzyl)-5-oxotetrahydrofuran-2-carboxamide (rac-**13**)

2-Formylpyrrole rac-**8** (278 mg, 0.571 mmol) was dissolved in CH_2_Cl_2_ (8.6 mL) and a solution of *para*-toluenesulfonic acid monohydrate (217 mg, 1.14 mmol, 2.0 equiv) in MeOH (2.9 mL) was added. The resulting mixture was stirred for 4 h at rt, while being monitored by TLC. After completion, the reaction mixture was neutralized with a saturated aqueous NaHCO_3_ (6 mL), and the aqueous layer was extracted with CH_2_Cl_2_ (2 × 15 mL). The combined organic layers were washed with a saturated brine solution (15 mL), dried over anhydrous Na_2_SO_4_, and concentrated in vacuo. The resulting crude product was purified by flash column chromatography (EtOAc:petroleum ether 3:1 → 4:1), providing a white solid (209 mg, 0.534 mmol for mole-fraction-weighted M = 391.21 g.mol^−1^, 94%). The isolated product contained 85% of rac-**13** and 15% of rac-**14**, as confirmed by HPLC and ^1^H NMR. This mixture was used in the next step.

Isolation of rac-**13**: The mixture of rac-**13** and rac-**14** (85:15) was treated with MeOH (3 mL), resulting in a white suspension that was placed in a freezer. The white precipitate that formed was collected by filtration, providing lactone rac-**13** (105 mg, 0.272 mmol, 47%) as a white solid. The filtrate contained a mixture of rac-**13** and rac-**14** (1:1) according to HPLC. Rf 0.39 (petroleum ether:EtOAc, 1:3); mp 131.5–133.6 °C; ^1^H NMR (400 MHz, CDCl_3_): δ = 9.36 (s, 1H), 7.37–7.33 (m, 1H), 7.32–7.27 (m, 2H), 7.03 (d, *J* = 4.0 Hz, 1H), 6.90–6.84 (m, 2H), 6.30 (d, *J* = 4.0 Hz, 1H), 5.39 (dd, *J* = 11.4, 9.5 Hz, 1H), 4.95 (dd, *J* = 10.4, 7.6 Hz, 1H), 4.62–4.53 (m, 2H), 4.51–4.40 (m, 2H), 3.80 (s, 3H), 3.31 (s, 3H), 3.07 (ddd, *J* = 12.4, 9.5, 7.6 Hz, 1H), 2.55 (ddd, *J* = 12.5, 11.5, 10.4 Hz, 1H); ^13^C NMR (100 MHz, CDCl_3_): δ = 178.88, 170.86, 168.56, 159.01, 139.60, 131.83, 129.58, 129.14, 125.75, 114.01, 112.45, 74.56, 65.69, 57.67, 55.27, 55.10, 42.77, 32.91; HRMS–HESI (*m*/*z*): calcd for C_20_H_22_N_2_O_6_Na [M + Na]^+^, 409.13701, found 409.13717.

#### 3.2.7. (±)-Hemerocallisamine I, (2S*,4S*)-**1**

Step **b**: The mixture of rac-**13** and rac-**14** (85:15) (100 mg, 0.256 mmol for mole-fraction-weighted M = 391.21 g.mol^−1^) was dissolved in dry MeOH (2.9 mL). MeONa (25w% solution in MeOH) (4.2 mg, 21 μL, 0.078 mmol, 0.3 equiv) was added to the solution at 0 °C, under an argon atmosphere. The reaction mixture was stirred at 0 °C and was monitored by TLC. After 30 min, a saturated aqueous NH_4_Cl solution (2 mL) was added at 0 °C and the resulting mixture was extracted with EtOAc (3 × 10 mL). The collected organic layers were washed with brine (10 mL), dried over anhydrous Na_2_SO_4_, and concentrated in vacuo*,* yielding rac-**14** as a thick pale-yellow oil (108 mg, quantitative).

Step **c**: The crude methyl ester rac-**14** (100 mg, 0.239 mmol) was dissolved in a mixture of CH_2_Cl_2_ (2.3 mL) and a phosphate buffer (NaH_2_PO_4_, Na_2_HPO_4_; pH 7, *c* 1M; 0.481 mL). Subsequently, 2,3-dichloro-5,6-dicyano-1,4-benzoquinone (163 mg, 0.717 mmol, 3.0 equiv) was added to the solution. The resulting mixture was stirred at rt while the reaction progress was monitored by HPLC and TLC. After 24 h, the reaction mixture was diluted with CH_2_Cl_2_ (5 mL), dried with Na_2_SO_4_, and filtered through a Celite pad. The pad was washed first with CH_2_Cl_2_ (10 mL), then with dioxane (3 × 20 mL). The combined filtrates were concentrated in vacuo and purified using flash column chromatography (CH_2_Cl_2_:MeOH, 13:1). The desired product was isolated after trituration from MeOH–Et_2_O, yielding (±)-hemerocallisamine I ((2S*,4S*)-**1**) (41 mg, 0.137 mmol, 57%) as a white solid. Mp 204.3–204.9 °C; ^1^H NMR (400 MHz, DMSO-*d_6_*): δ = 9.37 (s, 1H), 7.25 (s, 1H), 7.18–7.11 (m, 2H), 6.33 (d, *J* = 3.9 Hz, 1H), 5.74–5.66 (m, 1H), 5.27 (bs, 1H), 4.74 (d, *J* = 13.3 Hz, 1H), 4.37 (d, *J* = 13.2 Hz, 1H), 3.57 (s, 3H), 3.20 (s, 3H), 3.08–3.00 (m, 1H), 2.42–2.26 (m, 2H); ^13^C NMR (100 MHz, DMSO-*d_6_*): δ = 179.38, 176.32, 170.26, 141.05, 132.43, 126.44, 111.54, 69.70, 65.31, 57.50, 55.69, 52.63, 37.06; HRMS–HESI (*m*/*z*): calcd for C_13_H_18_N_2_O_6_Na [M + Na]^+^, 321.10571, found 321.10584.

#### 3.2.8. (*S*)-4-(4-Methoxyphenyl)-2-(((*S*)-1-(4-methoxyphenyl)ethyl)amino)-4-oxobutanoic acid, (*S*,*S*)-**15**

Acid **6** (11.8 g, 57.2 mmol) was dissolved in MeOH (228 mL) and, subsequently, (*S*)-(–)-1-(4-methoxyphenyl)ethan-1-amine (9.52 g, 9.3 mL, 63.0 mmol, 1.1 equiv) was added dropwise. The reaction mixture was stirred at 40 °C while being monitored by HPLC. After 5 days, the white precipitate was collected by filtration, washed with a small amount of Et_2_O, and dried in vacuo*,* providing (*S*,*S*)-**15** (15.6 g, 43.7 mmol, 76%, dr 96:4) as a white solid. Mp 180.3–181.1 °C; [α]^25^_D_ +67.1 (*c* 1.00, MeOH:5% aq. HCl, 9:1); ^1^H NMR (400 MHz, acetone + DCl): δ = 7.95–7.91 (m, 2H), 7.71–7.65 (m, 2H), 7.03–6.94 (m, 4H), 4.83 (q, *J* = 6.9 Hz, 1H), 4.12 (t, *J* = 5.3 Hz, 1H), 3.96 (d, *J* = 5.1 Hz, 2H), 3.84 (s, 3H), 3.80 (s, 3H), 1.84 (d, *J* = 6.9 Hz, 3H); ^13^C NMR (100 MHz, acetone + DCl): δ = 194.83, 169.69, 164.91, 161.26, 131.50, 130.87, 129.40, 128.31, 115.28, 114.67, 59.19, 56.01, 55.62, 53.82, 39.17, 20.81; HRMS–HESI (*m*/*z*): calcd for C_20_H_24_NO_5_ [M + H]^+^, 358.16490, found 358.16496.

#### 3.2.9. (*S*)-1-Carboxy-3-(4-methoxyphenyl)-3-oxopropan-1-aminium 2,2,2-trifluoroacetate, (*S*)-**9**∙TFA

Amino acid (*S*,*S*)-**15** (4.37 g, 12.2 mmol) was dissolved in trifluoroacetic acid (9.4 mL, 13.9 g, 122.3 mmol, 10.0 equiv) and triethylsilane (1.9 mL, 1.42 g, 12.2 mmol, 1.0 equiv) was added. The resulting solution was stirred at 60 °C and the reaction was monitored by HPLC. After 20 h, the reaction mixture was concentrated in vacuo, and the residue was treated with Et_2_O (20 mL) and placed in an ultrasonic bath for 10 min. The insoluble white solid was filtered off, washed with a small amount of Et_2_O, and dried in vacuo to give (*S*)-**9∙**TFA (3.75 g, 11.1 mmol, 91%) as a white solid. Mp 155.0–156.0 °C; [α]^25^_D_ +24.3 (*c* 1.00, MeOH); ^1^H NMR (400 MHz, DMSO-*d_6_*): δ = 8.34 (bs, 3H), 8.00–7.95 (m, 2H), 7.12–7.03 (m, 2H), 4.31 (t, *J* = 5.1 Hz, 1H), 3.85 (s, 3H), 3.62 (d, *J* = 5.1 Hz, 2H); ^13^C NMR (100 MHz, DMSO-*d_6_*): δ = 194.44, 170.53, 163.75, 130.56, 128.48, 114.14, 55.68, 48.08, 38.16; HRMS–HESI (*m*/*z*): calcd for C_11_H_14_NO_4_ [M + H]^+^, 224.09173, found 224.09176.

#### 3.2.10. (3*S*,5*S*)-3-Amino-5-(4-methoxyphenyl)dihydrofuran-2(3H)-one, (*S*,*S*)-**10**

The salt (*S*)-**9∙**TFA (13.0 g, 38.5 mmol) was suspended in a mixture of MeOH (128 mL) and H_2_O (28 mL). NaBH_4_ (5.1 g, 0.135 mol, 3.5 equiv) was added portionwise over 30 min to the suspension at rt. The reaction was monitored by HPLC. After 1 h, the reaction mixture was concentrated in vacuo*,* providing the crude hydroxy acid. The crude product was suspended in H_2_O (85 mL), and conc. HCl (36%, 45 mL) was added. The reaction mixture was stirred at room temperature for 4 h. After completion of the reaction, the insoluble white precipitate was collected by filtration and washed with 1M HCl (15 mL) and Et_2_O (2 × 30 mL). The white solid was then suspended in H_2_O (80 mL), and a 10% aqueous K_2_CO_3_ solution (100 mL) was added. The resulting mixture was extracted with CH_2_Cl_2_ (3 × 100 mL). The combined organic layers were dried over anhydrous Na_2_SO_4_ and concentrated in vacuo*,* providing amino lactone (*S*,*S*)-**10** (6.0 g, 29.0 mmol, 75%, *cis*:*trans* > 99:1) as a white powder. Mp 101.8–103.0 °C; [α]^25^_D_ -1.3 (*c* 1.00, MeOH); ^1^H NMR (400 MHz, CDCl_3_): δ = 7.31–7.25 (m, 2H), 6.95–6.87 (m, 2H), 5.29 (dd, *J* = 11.0, 5.2 Hz, 1H), 3.89 (dd, *J* = 12.1, 8.0 Hz, 1H), 3.81 (s, 3H), 2.89 (ddd, *J* = 12.5, 8.0, 5.2 Hz, 1H), 2.02 (ddd, *J* = 12.6, 12.2, 11.0 Hz, 1H, 1H), 1.68 (bs, 2H).

#### 3.2.11. (2*S*,4*S*)-4-((*tert*-Butoxycarbonyl)amino)-5-oxotetrahydrofuran-2-carboxylic acid, (*S*,*S*)-**7**

Step **d**: The lactone (*S*,*S*)-**10** (5.5 g, 26.5 mmol) was dissolved in THF (265 mL), and Et_3_N (8.1 mL, 5.91 g, 58.4 mmol, 2.2 equiv) was added. Boc_2_O (6.37 g, 29.2 mmol, 1.1 equiv) was dissolved in a small amount of THF (10 mL) and the resulting solution was added to the first one. The reaction mixture was stirred at room temperature and was monitored by TLC. After 4 h, the reaction mixture was cooled to 0 °C and the pH was adjusted to 2–3 with 2M HCl, followed by extraction with EtOAc (3 × 90 mL). The combined organic layers were washed with brine (130 mL), dried over anhydrous Na_2_SO_4_,_,_ and concentrated in vacuo*,* yielding crude *N*-Boc lactone as a pale-yellow solid. The crude product was crystallized from EtOAc-heptane, providing the *N*-Boc lactone (6.6 g, 21.5 mmol, 81%) as white crystals. Mp 189.7–190.9 °C; [α]^25^_D_ +9.3 (*c* 1.00, MeOH); ^1^H NMR (400 MHz, DMSO-*d_6_*): δ = 7.39 (d, *J* = 8.6 Hz, 1H), 7.36–7.31 (m, 2H), 7.02–6.95 (m, 2H), 5.37 (dd, *J* = 11.0, 5.6 Hz, 1H), 4.56 (dt, *J* = 11.9, 8.6 Hz, 1H), 3.76 (s, 3H), 2.70 (ddd, *J* = 12.0, 8.6, 5.6 Hz, 1H), 2.21 (“q”, *J* = 11.8 Hz, 1H), 1.41 (s, 9H).

Step **e**: The *N*-Boc lactone (4.0 g, 13.0 mmol) was dissolved in a mixture of CH_3_CN (65 mL) and EtOAc (65 mL). In parallel, NaIO_4_ (54.1 g, 252.7 mmol, 19.4 equiv) was dissolved in H_2_O (184 mL) and both solutions were combined. Consequently, RuCl_3_ (162 mg, 0.782 mmol, 0.06 equiv) was added, and the reaction mixture was stirred in a water bath (10 °C). After 1 h, the bath was removed, and the reaction mixture was stirred for an additional 2 h at rt. The thick suspension was then decanted, and the white residue was washed with EtOAc (4 × 100 mL). Et_2_O (70 mL) was added to the combined solutions, and the resulting mixture was stirred for 30 min at rt. Afterward, the resulting black suspension was filtered through the pad of Celite, and the pad was washed with EtOAc (3 × 50 mL). The combined filtrate was washed with a 20% aqueous NaCl solution (3 × 100 mL), dried over anhydrous Na_2_SO_4,_ and concentrated in vacuo to provide (*S*,*S*)-**7** (2.04 g, 8.32 mmol, 64%) as a pale-yellow solid. Mp 171.7–174.6 °C; [α]^25^_D_ -1.4 (*c* 1.00, MeOH); ^1^H NMR (400 MHz, DMSO-*d_6_*): δ = 7.36 (d, *J* = 8.5 Hz, 1H), 4.88 (dd, *J* = 10.9, 6.5 Hz, 1H), 4.49 (“dt”, *J* = 11.7, 8.7 Hz, 1H), 2.66 (ddd, *J* = 11.8, 8.9, 6.6 Hz, 1H), 2.12 (“q”, *J* = 11.6 Hz, 1H), 1.38 (s, 9H).

#### 3.2.12. (2*S*,4*S*)-4-Amino-*N*-(4-methoxybenzyl)-5-oxotetrahydrofuran-2-carboxamide, (*S*,*S*)-**11**

Step **a**: Acid (*S*,*S*)-**7** (1.23 g, 5.02 mmol) was dissolved in dry THF (125 mL) at 0 °C and the solution was treated with Et_3_N (1.27 g, 1.7 mL, 12.5 mmol, 2.5 equiv). Ethyl chloroformate (1.12 g, 1.0 mL, 10.0 mmol, 2.0 equiv) was added dropwise at 0 °C, under argon. After 15 min, *para*-methoxybenzyl amine (1.90 g, 1.8 mL, 13.5 mmol, 2.7 equiv) was added and the reaction mixture was stirred at 0 °C for 2 h. After completion, the reaction mixture was diluted with EtOAc (85 mL) and washed with 1M HCl (1 × 40 mL). The aqueous phase was extracted with EtOAc (2 × 50 mL). The combined organic layers were dried over anhydrous Na_2_SO_4_ and concentrated under reduced pressure. The crude product was purified by flash column chromatography (EtOAc:Hex, 2:3 → 1:1→ 4:1), yielding the corresponding amide (930 mg, 2.55 mmol, 51%) as a pale-yellow solid. Mp 123.5–125.6 °C; [α]^25^_D_ +1.5 (*c* 1.00, MeOH); ^1^H NMR (400 MHz, DMSO-*d_6_*): δ = 8.63 (“t”, *J* = 6.1 Hz, 1H), 7.38 (d, *J* = 8.6 Hz, 1H), 7.22–7.17 (m, 2H), 6.90–6.85 (m, 2H), 4.82 (dd, *J* = 10.5, 6.2 Hz, 1H), 4.51 (“dt”, *J* = 11.7, 8.7 Hz, 1H), 4.28 (dd, *J* = 14.8, 6.3 Hz, 1H), 4.20 (dd, *J* = 14.9, 6.0 Hz, 1H), 3.72 (s, 3H), 2.61 (ddd, *J* = 11.8, 8.7, 6.3 Hz, 1H), 2.11 (“q”, *J* = 11.5 Hz, 1H), 1.39 (m, 9H).

Step **b**: The amide (650 mg, 1.78 mmol) was dissolved in CH_2_Cl_2_ (7.2 mL), the solution was cooled to 0 °C, and trifluoroacetic acid (2.15 g, 1.4 mL, 18.9 mmol, 10.6 equiv) was added. The reaction mixture was stirred at rt while being monitored by TLC. After 3 h, the mixture was concentrated in vacuo, Et_2_O (40 mL) was added to the residue, and the mixture was kept in the ultrasonic bath for 30 min to form a white suspension. The insoluble precipitate was filtered off, washed with a small amount of Et_2_O (5 mL), and dried in vacuo*,* yielding (*S*,*S*)-**11∙**TFA (643 mg, 1.70 mmol, 95%) as an off-white solid. Mp 133.6–135.1 °C; [α]^25^_D_ +14.6 (*c* 0.50, MeOH); ^1^H NMR (400 MHz, DMSO-*d_6_*): δ = 8.84 (“t”, *J* = 6.0 Hz, 1H), 8.56 (bs, 3H), 7.23–7.17 (m, 2H), 6.92–6.86 (m, 2H), 4.96 (dd, *J* = 10.3, 6.2 Hz, 1H), 4.47 (dd, *J* = 11.5, 8.9 Hz, 1H), 4.29 (dd, *J* = 14.7, 6.0 Hz, 1H), 4.23 (dd, *J* = 14.6, 5.9 Hz, 1H), 3.73 (s, 3H), 2.78 (ddd, *J* = 12.2, 8.9, 6.2 Hz, 1H), 2.26–2.10 (m, 1H).

Step **c**: Salt (*S*,*S*)-**11∙**TFA (643 mg, 1.70 mmol) was suspended in CH_2_Cl_2_ (20 mL), and the resulting suspension was washed with 10% aqueous NaHCO_3_ (5 mL). The aqueous layer was extracted with CH_2_Cl_2_ (2 × 10 mL). The combined organic layers were dried over anhydrous Na_2_SO_4_, and concentrated in vacuo*,* yielding (*S*,*S*)-**11** (430 mg, 1.63 mmol, 96%) as an orange solid. [α]^25^_D_ +14.0 (*c* 1.00, MeOH); ^1^H NMR (400 MHz, DMSO-*d_6_*): δ = 8.63 (t, *J* = 6.0 Hz, 1H), 7.22–7.15 (m, 2H), 6.91–6.86 (m, 2H), 4.74 (dd, *J* = 10.1, 6.3 Hz, 1H), 4.23 (d, *J* = 6.0 Hz, 2H), 3.73 (s, 3H), 3.72–3.67 (m, 1H), 2.64 (ddd, *J* = 12.2, 8.5, 6.3 Hz, 1H), 2.02 (bs, 2H), 1.83 (ddd, *J* = 12.2, 11.0, 10.1 Hz, 1H).

#### 3.2.13. (2*S*,4*S*)-4-(2-(((*tert*-Butyldimethylsilyl)oxy)methyl)-5-formyl-1H-pyrrol-1-yl)-*N*-(4-methoxybenzyl)-5-oxotetrahydrofuran-2-carboxamide, (*S*,*S*)-**8**

Amide (*S*,*S*)-**11** (200 mg, 0.757 mmol) was dissolved in dry toluene (3.8 mL), and dihydropyranone **4** (196 mg, 0.757 mmol, 1.0 equiv) was added. The reaction mixture was stirred at 70 °C for 14 h under argon. The formation of a precipitate was observed. The reaction progress was monitored by TLC. After completion, the reaction mixture was concentrated in vacuo and purified by flash column chromatography (petroleum ether:EtOAc, 1:1), providing (*S*,*S*)-**8** (131 mg, 0.269 mmol, 36%, er > 99:1) as a pale-yellow oil. [α]^25^_D_ +11.7 (*c* 0.70, MeOH); ^1^H NMR (400 MHz, CDCl_3_): δ = δ 9.34 (s, 1H), 7.40 (“t”, *J* = 5.6 Hz, 1H), 7.30 (d, *J* = 8.6 Hz, 2H), 7.00 (d, *J* = 4.0 Hz, 1H), 6.89–6.40 (m, 2H), 6.20 (d, *J* = 4.0 Hz, 1H), 5.45 (dd, *J* = 11.3, 9.5 Hz, 1H), 4.94 (dd, *J* = 10.3, 7.6 Hz, 1H), 4.77 (d, *J* = 13.6 Hz, 1H), 4.69 (d, *J* = 13.7 Hz, 1H), 4.57 (dd, *J* = 14.7, 6.3 Hz, 1H), 4.43 (dd, *J* = 14.7, 5.4 Hz, 1H), 3.80 (s, 3H), 3.06 (ddd, *J* = 12.5, 9.5, 7.6 Hz, 1H), 2.57 (ddd, *J* = 12.4, 11.3, 10.3 Hz, 1H), 0.88 (s, 9H), 0.09 (s, 3H), 0.04 (s, 3H).

#### 3.2.14. (2*S*,4*S*)-4-(2-Formyl-5-(methoxymethyl)-1H-pyrrol-1-yl)-*N*-(4-methoxybenzyl)-5-oxotetrahydrofuran-2-carboxamide, (*S*,*S*)-**13**

2-Formylpyrrole (*S*,*S*)-**8** (145 mg, 0.298 mmol) was dissolved in CH_2_Cl_2_ (4.5 mL), and a solution of *para*-toluenesulfonic acid monohydrate (113 mg, 0.596 mmol, 2.0 equiv) in MeOH (1.5 mL) was added. The resulting mixture was stirred for 4 h at rt while being monitored by TLC. After completion, the reaction mixture was neutralized with a saturated aqueous NaHCO_3_ solution (3 mL), and the aqueous layer was extracted with CH_2_Cl_2_ (2 × 10 mL). The combined organic solutions were washed with brine (15 mL), dried over anhydrous Na_2_SO_4_, and concentrated in vacuo. The resulting crude product was purified by flash column chromatography (EtOAc:petroleum ether, 3:1 → 4:1), yielding a pale-yellow oil (107 mg, 0.274 mmol for mole-fraction-weighted M = 391.21 g.mol^−1^, 92%). The isolated product contained 85% of (*S*,*S*)-**13** and 15% of (*S*,*S*)-**14**, as confirmed by HPLC and ^1^H NMR. This mixture was used in the next step.

#### 3.2.15. (–)-Hemerocallisamine I, (*S*,*S*)-**1**

Step **f**: The mixture of (*S*,*S*)-**13** and (*S*,*S*)-**14** (85:15) (96 mg, 0.245 mmol for mole-fraction-weighted M = 391.21 g.mol^−1^) was dissolved in dry MeOH (2.8 mL). MeONa (25w% solution in MeOH) (5.4 mg, 27 μL, 0.099 mmol, 0.4 equiv) was added to the solution at 0 °C, under an argon atmosphere. The reaction mixture was stirred at 0 °C. After 30 min, a saturated aqueous NH_4_Cl solution (3 mL) was added at 0 °C, and the resulting mixture was extracted with EtOAc (3 × 10 mL). The combined organic layers were washed with brine (10 mL), dried over anhydrous Na_2_SO_4_, and concentrated in vacuo*,* yielding crude (*S*,*S*)-**14** as a thick pale-yellow oil (96 mg, 0.229 mmol, 93%).

Step **g**: The crude methyl ester (*S*,*S*)-**14** (96.0 mg, 0.229 mmol) was dissolved in a mixture of CH_2_Cl_2_ (2.8 mL) and a phosphate buffer (NaH_2_PO_4_, Na_2_HPO_4_; pH 7, *c* 1M; 0.450 mL). Subsequently, 2,3-dichloro-5,6-dicyano-1,4-benzoquinone (156 mg, 0.688 mmol, 3.0 equiv) was added to the solution. The resulting mixture was stirred at rt while the reaction progress was monitored by HPLC and TLC. After 24 h, the reaction mixture was diluted with CH_2_Cl_2_ (5 mL), dried with Na_2_SO_4_, and filtered through a Celite pad. The pad was washed first with CH_2_Cl_2_ (10 mL), then with dioxane (3 × 20 mL). The combined filtrates were concentrated in vacuo and purified by flash column chromatography (CH_2_Cl_2_:MeOH, 13:1). (–)-Hemerocallisamine I (27 mg, 0.091 mmol, 40%, er > 99:1) was isolated after trituration from MeOH:Et_2_O as a white powder. Mp 171.2−172.8 °C; [α]^25^_D_ -34.5 (*c* 0.12, MeOH); ^1^H NMR (400 MHz, DMSO-*d_6_*): δ = 9.37 (s, 1H), 7.24 (s, 1H), 7.20–7.07 (m, 2H), 6.33 (d, *J* = 4.0 Hz, 1H), 5.70 (bd, *J* = 5.7 Hz, 1H), 5.26 (bs, 1H), 4.73 (bd, *J* = 13.2 Hz, 1H), 4.36 (d, *J* = 13.1 Hz, 1H), 3.56 (s, 3H), 3.19 (s, 3H), 3.09–3.00 (m, 1H), 2.41–2.25 (m, 2H); ^13^C NMR (100 MHz, DMSO-*d_6_*): δ = 178.89, 175.83, 169.78, 140.56, 131.96, 125.96, 111.06, 67.75, 64.82, 57.02, 55.20, 52.14, 36.57.

## 4. Conclusions

The synthesis of the pyrrolic alkaloid (–)-hemerocallisamine I, featuring crystallization-induced diastereomer transformation (CIDT) and the Maillard reaction as the key synthetic strategies, was achieved in 12 steps and a 1.6% overall yield. The sequence involved the preparation of (2*S*,4*S*)-4-hydroxyglutamic acid lactone in gram quantities, from an achiral substrate. In parallel, the first synthesis of (±)-hemerocallisamine I was described in 11 steps and a 5.9% overall yield. A detailed inspection of the Maillard reaction conditions revealed diketopiperazine **12** as the dominant side product, arising from a cannibalistic reaction of the amine **11**. This transformation might be responsible for the often-reported depletion of the starting amino-acid-derived amines in the Maillard-type condensations.

## Figures and Tables

**Table 1:** Optimization of the Maillard reaction step.

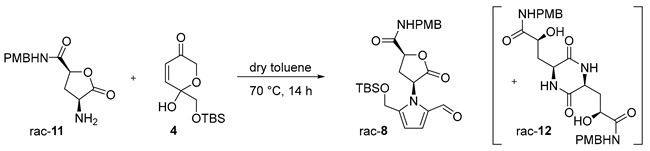		
Entry	Variation from the Optimized Conditions ^a,b^	Yield (%) ^c^
1	none	47 (43 ^d^)
2	90 °C	47
3	rt	36
4	MeCN, 60 °C	30
5	THF, 60 °C	22
6	THF/H_2_O 1:1, 55 °C	20
7	wet DCE, 55 °C	43
8	CH_2_Cl_2_, 35 °C	41
9	CH_2_Cl_2_, rt	33
10	+0.1 equiv rac-**11**, CH_2_Cl_2_, rt	37
11	AcOH (cat.), wet CH_2_Cl_2_, rt	32
12	TEA (1 equiv), CH_2_Cl_2_, rt	0

^a^ Optimized reaction conditions: rac-**11** (1.0 equiv), **4** (1.0 equiv), dry toluene (0.2 M), 70 °C, under argon, 14 h. ^b^ The solvents used were dry unless otherwise specified. ^c^ Yields based on ^1^H NMR with the internal standard are reported, unless otherwise noted. ^d^ Isolated yield. PMB = *p*-methoxybenzyl, TBS = *tert*-butyldimethylsilyl, DCE = 1,2-dichloroethane, PTSA = *p*-toluenesulfonic acid, TEA = triethylamine.

## Data Availability

Not applicable.

## Supplementary Materials

The following supporting information can be downloaded at: https://www.mdpi.com/1420-3049/28/5/2177/s1, Pages S2–S4: Synthesis and characterization data of compounds **6**, **4** and rac-**12**; Pages S5–S6: HPLC data for compounds (*S*,*S*)-**8** and (*S*,*S*)-**1**; Pages S7–S8: Optimization of the Maillard reaction; Page S9: X-ray analysis of compound (*S*,*S*)-**1**; Pages S10–S27: ^1^H and ^13^C NMR spectra [42,43,44,45,46].
