# Supplementary material for: Total Synthesis of Hemerocallisamine I Paved by Gram-Scale Synthesis of (2S,4S)-4-Hydroxyglutamic Acid Lactone
Source: Molecules. 2023 Feb 26;28(5):2177. doi: 10.3390/molecules28052177 (PMC10037412; doi:10.3390/molecules28052177)
Supplement: Supplementary file 1 [file molecules-28-02177-s001.zip › molecules-2242046-supplementary.pdf]

## SUPPORTING INFORMATION

# Total Synthesis of Hemerocallisamine I Paved by Gram Scale Synthesis of (2*S*,4*S*)-4-Hydroxyglutamic Acid Lactone


1. Materials and methods S2
2. Synthesis and characterization of compounds **6**, **4** and rac-**12** S2
3. HPLC data for compounds (*S*,*S*)-**8** and (*S*,*S*)-**1** S5
4. Optimization of the Maillard reaction S7
5. X-ray analysis of compound (*S*,*S*)-**1** S9
6. $^{1}$H and $^{13}$C NMR spectra S10
7. References S28

## 1. Materials and methods

Unless otherwise noted, all chemicals were purchased from commercial sources and used without further purifications. Column chromatography was carried out using silica 60 A, Davisil, purchased from Fisher Chemicals. Reactions were monitored by thin layer chromatography (TLC), using Macherey-Nagel's pre-coated TLC sheets POLYGRAM SIL G/UV254, which were visualized under UV light (254 nm) or by staining with aqueous basic potassium permanganate or cerium molybdate solutions as appropriate. HPLC analyses were performed on a Varian system using a Macherey-Nagel EC 250/4 Nucleodur Phenyl-Hexyl 5 µm, CHIRAL ART, Amylose-SA, 250 x 4.6 mm, 5 µm and Astec CHIROBIOTIC ®T, 250 x 4.6 mm, 5 µm column. All $^{1}H$ and $^{13}C$ NMR spectra were recorded using Bruker Avance NEO 400 MHz and/or Varian 400 MR spectrometers. Chemical shifts (δ) are given in parts per million (ppm). The $^{1}H$ NMR chemical shift scale is referenced to the TMS internal standard (δ = 0 ppm) or solvent residual peak (δ = 2.50 ppm for DMSO-$d_6$ and δ = 7.26 ppm for $CDCl_3$). The $^{13}C$ NMR chemical shift scale is referenced to the solvent residual peak (δ = 39.52 ppm for DMSO-$d_6$ and δ = 77.16 ppm for $CDCl_3$). Coupling constants (*J*) are given in hertz (Hz). The multiplicity of $^{1}H$ NMR signals is reported as follows: s = singlet, d = doublet, t = triplet, q = quartet, m = multiplet, bs = broad singlet, "t" for dd with two identical or similar coupling constants, "dt" or "td" for ddd with two identical or similar coupling constants and "q" for ddd with three identical or similar coupling constants. High-resolution mass spectra were measured using Thermo Scientific mass spectrometer with Orbitrap analyzer and HESI ionization.

## 2. Synthesis and characterization of compounds

### (*E*)-4-(4-methoxyphenyl)-4-oxobut-2-enoic acid (6)

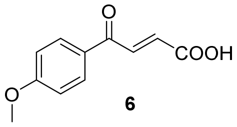


Anisole (20.0 g, 0.185 mol) was dissolved in $CH_2Cl_2$ (111 ml), and then maleic anhydride (27.2 g, 0.277 mol, 1.5 equiv) and $AlCl_3$ (37.0 g, 0.277 mol, 1.5 equiv) were added. The reaction mixture was stirred for 20 h at rt. After completion, the reaction mixture was quenched with $H_2O$ (100 ml) and $CH_2Cl_2$ was evaporated under reduced pressure. The residue was diluted with $H_2O$ (150 ml) and extracted with EtOAc (3 x 150 ml). The collected organic layers were washed with saturated brine (200 ml), dried over anhydrous $Na_2SO_4$, and concentrated *in vacuo,* yielding a crude product that was crystallized from EtOAc (120 ml). Acid **6** (21.0 g, 0.102 mol, 55%) was isolated as a yellow solid. The $^{1}H$ NMR spectrum of **6** was consistent with the published data.[1]

### 6-(((*tert*-Butyldimethylsilyl)oxy)methyl)-6-hydroxy-2H-pyran-3(6H)-one (4)

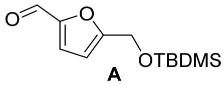


Step 1: Imidazole (5.94 g, 87.2 mmol, 1.1 equiv) and 5-hydroxymethylfurfural (10.0 g, 79.3 mmol) were stirred in $CH_2Cl_2$ (198 ml) for 15 minutes at rt. TBDMSCl (12.5 g, 83.3 mmol, 1.05 equiv) was added afterwards and the reaction mixture was stirred at rt, under argon, while being monitored by TLC. After 16 h $H_2O$ (200 ml) was added, the organic phase was separated, and the aqueous phase was extracted with $CH_2Cl_2$ (2 x 150 ml). The combined organic solutions were dried over anhydrous $Na_2SO_4$ and concentrated *in vacuo*, providing carbaldehyde **A** (18.3 g, 76.1 mmol, 96%) as an orange oil.

Rf 0.77 (EtOAc:Hex 1:5)

$^1H$ NMR (300 MHz, $CDCl_3$) : δ 9.59 (s, 1H), 7.20 (d, *J* = 3.6 Hz, 1H), 6.47 (dt, *J* = 3.5, 0.8 Hz, 1H), 4.73 (d, *J* = 0.5 Hz, 2H), 0.92 (s, 9H), 0.10 (s, 6H).

$^1H$-NMR spectrum of **A** was consistent with the published data.[2]

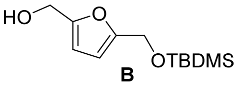


**B**

Step 2: $NaBH_4$ (5.76 g, 152 mmol, 2.0 equiv) was added portionwise to a solution of carbaldehyde **A** (18.3 g, 76.1 mmol) in MeOH (400 ml) at 0 °C, and the reaction mixture was stirred at this temperature for 45 min. Then $H_2O$ (200 ml) was added and MeOH was removed *in vacuo*. The residue was diluted with $H_2O$ (200 ml) and extracted with EtOAc (250 and 2 x 200ml). The combined organic layers were dried over anhydrous $Na_2SO_4$ and concentrated under reduced pressure. The crude product was purified by flash column chromatography (EtOAc:Hex, 1:1 to 3:1) to give **B** (15.0 g, 61.9 mmol, 81%) as a pale-yellow oil.

Rf 0.44 (EtOAc:Hex 1:4)

$^1H$ NMR (300 MHz, $CDCl_3$) : δ = 6.24 – 6.20 (m, 1H), 6.17 (d, *J* = 3.1 Hz, 1H), 4.62 (s, 2H), 4.59 (s, 2H), 1.78 (s, 1H), 0.90 (s, 9H), 0.08 (s, 6H).

$^1H$-NMR spectrum of **B** was consistent with the published data.[3]

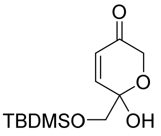


**4**

Step 3: *m*-CPBA (18.0 g, 80.5 mmol 1.3 equiv) was added to a solution of **B** (15 g, 61.8 mmol) in $CH_2Cl_2$ (495 ml) at 0 °C and the mixture was allowed to stir at this temperature for 0.5 h. Afterwards the mixture was let to reach rt and was stirred for additional 4 h while being monitored by TLC. The reaction was quenched with a saturated aqueous $Na_2SO_3$ solution (150 ml), followed by neutralisation to pH 7–8 with 1M aqueous NaOH. The mixture was extracted with $CH_2Cl_2$ (2 x 80 mL), the combined organic extracts were washed with brine and dried over anhydrous $Na_2SO_4$ to give dihydropyranone **4** (13.4 g, 51.9 mmol, 83%) as an off-white solid.

Mp 83 – 84 °C; Rf 0.55 (EtOAc:Hex 1:5)

$^1H$ NMR (400 MHz, $CDCl_3$): δ = 6.79 (d, *J* = 10.4 Hz, 1H), 6.15 (d, *J* = 10.4 Hz, 1H), 4.59 (d, *J* = 16.9 Hz, 1H), 4.14 (d, *J* = 17.0 Hz, 1H), 3.77 (d, *J* = 10.2 Hz, 1H), 3.68 (d, *J* = 10.2 Hz, 1H), 0.93 (s, 9H), 0.12 (s, 6H).

$^1H$-NMR spectrum of **4** was consistent with the published data.[3]

## Isolation of the diketopiperazine byproduct rac-12

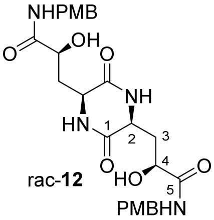


rac-**12**

Amine rac-**11** (153.0 mg, 0.579 mmol) was dissolved in dry $CH_2Cl_2$ (2.9 ml) and dihydropyranone **4** (149.6 mg, 0.579 mmol, 1.0 equiv) was added. The reaction mixture was stirred at rt under argon. After 14 h, the reaction mixture was concentrated *in vacuo* and the crude product was treated with a mixture of MeOH and $Et_2O$ (1:1, 2 ml). The precipitate was filtered off and washed with a small amount of MeOH and $Et_2O$, yielding rac-**12** as a white solid (13.0 mg, 0.025 mmol, 9% yield with respect to the reaction stoichiometry rac-**11**/rac-**12** 2:1).

$^1H$ NMR (400 MHz, DMSO-$d_6$): δ = 8.32 – 8.25 (m, 2H, 2xPMB-NH), 8.23 (m, 2H, 2xNH), 7.18 (d, *J* = 8.7 Hz, 4H, 2xPMB-Ar), 6.86 (d, *J* = 8.5 Hz, 4H, 2xPMB-Ar), 5.84 (d, *J* = 5.5 Hz, 1H, OH), 5.79 (d, *J* = 5.5 Hz, 1H, OH),

4.22 (d, $J$ = 6.2 Hz, 4H, 2xPMB-CH2), 4.19 – 4.12 (m, 2H, 2xH-4), 4.01 – 3.93 (m, 2H, 2xH-2), 3.72 (s, 6H, 2xPMB-Me), 2.05 – 1.83 (m, 4H, 2xH-3)
$^{1}$H NMR was assigned on the basis of 2D NMR, the numbering is specified in the figure.

$^{13}$C NMR (101 MHz, DMSO-$d_6$): δ = 173.28 (C-5), 168.51 (C-1), 158.16 (PMB-Ar), 131.54 (PMB-Ar), 128.53 (PMB-Ar), 113.63 (PMB-Ar), 68.00 (C-4), 55.03 (PMB-Me), 51.58 (C-2), 41.23 (PMB-CH2), 37.56 (C-3).

HRMS–HESI (m/z): calcd for $C_{26}H_{32}N_4O_8Na$ $[M+Na]^+$, 551.21124, found 551.21137.

## 3. HPLC data for compounds (*S*,*S*)-8 and (*S*,*S*)-1

**HPLC conditions:** CHIRAL ART, Amylose-SA, 250 x 4.6 mm, 5 µm.
Mobile Phase: Hexane:Propan-2-ol 6:1, λ = 254 nm, flow 1 ml/min
er > 99:1

20. 1. 2023 9:01 Chromatogram C:\Clarity\DataFiles\WORK3\Data\LP371 rac hex-iPrOH 6-1 1 mL_min, 254 nm.prm Page 2 of 2

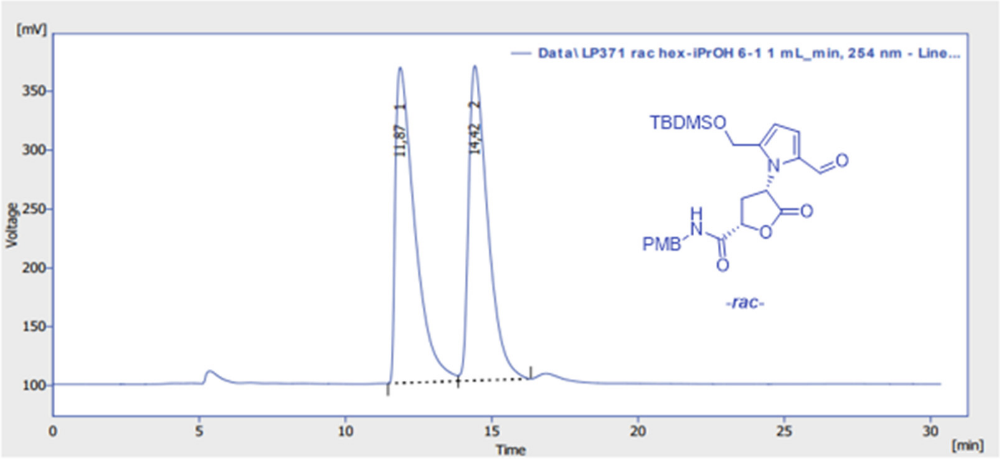


Result Table (Uncal - Data\LP371 rac hex-iPrOH 6-1 1 mL_min, 254 nm - Linear UV)

| | Reten. Time [min] | Area [mV.s] | Height [mV] | Area [%] | Height [%] | W05 [min] | Compound Name |
|---|---|---|---|---|---|---|---|
| 1 | 11,870 | 12463,255 | 268,299 | 50,6 | 50,1 | 0,69 | |
| 2 | 14,422 | 12155,452 | 267,654 | 49,4 | 49,9 | 0,69 | |
| | Total | 24618,707 | 535,953 | 100,0 | 100,0 | | |

20. 1. 2023 9:01 Chromatogram C:\Clarity\DataFiles\WORK3\Data\LP398 ee purity hex-iPrOH 6-1 1 mL_min, 254 nm.prm Page 2 of 2

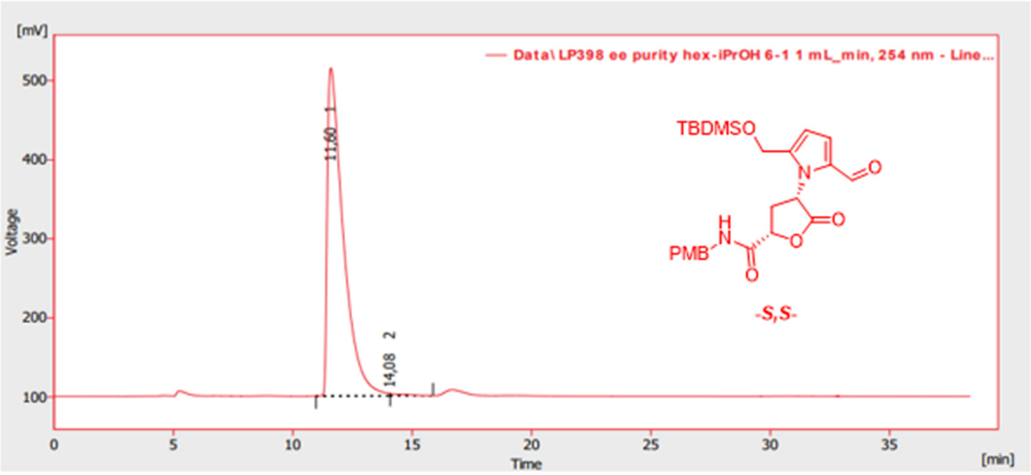


Result Table (Uncal - Data\LP398 ee purity hex-iPrOH 6-1 1 mL_min, 254 nm - Linear UV)

| | Reten. Time [min] | Area [mV.s] | Height [mV] | Area [%] | Height [%] | W05 [min] | Compound Name |
|---|---|---|---|---|---|---|---|
| 1 | 11,595 | 19350,645 | 414,591 | 99,3 | 99,3 | 0,68 | |
| 2 | 14,082 | 129,388 | 2,818 | 0,7 | 0,7 | 0,84 | |
| | Total | 19480,033 | 417,409 | 100,0 | 100,0 | | |

**HPLC conditions:** Astec CHIROBIOTIC ®T, 250 x 4.6 mm, 5 µm.
Mobile Phase: Methanol:Water 2:98, λ = 254 nm, flow 1 ml/min
er > 99:1

20. 1. 2023 8:55 Chromatogram C:\Clarity\DataFiles\WORK3\Data\LP hemerocallisamine I rac MeOH-H2O 2-98 17-1-22, 1 mL_min, 254 nm.prm Page 2 of 2

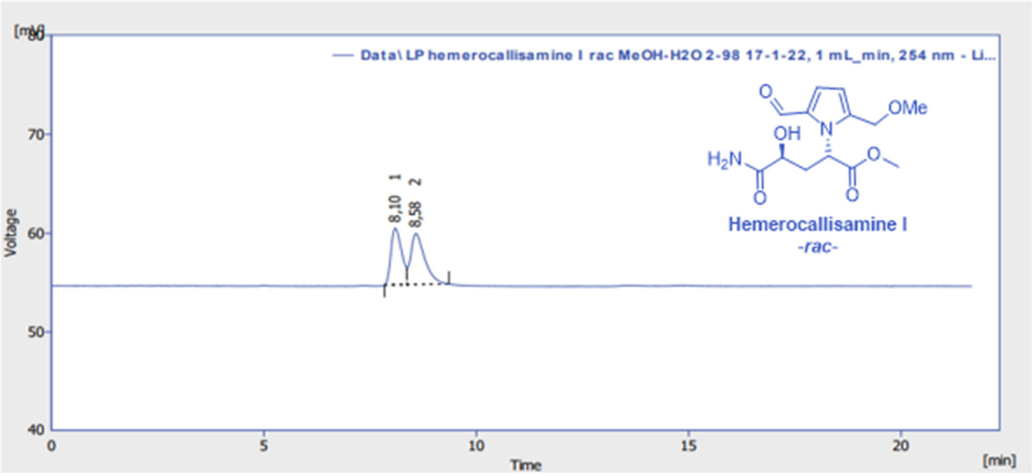


*Result Table (Uncal - Data\LP hemerocallisamine I rac MeOH-H2O 2-98 17-1-22, 1 mL_min, 254 nm - Linear UV)*

| | Reten. Time [min] | Area [mV.s] | Height [mV] | Area [%] | Height [%] | W05 [min] | Compound Name |
|---|---|---|---|---|---|---|---|
| 1 | 8,095 | 99,625 | 5,712 | 45,5 | 52,6 | 0,29 | |
| 2 | 8,578 | 119,320 | 5,149 | 54,5 | 47,4 | 0,35 | |
| | Total | 218,945 | 10,861 | 100,0 | 100,0 | | |

20. 1. 2023 8:5Chromatogram C:\Clarity\DataFiles\WORK3\Data\LP hemerocallisamine I enantiomer MeOH-H2O 2-98 17-1-22, 1 mL_min, 254 nm.prm Page 2 of 2

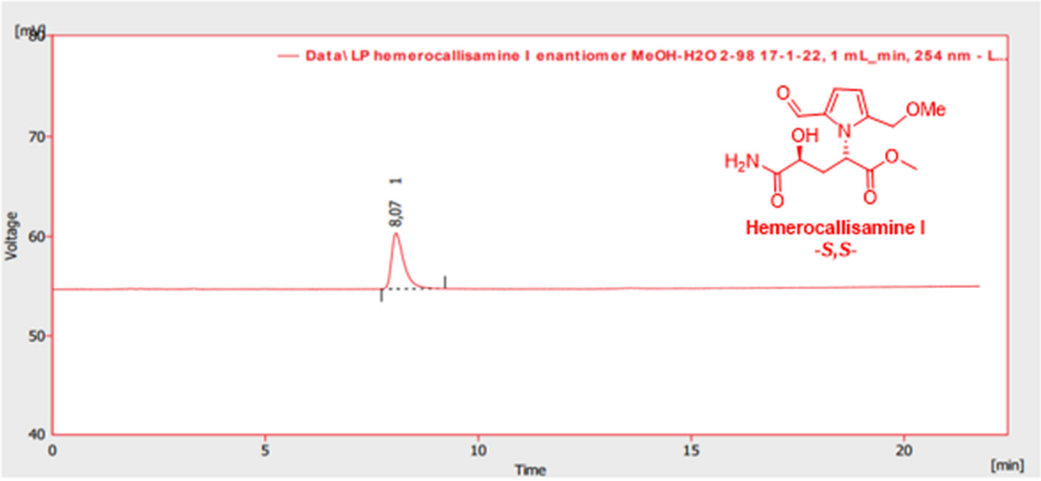


*Result Table (Uncal - Data\LP hemerocallisamine I enantiomer MeOH-H2O 2-98 17-1-22, 1 mL_min, 254 nm - Linear UV)*

| | Reten. Time [min] | Area [mV.s] | Height [mV] | Area [%] | Height [%] | W05 [min] | Compound Name |
|---|---|---|---|---|---|---|---|
| 1 | 8,070 | 114,480 | 5,611 | 100,0 | 100,0 | 0,30 | |
| | Total | 114,480 | 5,611 | 100,0 | 100,0 | | |

## 4. Optimization of the Maillard reaction

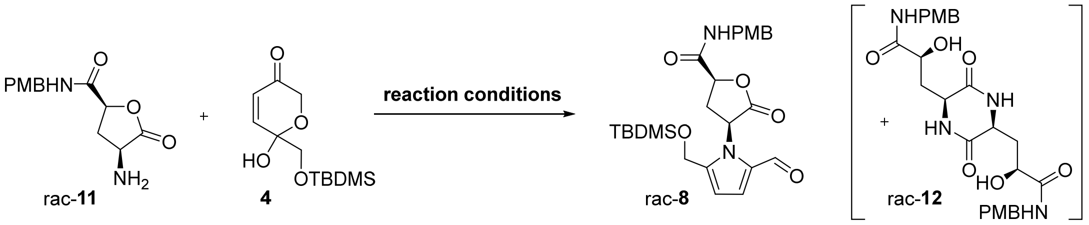


**General procedure for $^1$H-NMR experiment:**

Reagents rac-**11** (9.0 mg, 0.034 mmol) and **4** (8.8 mg, 0.034 mmol, 1.0 equiv) were dissolved in the specified solvents (see the Table). The resulting reaction mixtures were stirred under the specified conditions for 14 h. After completion, the solvent was evaporated *in vacuo.* The reactions yields were determined by $^1$H-NMR with the internal standard 1,1,2-trichloroethane (4.5 mg, 0.034 mmol, 3 µl, 1.0 equiv), in $CDCl_3$. In selected experiments (A, Q, R, Y, Z), yields of the by-product rac-**12** were determined by $^1$H-NMR in DMSO-$d_6$, with 1,1,2-trichloroethane as the internal standard.

| Experiment | A equiv | B equiv | Reaction conditions | P H-NMR – Yield ($CDCl_3$) | BP H-NMR – Yield*(DMSO-$d_6$) |
|---|---|---|---|---|---|
| **A** | 1 | 1 | dry $CH_2Cl_2$ (0.2M), Ar, rt, 14 h | *33%* | *16%* |
| **B** | 1.1 | 1 | dry $CH_2Cl_2$ (0.2M), Ar, rt, 14 h | *37%* | - |
| **C** | 1 | 1 | dry $CH_2Cl_2$ (0.2M), Ar, 35 °C, 14 h | *41%* | - |
| **D** | 1 | 1 | $CH_2Cl_2$ (0.2M), rt, 14 h | *36%* | - |
| **E** | 1 | 1 | TEA (1 equiv), dry $CH_2Cl_2$ (0.2M), Ar, rt, 14 h | *0%* | - |
| **F** | 1 | 1 | dry $CH_2Cl_2$ (0.2M), MS (3A), Ar, rt, 14 h | *36%* | - |
| **G** | 1 | 1 | dry $CH_2Cl_2$ (0.2M), $Na_2SO_4$, Ar, rt, 14 h | *35%* | - |
| **H** | 1 | 1 | DCE (0.2M), Ar, 55 °C, 14 h | *43%* | - |
| **I** | 1 | 1 | THF/$H_2O$ (0.2M), 55 °C, 14 h | *20%* | - |
| **J** | 1 | 1 | $CH_2Cl_2$ (0.2M), AcOH (cat.), rt, 14 h | *32%* | - |
| **K** | 1 | 1 | THF/$H_2O$ (0.2M), rt, 14 h | *21%* | - |
| **L** | 1 | 1 | THF/$H_2O$ (0.2M), AcOH (cat.), rt, 14 h | *19%* | - |
| **M** | 1 | 1 | dry MeCN (0.2 M), Ar, rt, 14 h | *23%* | - |
| **N** | 1 | 1 | dry MeCN (0.2 M), Ar, 60 °C, 14 h | *30%* | - |
| **O** | 1 | 1 | dry THF (0.2 M), Ar, rt, 14 h | *17%* | - |
| **P** | 1 | 1 | dry THF (0.2 M), Ar, 60 °C, 14 h | *22%* | - |
| **Q** | 1 | 1 | dry toluene (0.2M), Ar, rt, 14 h | *36%* | *25%* |
| **R** | 1 | 1 | dry toluene (0.2M), Ar, 70 °C, 14 h | *47%* | *23%* |
| **S** | 1 | 1 | dry toluene (0.2M), Ar, 90 °C, 14 h | *47%* | - |
| **T** | 1 | 1 | dry toluene (0.2M), Ar, 105 °C, 14 h | *37%* | - |
| **U** | 1 | 1 | DCE (0.2M), pTsOH (0.88 eq), Ar, rt, 14 h | *37%* | - |
| **V** | 1 | 1 | DCE (0.2M), pTsOH (0.75 eq), 55°C, 14 h | *23%* | - |
| **X** | 1 (gradual addition over 30 min) | 1 | dry $CH_2Cl_2$ (0.08M), Ar, 35°C, 14 h | *42%* | - |
| **Y** | 1 | 1 | dry $CH_2Cl_2$ (0.02 M), rt, 14 h | *30%* | *35%* |
| **Z** | 1 | 2 | dry $CH_2Cl_2$ (0.02 M), rt, 14 h | *37%* | *30%* |

* yields with respect to the reaction stoichiometry rac-**11**/rac-**12** 2:1

## 5. X-ray analysis of compounds (*S*,*S*)-1

Single-crystal diffraction data for (*S*,*S*)-**1** were collected on a Bruker D8 VENTURE Kappa Duo diffractometer equipped with a PHOTON III detector and two IµS microfocus sealed tubes (Cu, Mo). Data were collected at 120K using monochromated CuKα (λ= 1.54178 Å) primary radiation. Data reduction was carried out using the diffractometer software. The phase problem was solved by intrinsic phasing (SHELXT)[4] and the structural model was refined by full-matrix least-squares against $F^2$ (SHELXL).[5] Non-hydrogen atoms were refined anisotropically and with no constraints imposed. Hydrogen atoms were refined isotropically. The two hydrogen atoms attached to N1 were refined freely, while all other hydrogen atoms were put into idealized positions and were refined using the riding model.

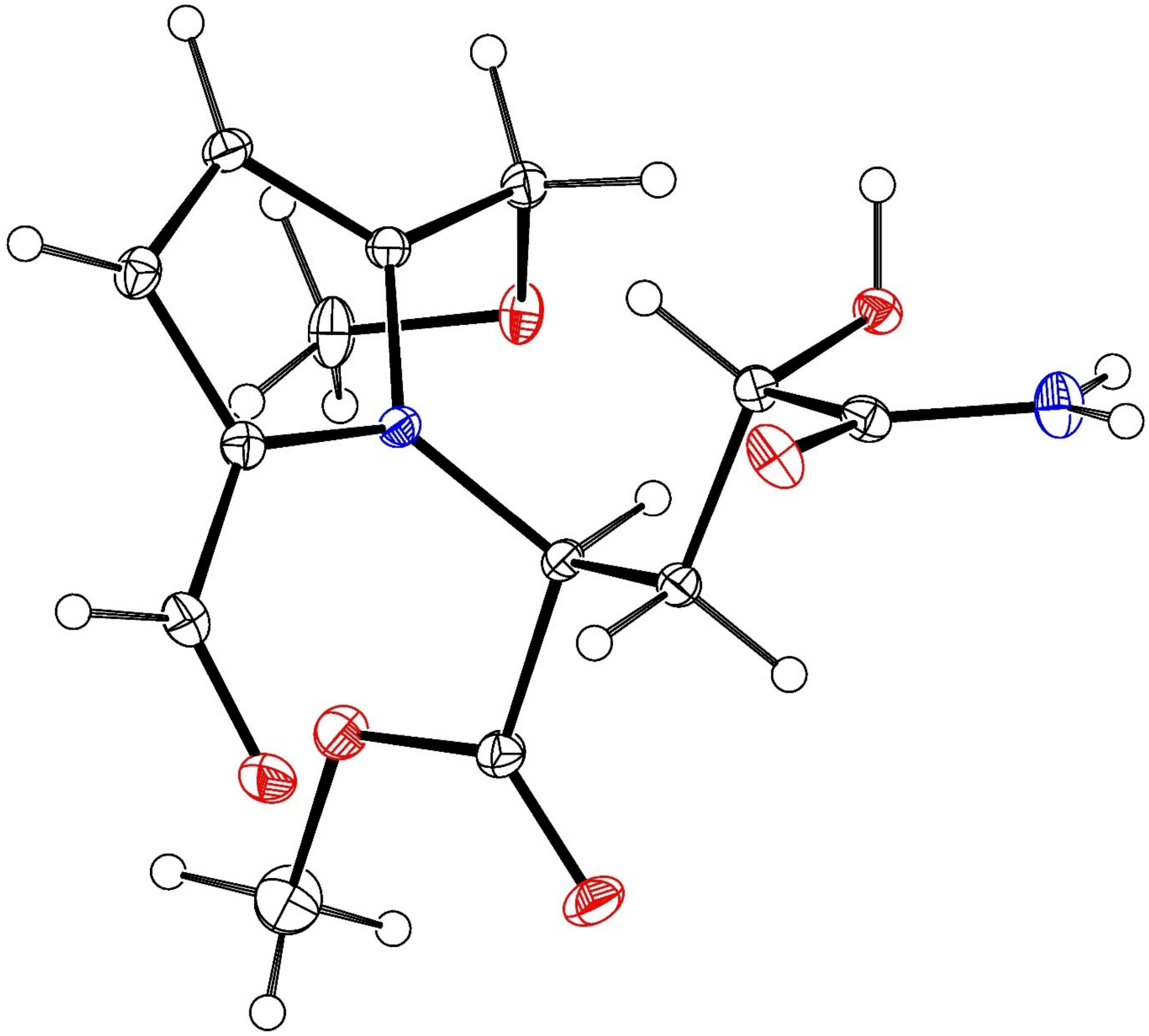

The molecular structure of (*S*,*S*)-**1** in solid state. Non-hydrogen atoms are displayed by thermal ellipsoids on 30% probability level.

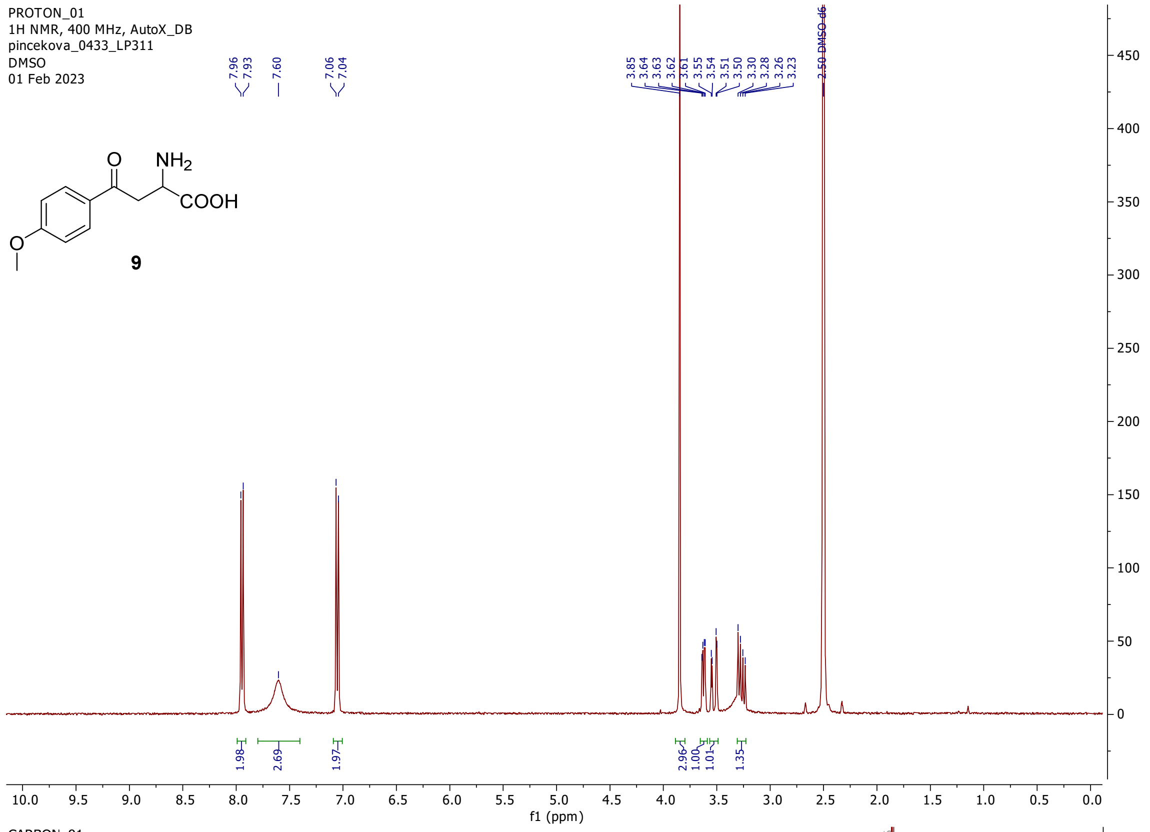
PROTON_01
1H NMR, 400 MHz, AutoX_DB
pincekova_0433_LP311
DMSO
01 Feb 2023
7.96
7.93
7.60
7.06
7.04
3.85
3.64
3.63
3.62
3.61
3.55
3.54
3.51
3.50
3.30
3.28
3.26
3.23
2.50 DMSO-d6
O
NH2
COOH
9
1.98
2.69
1.97
2.96
1.00
1.01
1.35
f1 (ppm)

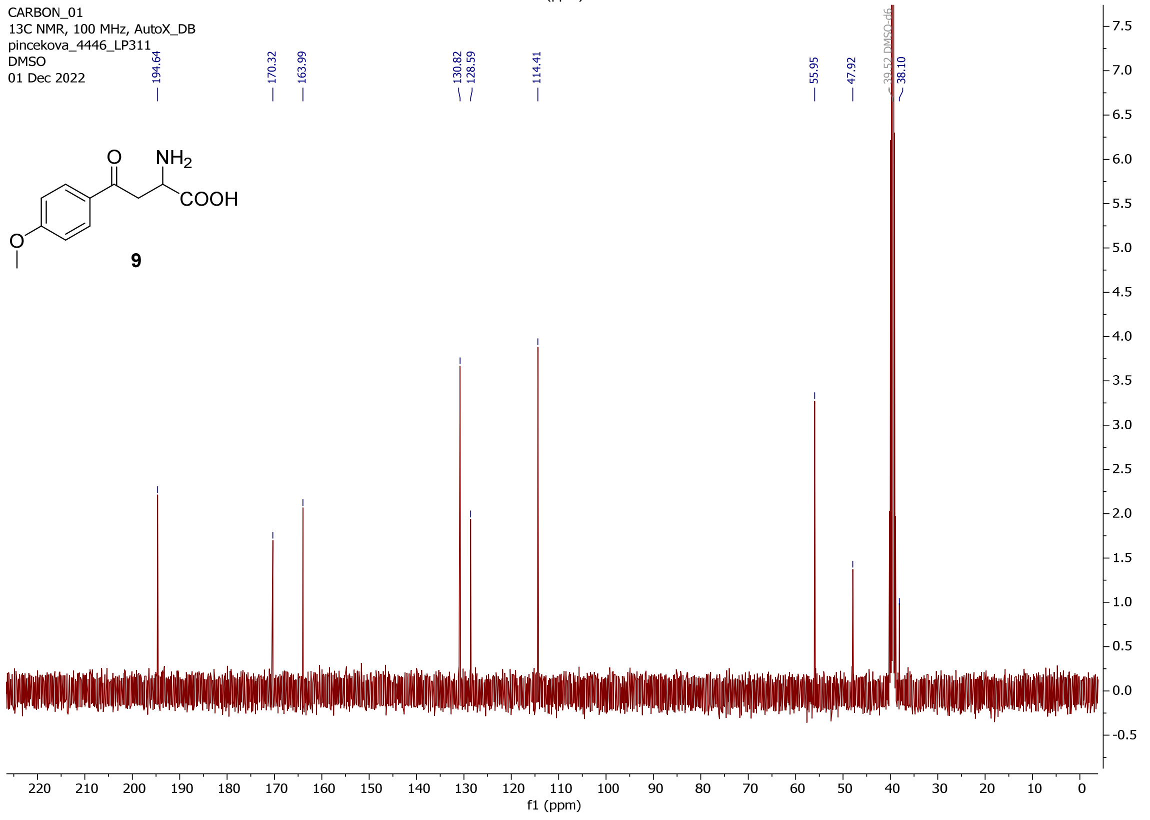
CARBON_01
13C NMR, 100 MHz, AutoX_DB
pincekova_4446_LP311
DMSO
01 Dec 2022
194.64
170.32
163.99
130.82
128.59
114.41
55.95
47.92
39.52 DMSO-d6
38.10
O
NH2
COOH
9
f1 (ppm)

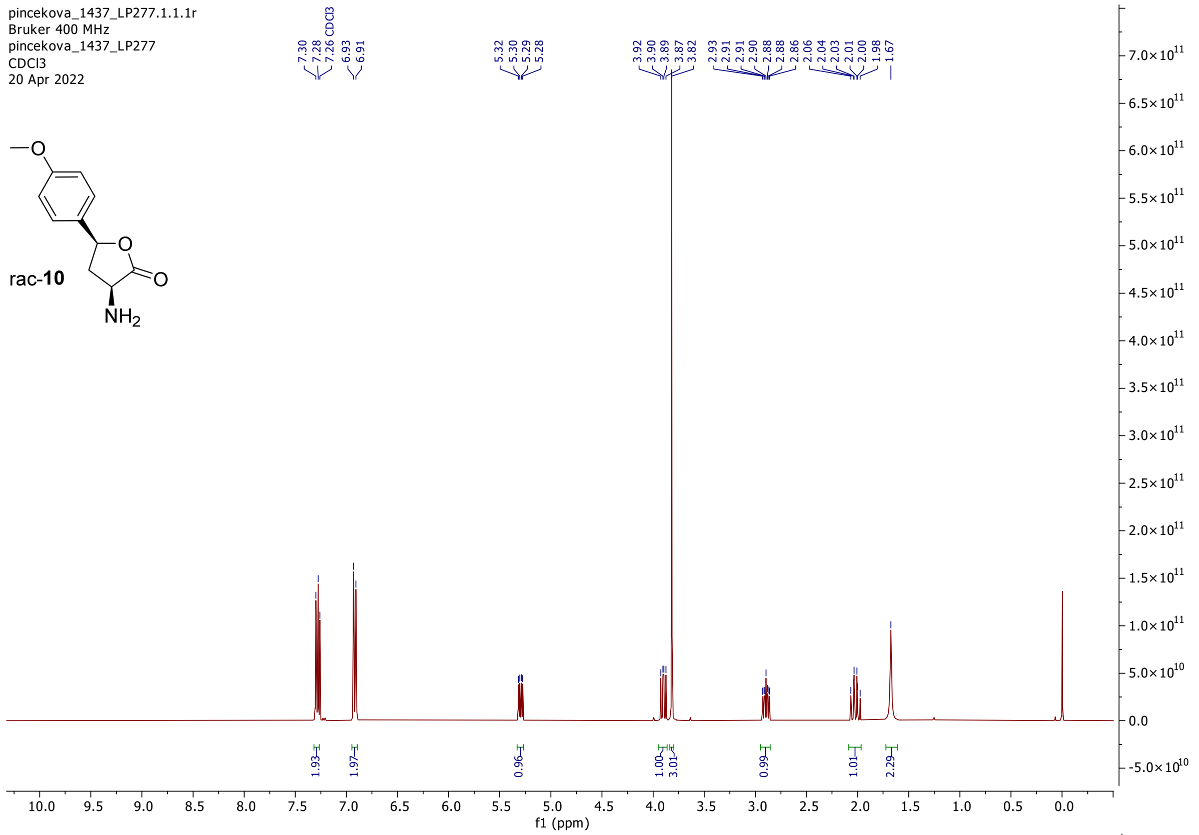
pincekova_1437_LP277.1.1.1r
Bruker 400 MHz
pincekova_1437_LP277
CDCl3
20 Apr 2022
rac-10
7.30
7.28
7.26 CDCl3
6.93
6.91
5.32
5.30
5.29
5.28
3.92
3.90
3.89
3.87
3.82
2.93
2.91
2.91
2.90
2.88
2.88
2.86
2.06
2.04
2.03
2.01
2.00
1.98
1.67
1.93
1.97
0.96
1.00
3.01
0.99
1.01
2.29
f1 (ppm)

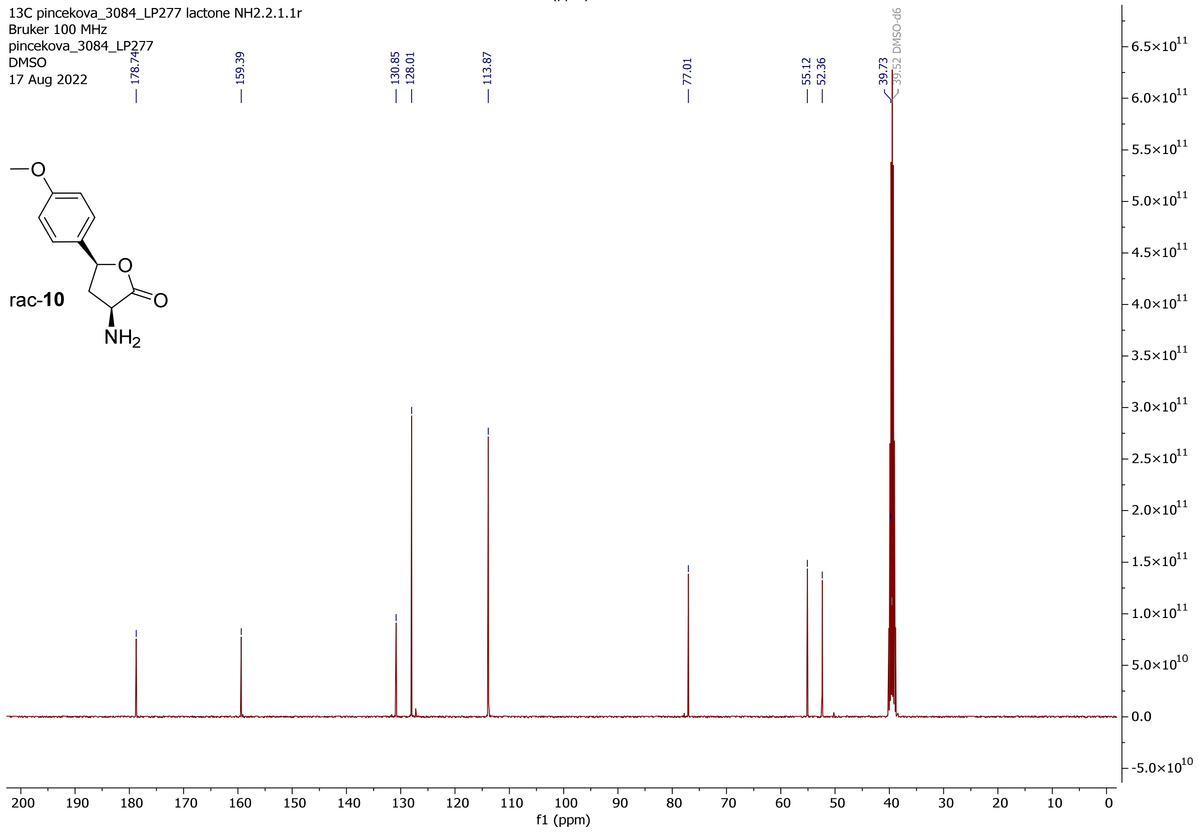
13C pincekova_3084_LP277 lactone NH2.2.1.1r
Bruker 100 MHz
pincekova_3084_LP277
DMSO
17 Aug 2022
rac-10
178.74
159.39
130.85
128.01
113.87
77.01
55.12
52.36
39.73
39.52 DMSO-d6
f1 (ppm)

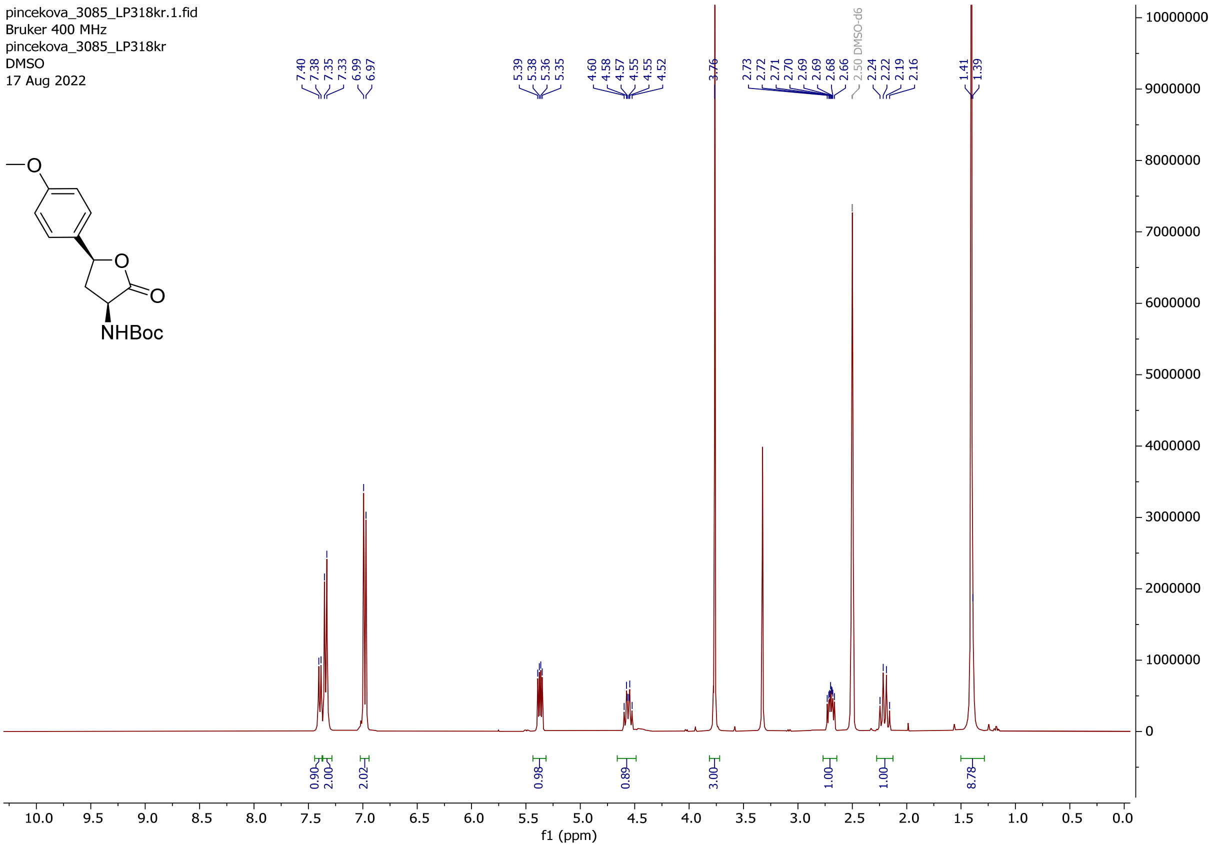
pincekova_3085_LP318kr.1.fid
Bruker 400 MHz
pincekova_3085_LP318kr
DMSO
17 Aug 2022
7.40 7.38 7.35 7.33 6.99 6.97
5.39 5.38 5.36 5.35
4.60 4.58 4.57 4.55 4.55 4.52
3.76
2.73 2.72 2.71 2.70 2.69 2.69 2.68 2.66
2.50 DMSO-d6
2.24 2.22 2.19 2.16
1.41 1.39
NHBoc
O
0.90 2.00 2.02 0.98 0.89 3.00 1.00 1.00 8.78
f1 (ppm)

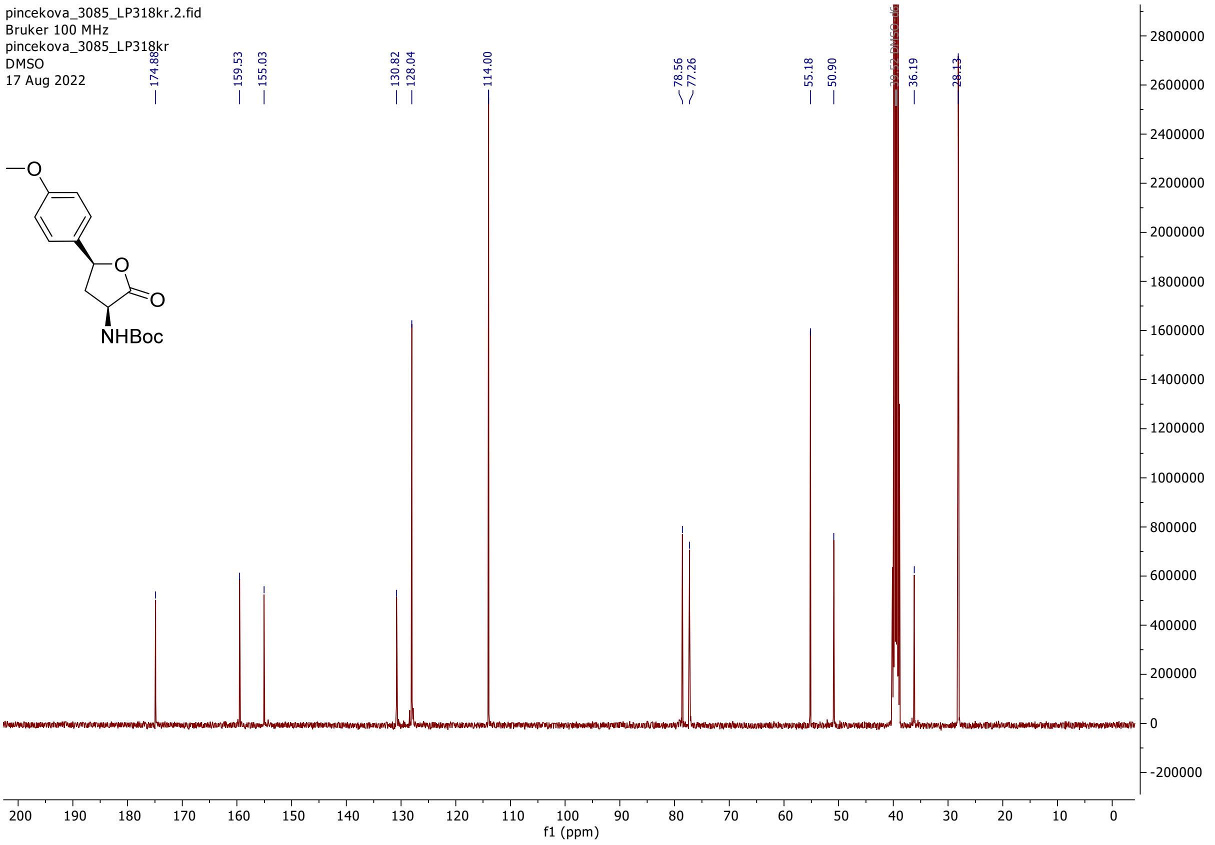
pincekova_3085_LP318kr.2.fid
Bruker 100 MHz
pincekova_3085_LP318kr
DMSO
17 Aug 2022
174.88
159.53
155.03
130.82
128.04
114.00
78.56
77.26
55.18
50.90
36.19
28.13
NHBoc
O
f1 (ppm)

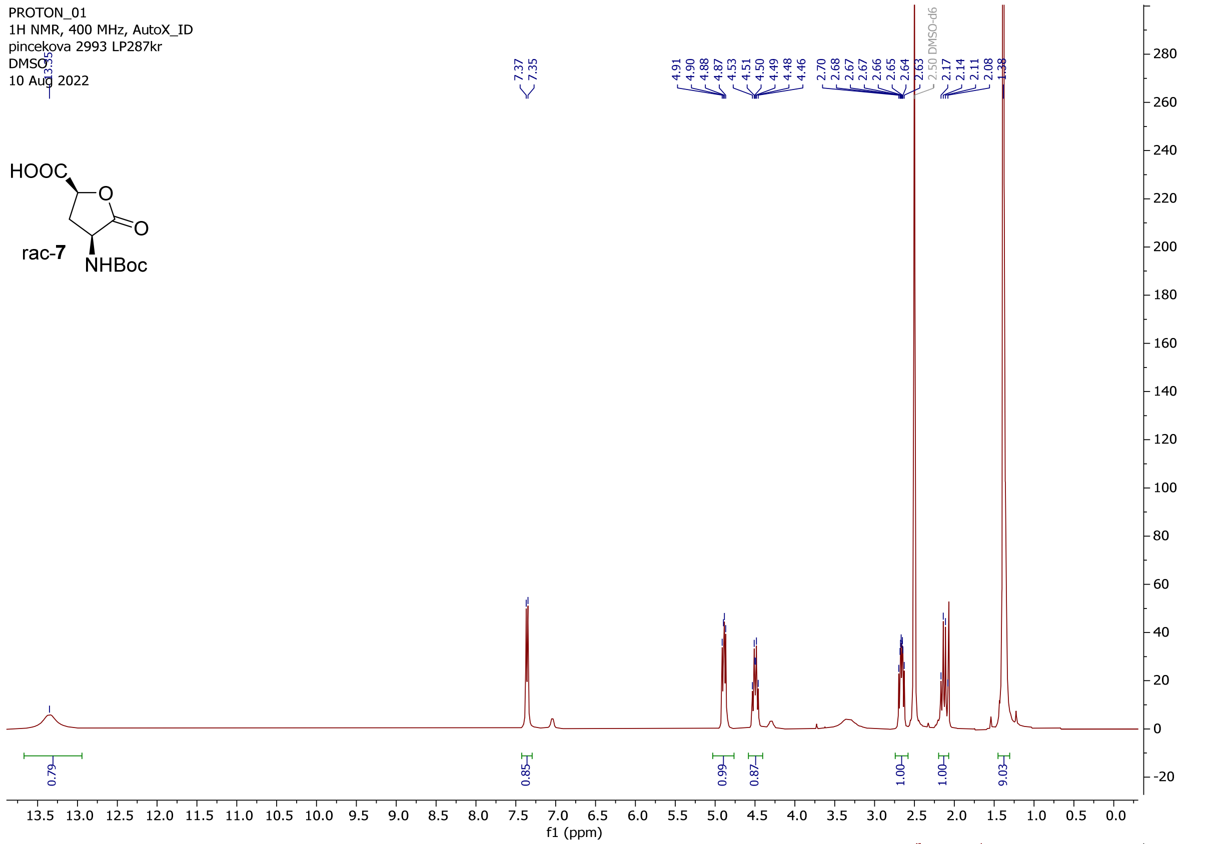
PROTON_01
1H NMR, 400 MHz, AutoX_ID
pincekova 2993 LP287kr
DMSO
10 Aug 2022
HOOC
O
O
rac-7
NHBoc
f1 (ppm)

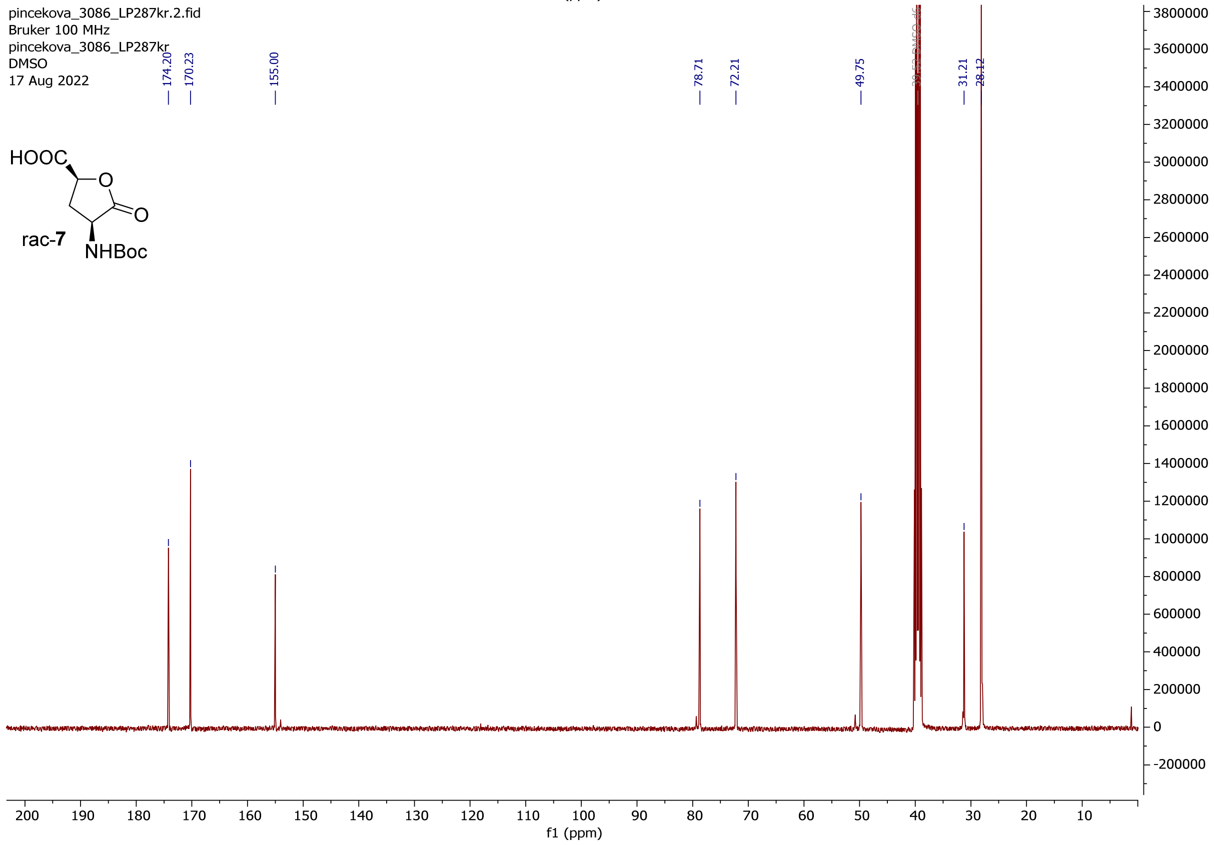
pincekova_3086_LP287kr.2.fid
Bruker 100 MHz
pincekova_3086_LP287kr
DMSO
17 Aug 2022
174.20
170.23
155.00
78.71
72.21
49.75
31.21
28.12
HOOC
O
O
rac-7
NHBoc
f1 (ppm)

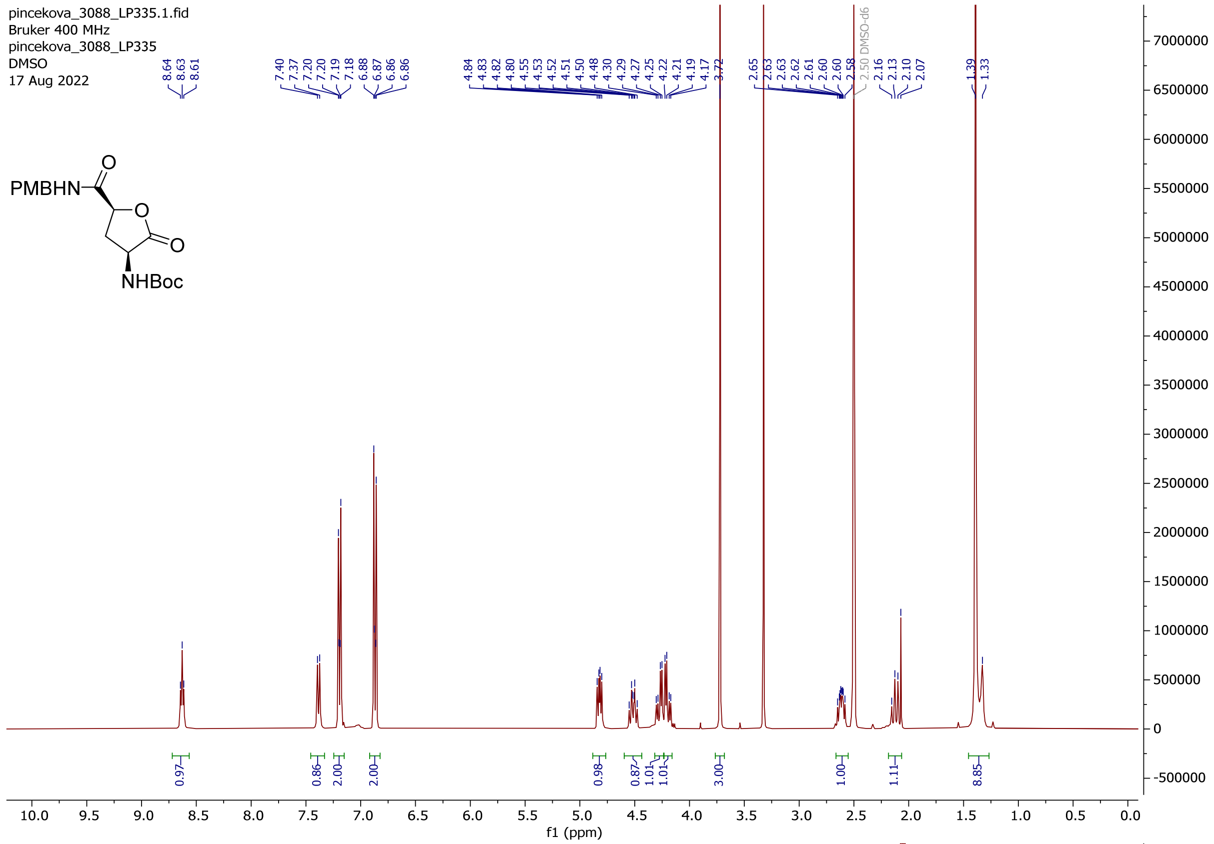
pincekova_3088_LP335.1.fid
Bruker 400 MHz
pincekova_3088_LP335
DMSO
17 Aug 2022
8.64 8.63 8.61
7.40 7.37 7.20 7.20 7.19 7.18 6.88 6.87 6.86 6.86
4.84 4.83 4.82 4.80 4.55 4.53 4.52 4.51 4.50 4.48 4.30 4.29 4.27 4.25 4.22 4.21 4.19 4.17 3.72
2.65 2.63 2.63 2.62 2.61 2.60 2.60 2.58
2.50 DMSO-d6
2.16 2.13 2.10 2.07
1.39 1.33
PMBHN
O
O
O
NHBoc
0.97 0.86 2.00 2.00 0.98 0.87 1.01 1.01 3.00 1.00 1.11 8.85
f1 (ppm)

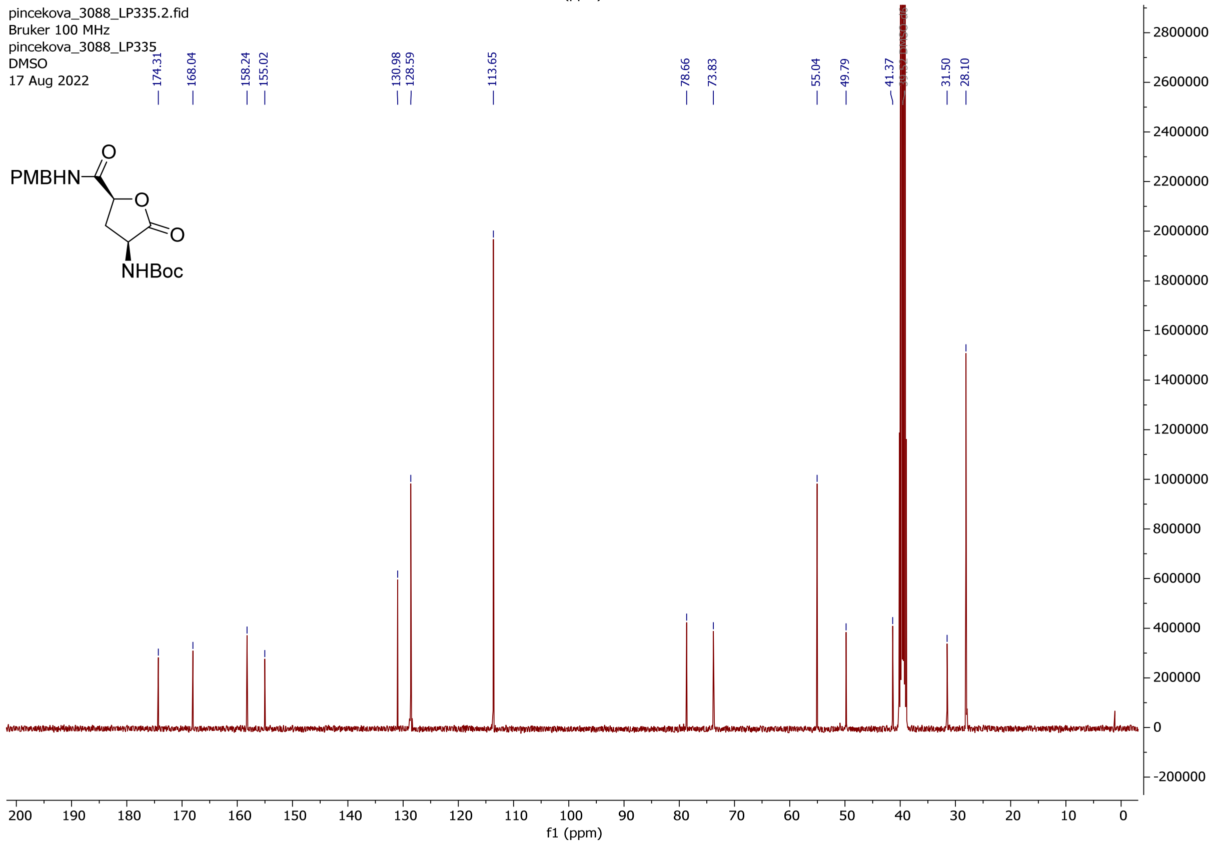
pincekova_3088_LP335.2.fid
Bruker 100 MHz
pincekova_3088_LP335
DMSO
17 Aug 2022
174.31 168.04 158.24 155.02 130.98 128.59 113.65 78.66 73.83 55.04 49.79 41.37 31.50 28.10
PMBHN
O
O
O
NHBoc
f1 (ppm)

PROTON_01
1H NMR, 400 MHz, AutoX_DB
pincekova_4744_LP422-salt
DMSO
20 Dec 2022

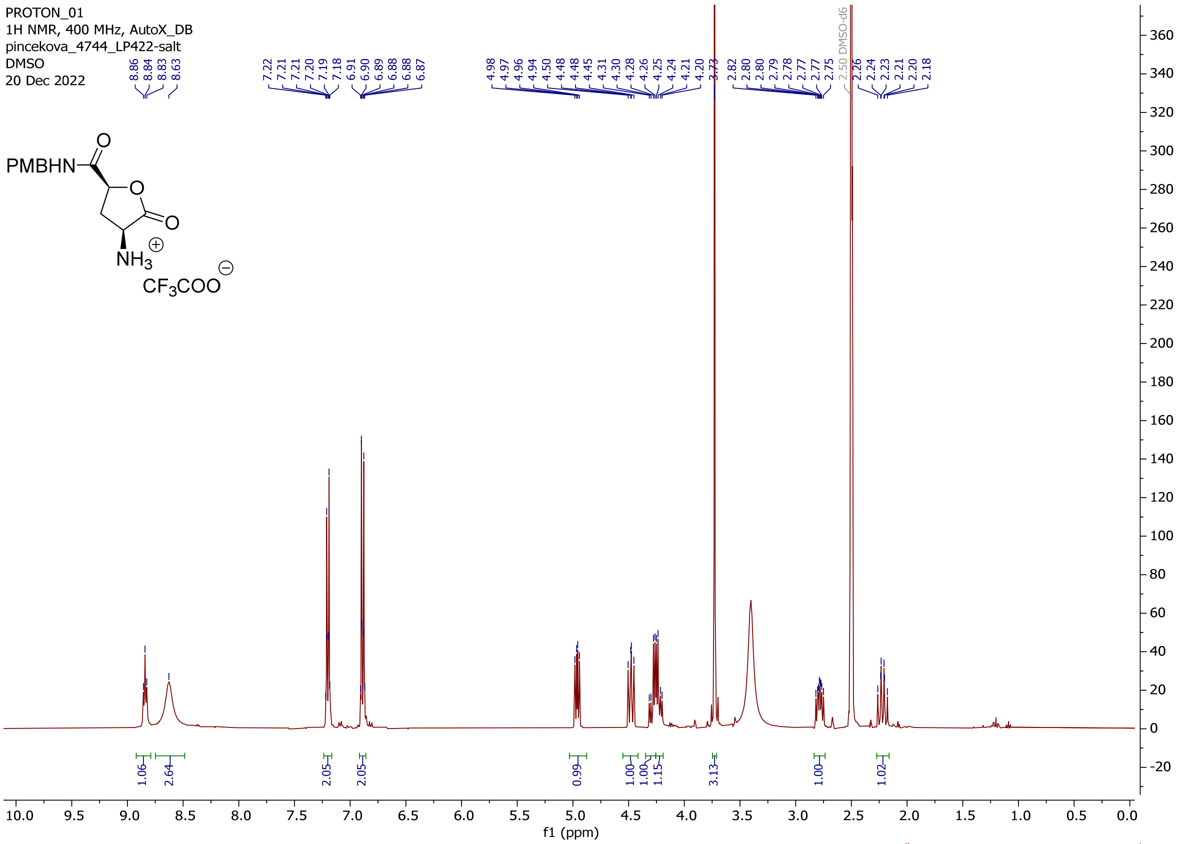


CARBON_01
13C NMR, 100 MHz, AutoX_DB
pincekova_4692_LP421-salt
DMSO
15 Dec 2022

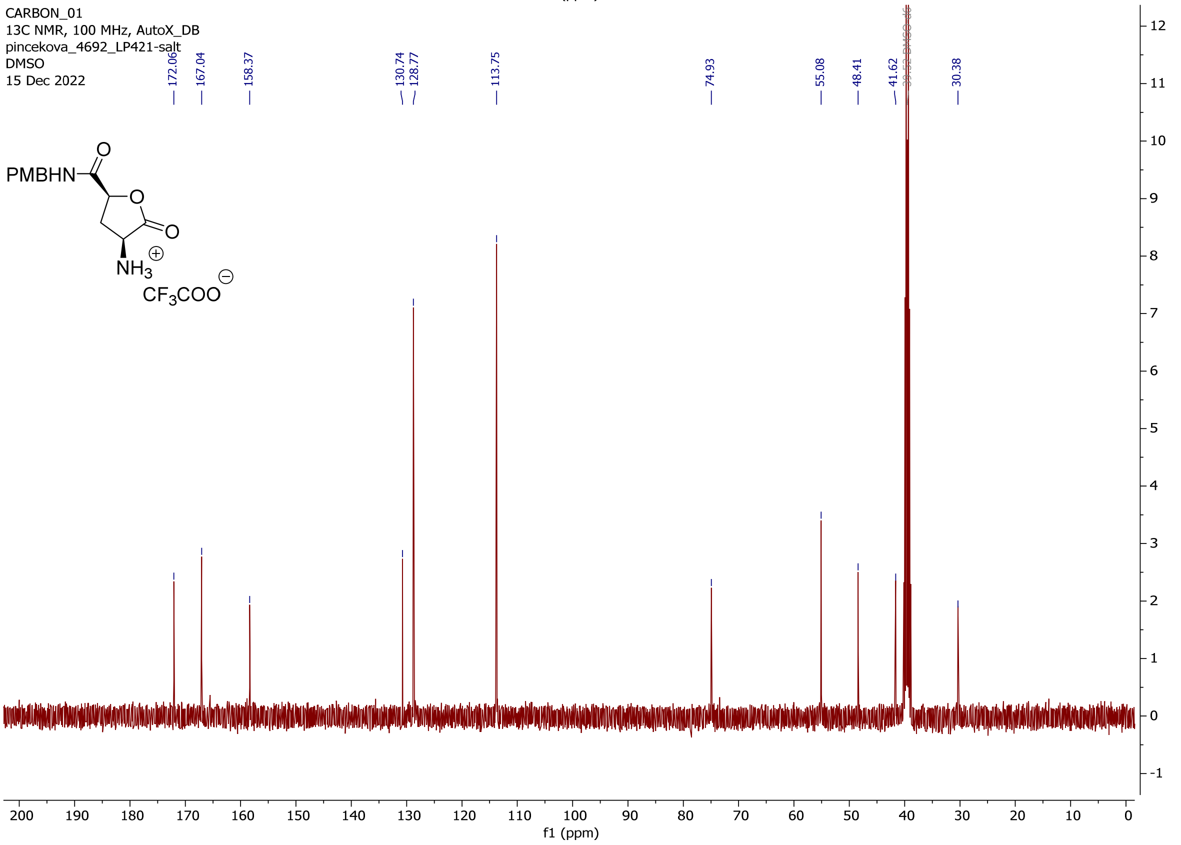

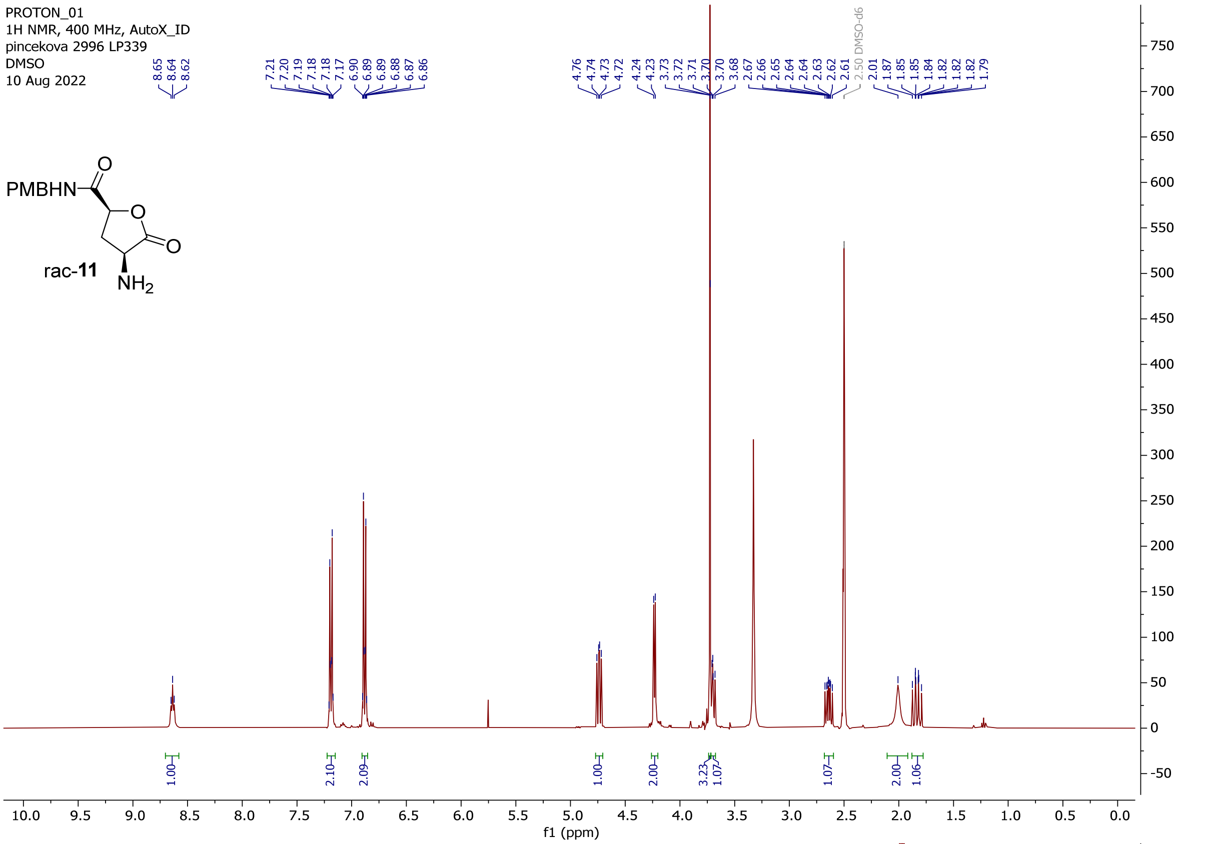
PROTON_01
1H NMR, 400 MHz, AutoX_ID
pincekova 2996 LP339
DMSO
10 Aug 2022
PMBHN
O
O
O
rac-11
NH2
8.65 8.64 8.62
7.21 7.20 7.19 7.18 7.18 7.17 6.90 6.89 6.89 6.88 6.87 6.86
4.76 4.74 4.73 4.72 4.24 4.23 3.73 3.72 3.71 3.70 3.70 3.68 2.67 2.66 2.65 2.64 2.64 2.63 2.62 2.61
2.50 DMSO-d6
2.01 1.87 1.85 1.85 1.84 1.82 1.82 1.82 1.79
1.00 2.10 2.09 1.00 2.00 3.23 1.07 1.07 2.00 1.06
f1 (ppm)

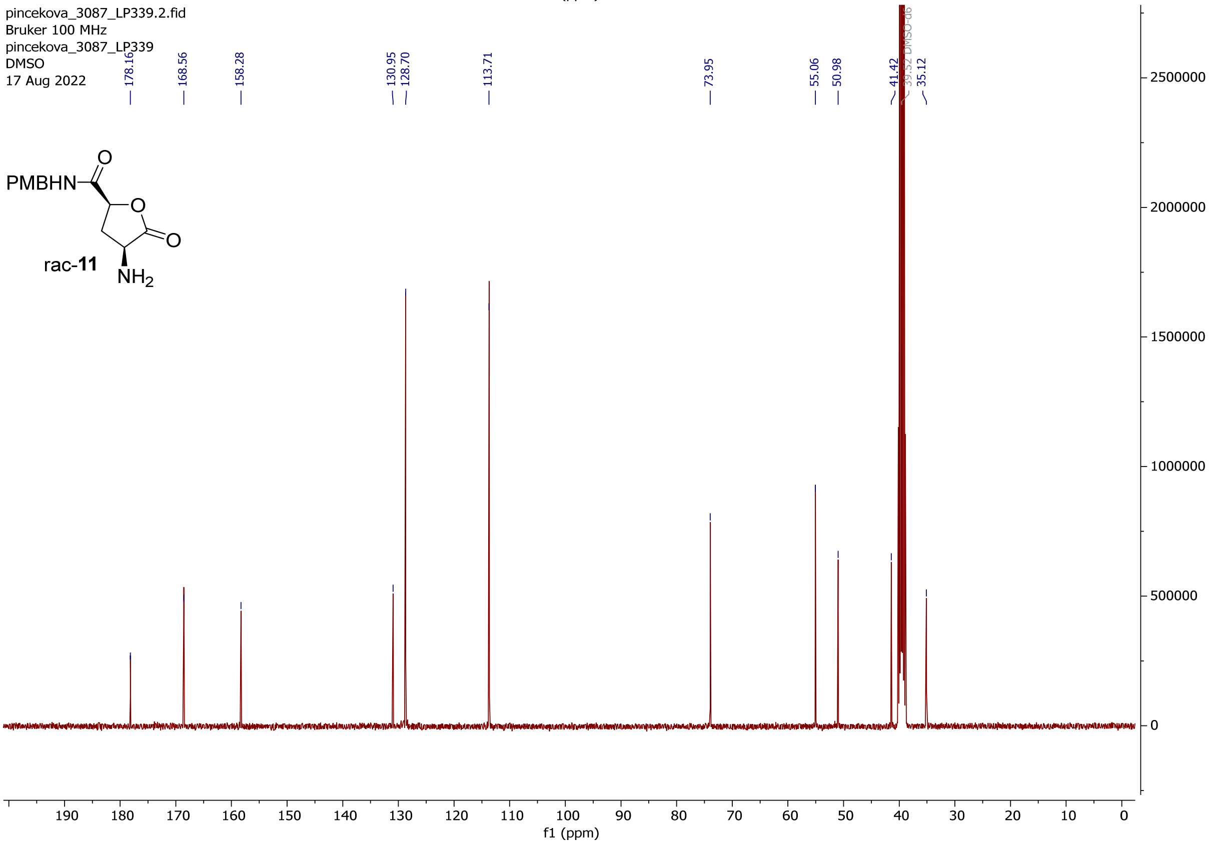
pincekova_3087_LP339.2.fid
Bruker 100 MHz
pincekova_3087_LP339
DMSO
17 Aug 2022
PMBHN
O
O
O
rac-11
NH2
178.16 168.56 158.28 130.95 128.70 113.71 73.95 55.06 50.98 41.42 39.52 DMSO-d6 35.12
f1 (ppm)

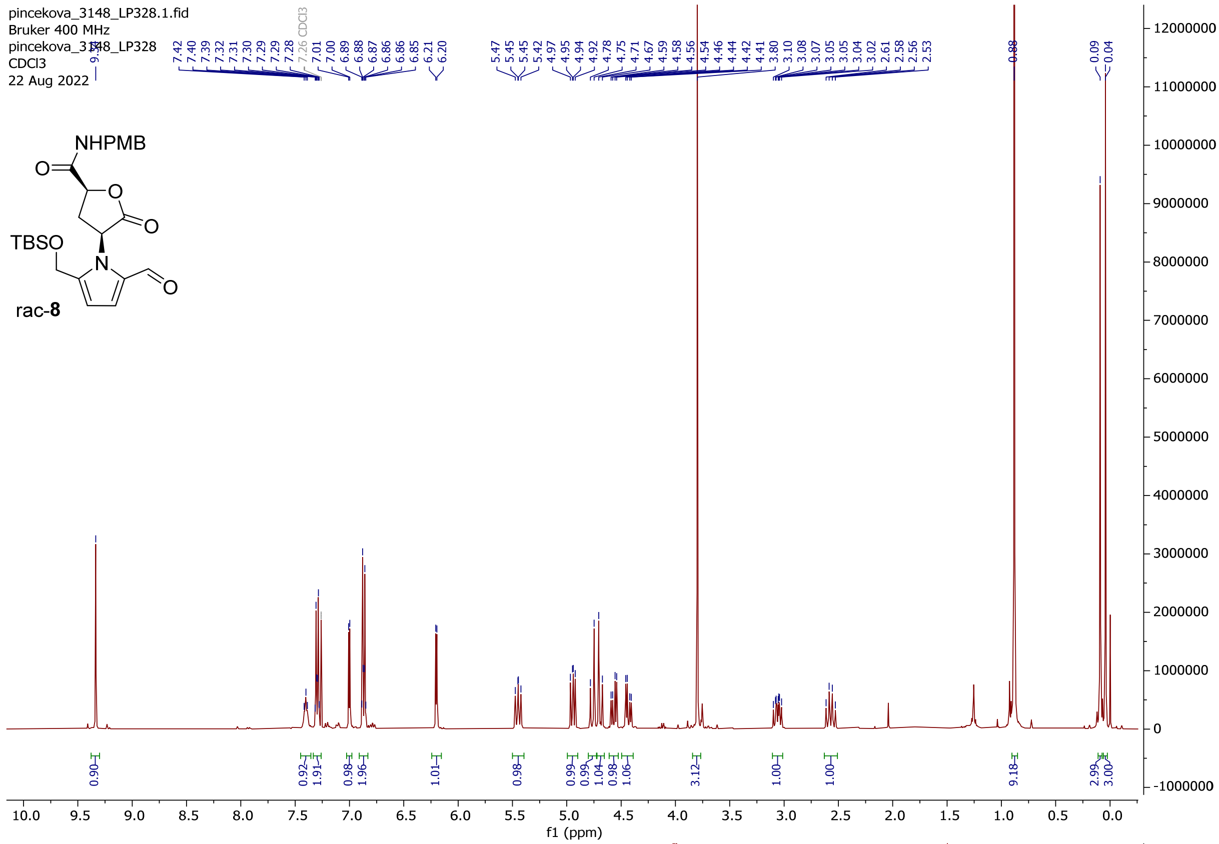
pincekova_3148_LP328.1.fid
Bruker 400 MHz
CDCl3
22 Aug 2022
7.42 7.40 7.39 7.32 7.31 7.30 7.29 7.29 7.28 7.26 CDCl3 7.01 7.00 6.89 6.88 6.87 6.86 6.86 6.85 6.21 6.20
5.47 5.45 5.45 5.42 4.97 4.95 4.94 4.92 4.78 4.75 4.71 4.67 4.59 4.58 4.56 4.54 4.46 4.44 4.42 4.41 3.80 3.10 3.08 3.07 3.05 3.05 3.04 3.02 2.61 2.58 2.56 2.53
0.88
0.09 0.04
NHPMB
O
O
O
TBSO
N
O
rac-8
0.90 0.92 1.91 0.98 1.96 1.01 0.98 0.99 0.99 1.04 0.98 1.06 3.12 1.00 1.00 9.18 2.99 3.00
f1 (ppm)

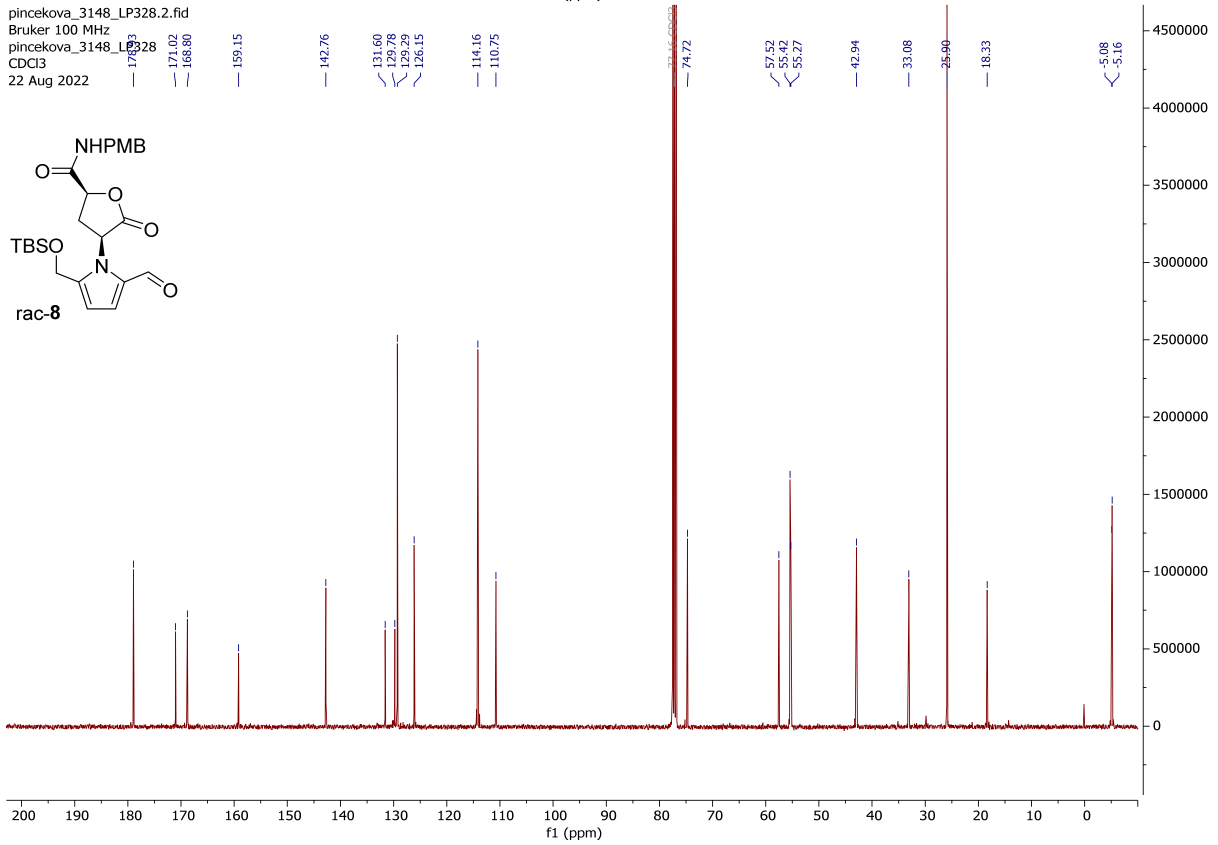
pincekova_3148_LP328.2.fid
Bruker 100 MHz
CDCl3
22 Aug 2022
171.02 168.80 159.15 142.76 131.60 129.78 129.29 126.15 114.16 110.75 74.72 57.52 55.42 55.27 42.94 33.08 25.90 18.33 -5.08 -5.16
NHPMB
O
O
O
TBSO
N
O
rac-8
f1 (ppm)

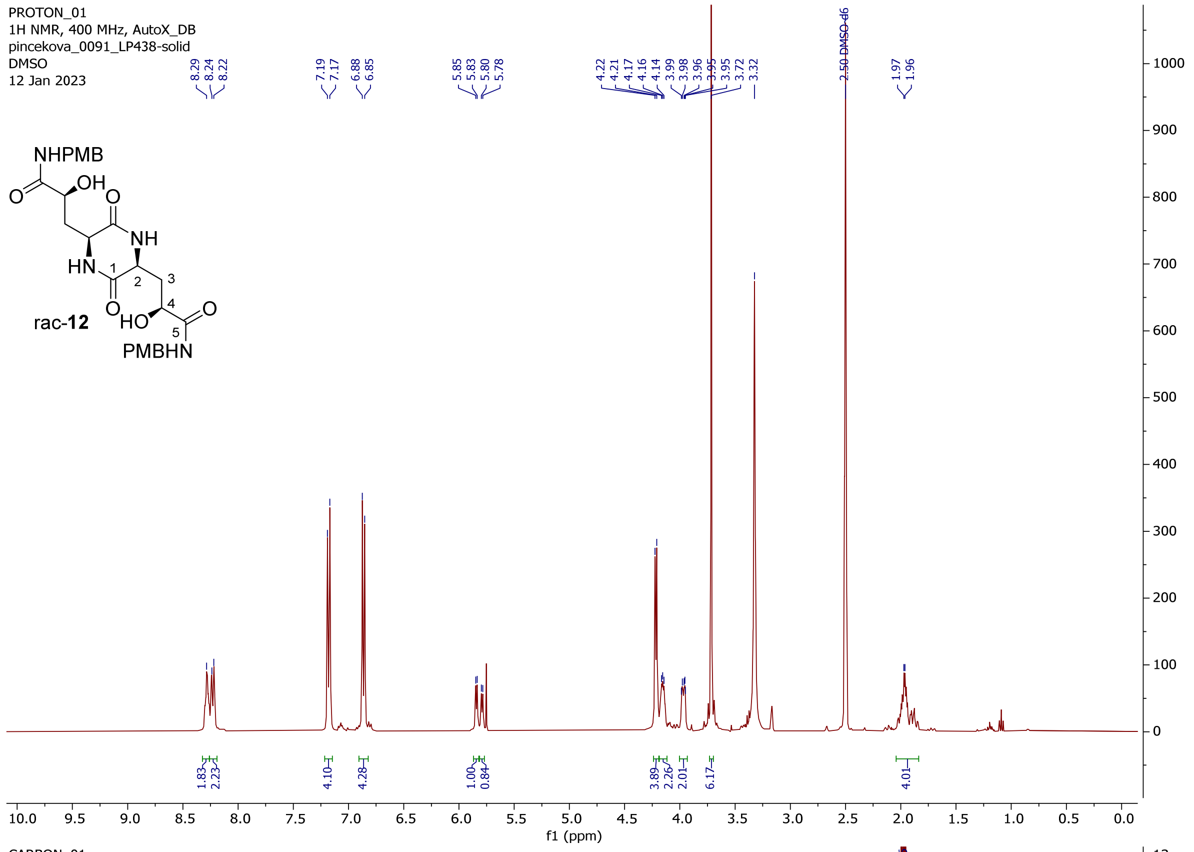
PROTON_01
1H NMR, 400 MHz, AutoX_DB
pincekova_0091_LP438-solid
DMSO
12 Jan 2023
NHPMB
OH
O
NH
HN
1
2
3
4
5
HO
PMBHN
rac-12
8.29
8.24
8.22
7.19
7.17
6.88
6.85
5.85
5.83
5.80
5.78
4.22
4.21
4.17
4.16
4.14
3.99
3.98
3.96
3.95
3.95
3.72
3.32
2.50 DMSO-d6
1.97
1.96
1.83
2.23
4.10
4.28
1.00
0.84
3.89
2.26
2.01
6.17
4.01
f1 (ppm)

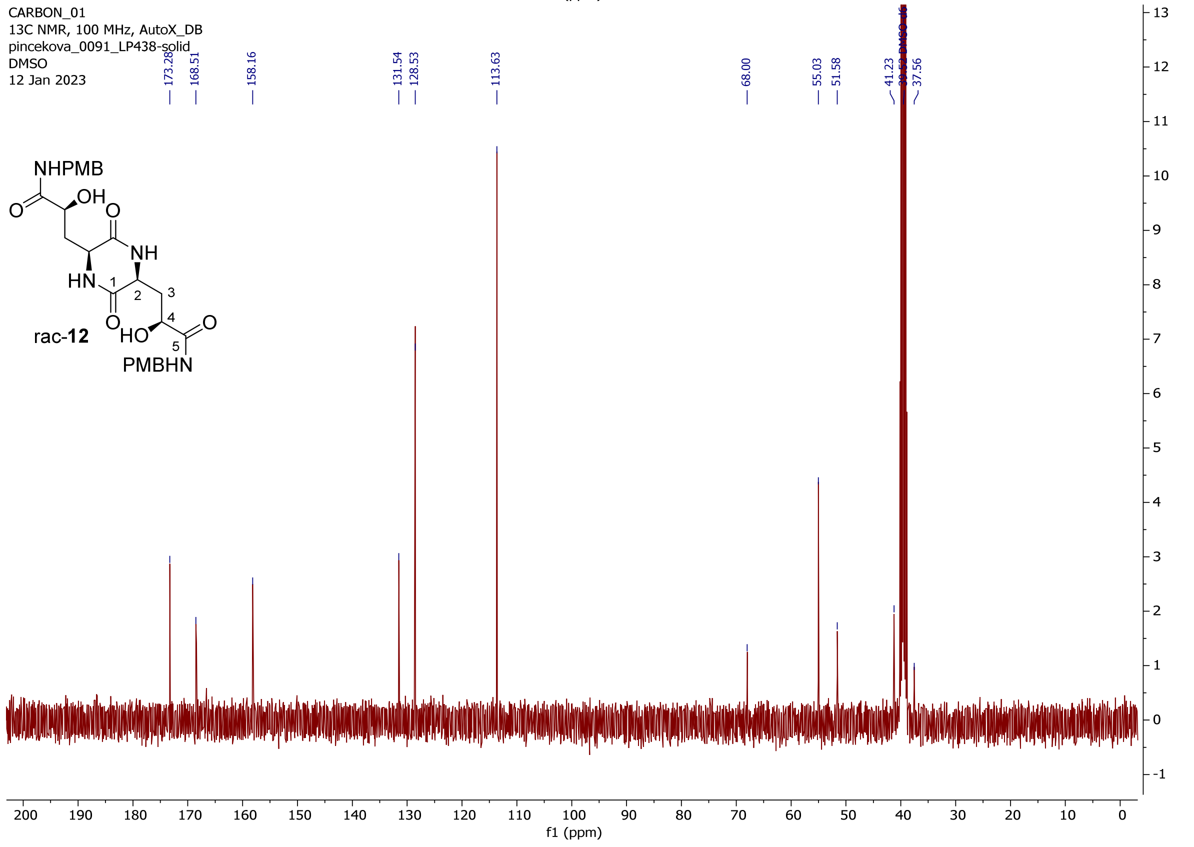
CARBON_01
13C NMR, 100 MHz, AutoX_DB
pincekova_0091_LP438-solid
DMSO
12 Jan 2023
173.28
168.51
158.16
131.54
128.53
113.63
68.00
55.03
51.58
41.23
37.56
NHPMB
OH
O
NH
HN
1
2
3
4
5
HO
PMBHN
rac-12
f1 (ppm)

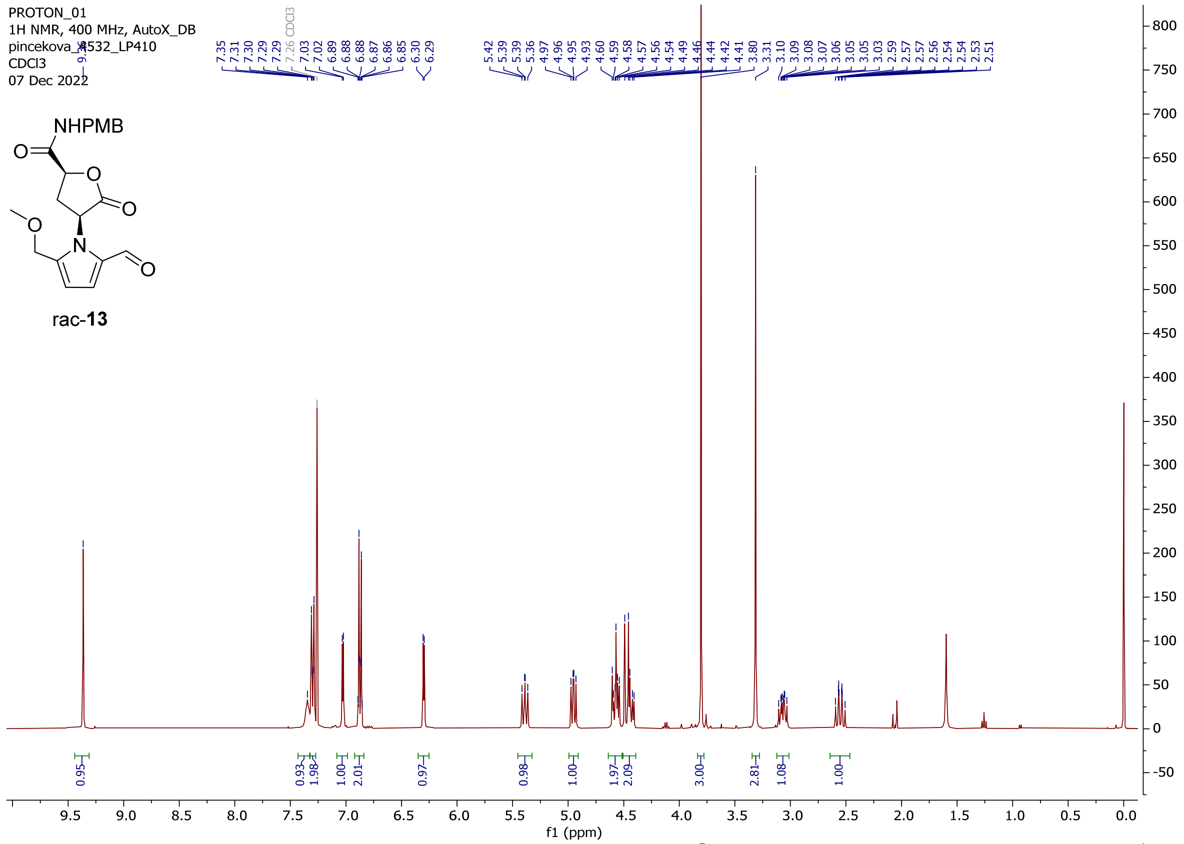
PROTON_01
1H NMR, 400 MHz, AutoX_DB
pincekova_4532_LP410
CDCl3
07 Dec 2022
NHPMB
rac-13
7.35 7.31 7.30 7.29 7.29 7.26 CDCl3 7.03 7.02 6.89 6.88 6.88 6.87 6.86 6.85 6.30 6.29
5.42 5.39 5.39 5.36 4.97 4.96 4.95 4.93 4.60 4.59 4.58 4.57 4.56 4.54 4.49 4.46 4.44 4.42 4.41 3.80 3.31 3.10 3.09 3.08 3.07 3.06 3.05 3.05 3.03 2.59 2.57 2.57 2.56 2.54 2.54 2.53 2.51
0.95 0.93 1.98 1.00 2.01 0.97 0.98 1.00 1.97 2.09 3.00 2.81 1.08 1.00
f1 (ppm)

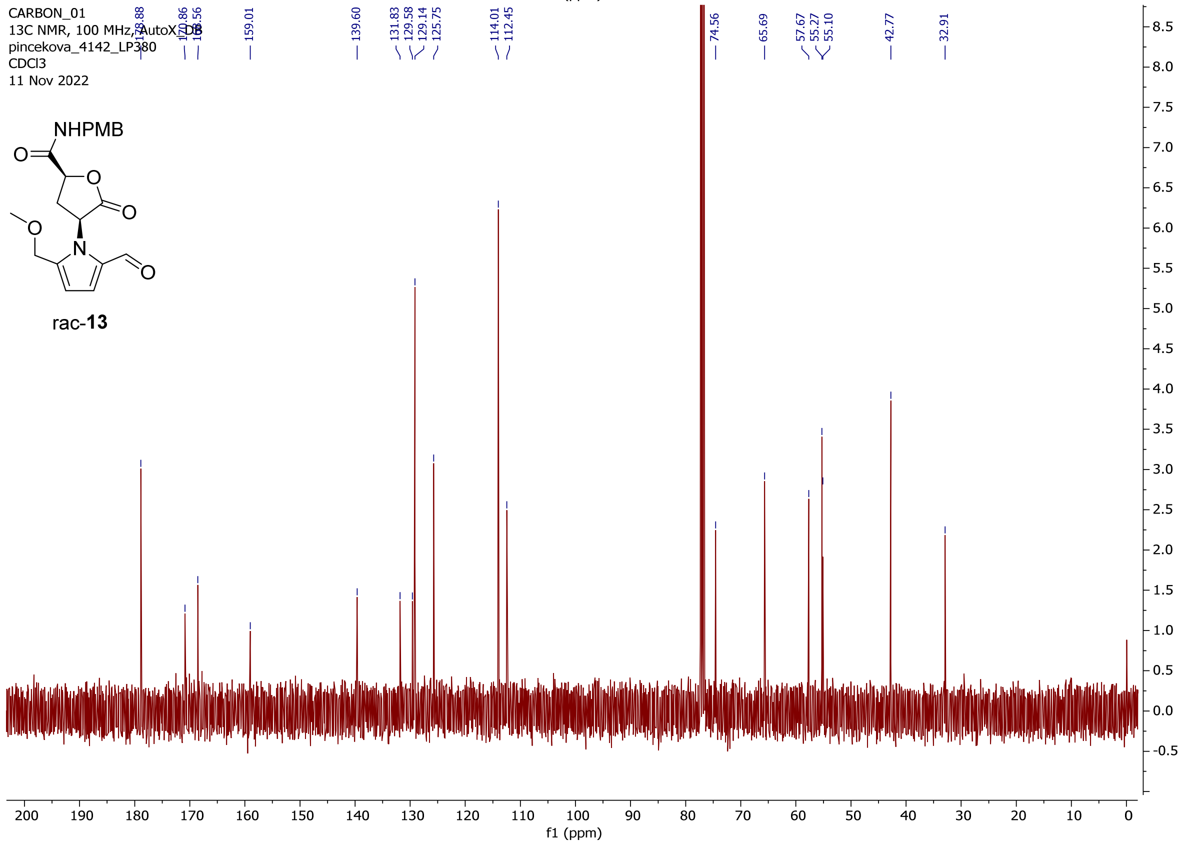
CARBON_01
13C NMR, 100 MHz, AutoX_DB
pincekova_4142_LP380
CDCl3
11 Nov 2022
NHPMB
rac-13
178.88 170.86 168.56 159.01 139.60 131.83 129.58 129.14 125.75 114.01 112.45 74.56 65.69 57.67 55.27 55.10 42.77 32.91
f1 (ppm)

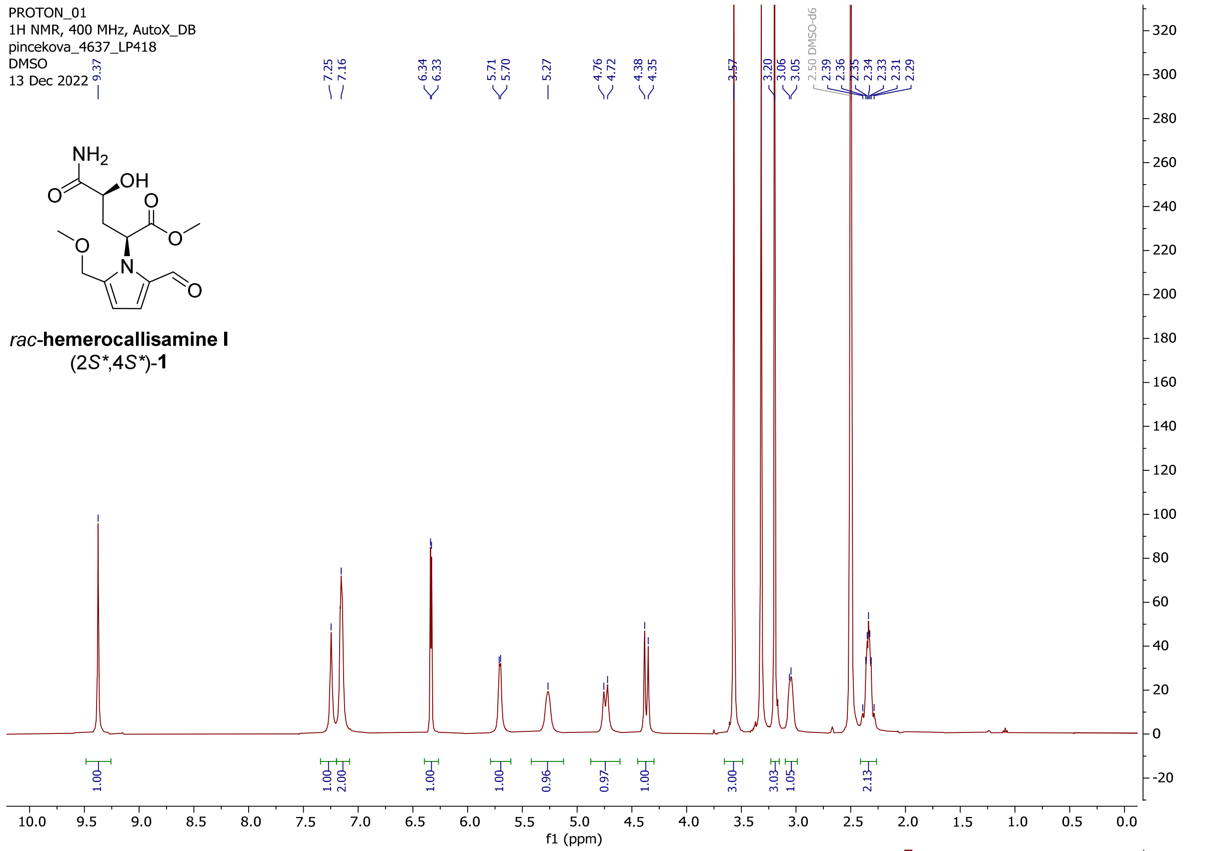
PROTON_01
1H NMR, 400 MHz, AutoX_DB
pincekova_4637_LP418
DMSO
13 Dec 2022
9.37
7.25
7.16
6.34
6.33
5.71
5.70
5.27
4.76
4.72
4.38
4.35
3.57
3.20
3.06
3.05
2.50 DMSO-d6
2.39
2.36
2.35
2.34
2.33
2.31
2.29
1.00
1.00
2.00
1.00
1.00
0.96
0.97
1.00
3.00
3.03
1.05
2.13
NH2
OH
O
N
rac-hemerocallisamine I
(2S*,4S*)-1
f1 (ppm)

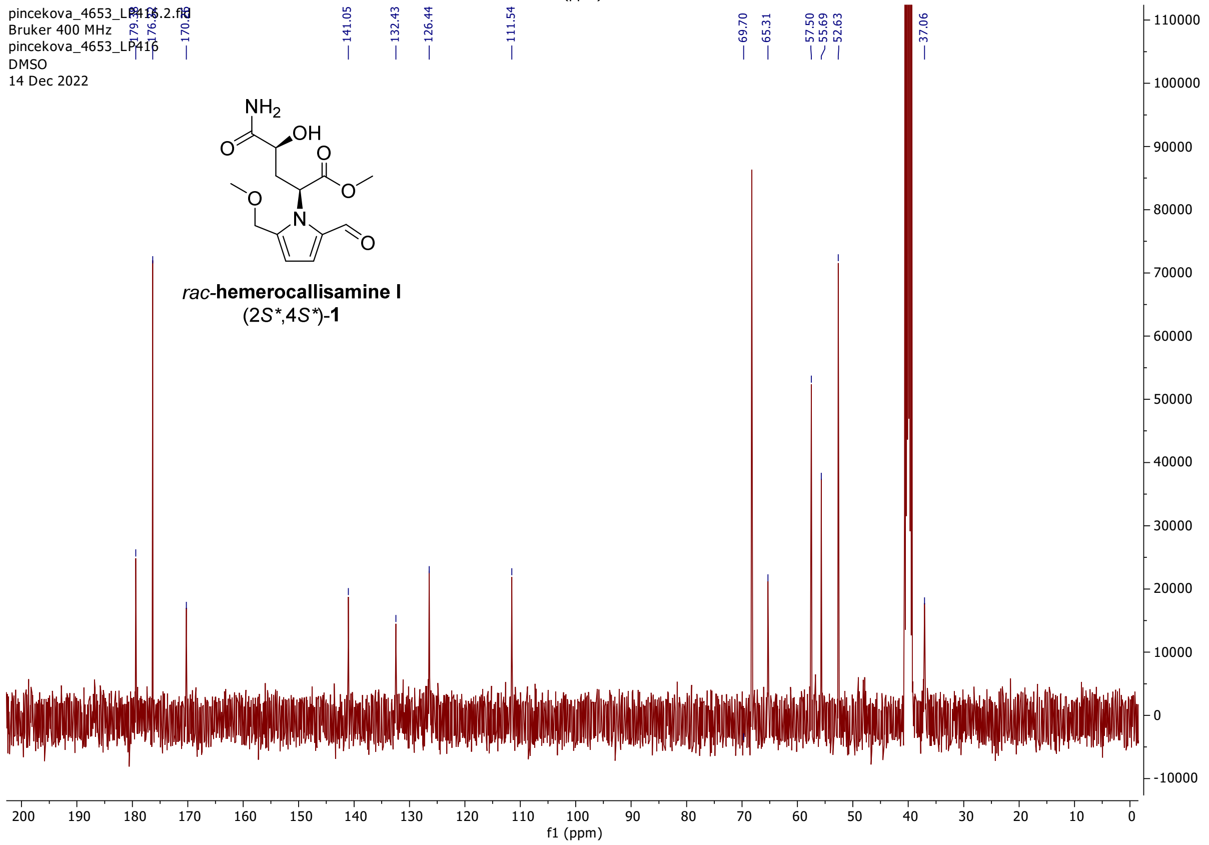
Bruker 400 MHz
pincekova_4653_LP416
DMSO
14 Dec 2022
141.05
132.43
126.44
111.54
69.70
65.31
57.50
55.69
52.63
37.06
NH2
OH
O
N
rac-hemerocallisamine I
(2S*,4S*)-1
f1 (ppm)

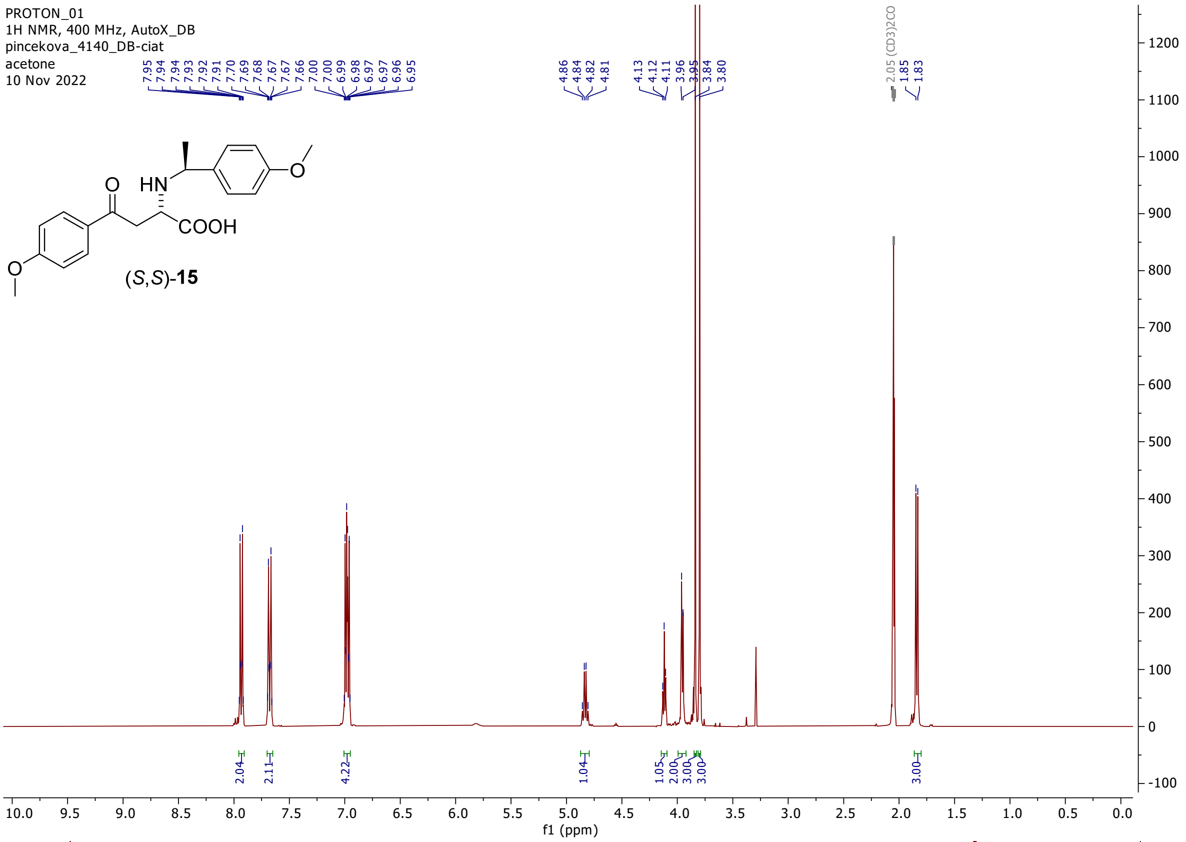
PROTON_01
1H NMR, 400 MHz, AutoX_DB
pincekova_4140_DB-ciat
acetone
10 Nov 2022
7.95 7.94 7.94 7.93 7.92 7.91 7.70 7.69 7.68 7.67 7.67 7.66 7.00 7.00 6.99 6.98 6.97 6.97 6.96 6.95
4.86 4.84 4.82 4.81
4.13 4.12 4.11 3.96 3.95 3.84 3.80
2.05 (CD3)2CO
1.85 1.83
O
HN
COOH
O
(S,S)-15
2.04 2.11 4.22 1.04 1.05 2.00 3.00 3.00 3.00
f1 (ppm)

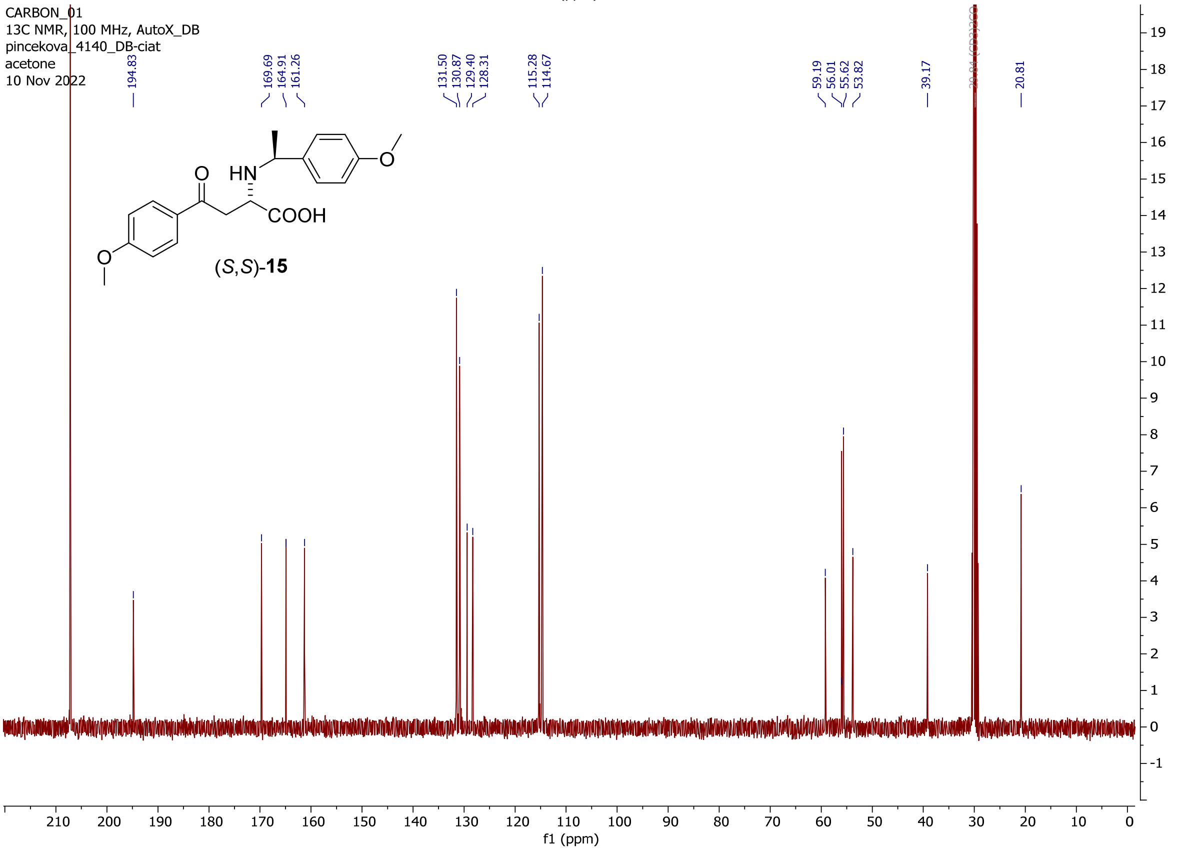
CARBON_01
13C NMR, 100 MHz, AutoX_DB
pincekova_4140_DB-ciat
acetone
10 Nov 2022
194.83
169.69 164.91 161.26
131.50 130.87 129.40 128.31
115.28 114.67
59.19 56.01 55.62 53.82
39.17
20.81
O
HN
COOH
O
(S,S)-15
f1 (ppm)

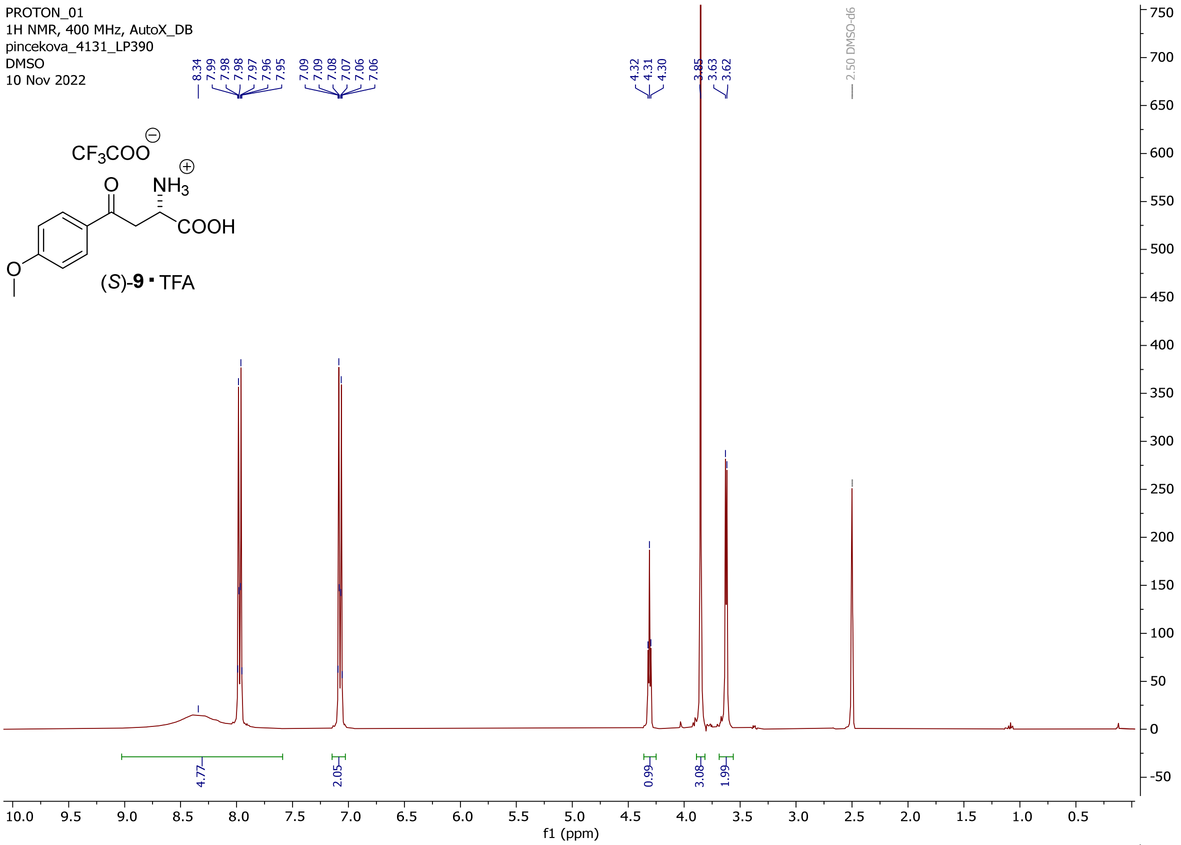
PROTON_01
1H NMR, 400 MHz, AutoX_DB
pincekova_4131_LP390
DMSO
10 Nov 2022
8.34
7.99
7.98
7.98
7.97
7.96
7.95
7.09
7.09
7.08
7.07
7.06
7.06
4.32
4.31
4.30
3.85
3.63
3.62
2.50 DMSO-d6
$CF_3COO^{\ominus}$
O
$NH_3^{\oplus}$
COOH
O
(*S*)-**9** · TFA
4.77
2.05
0.99
3.08
1.99
f1 (ppm)

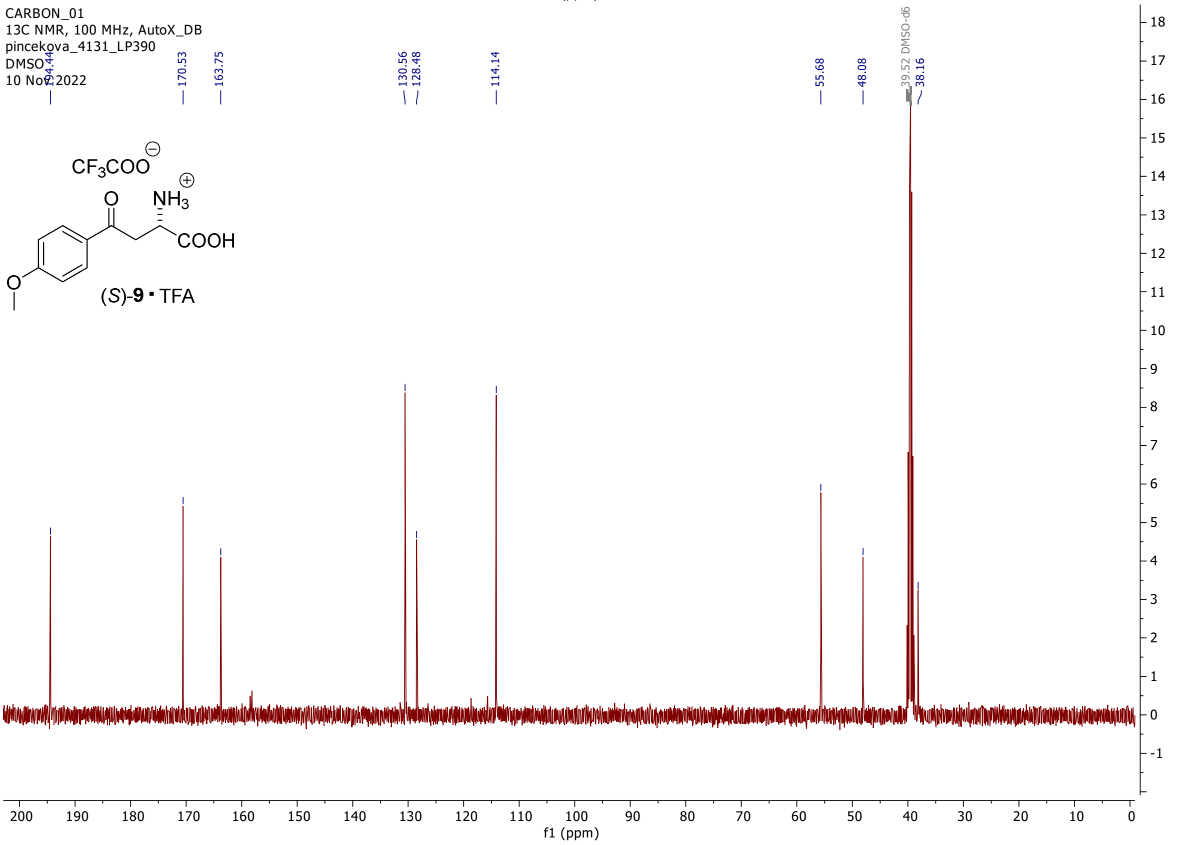
CARBON_01
13C NMR, 100 MHz, AutoX_DB
pincekova_4131_LP390
DMSO
10 Nov 2022
194.44
170.53
163.75
130.56
128.48
114.14
55.68
48.08
39.52 DMSO-d6
38.16
$CF_3COO^{\ominus}$
O
$NH_3^{\oplus}$
COOH
O
(*S*)-**9** · TFA
f1 (ppm)

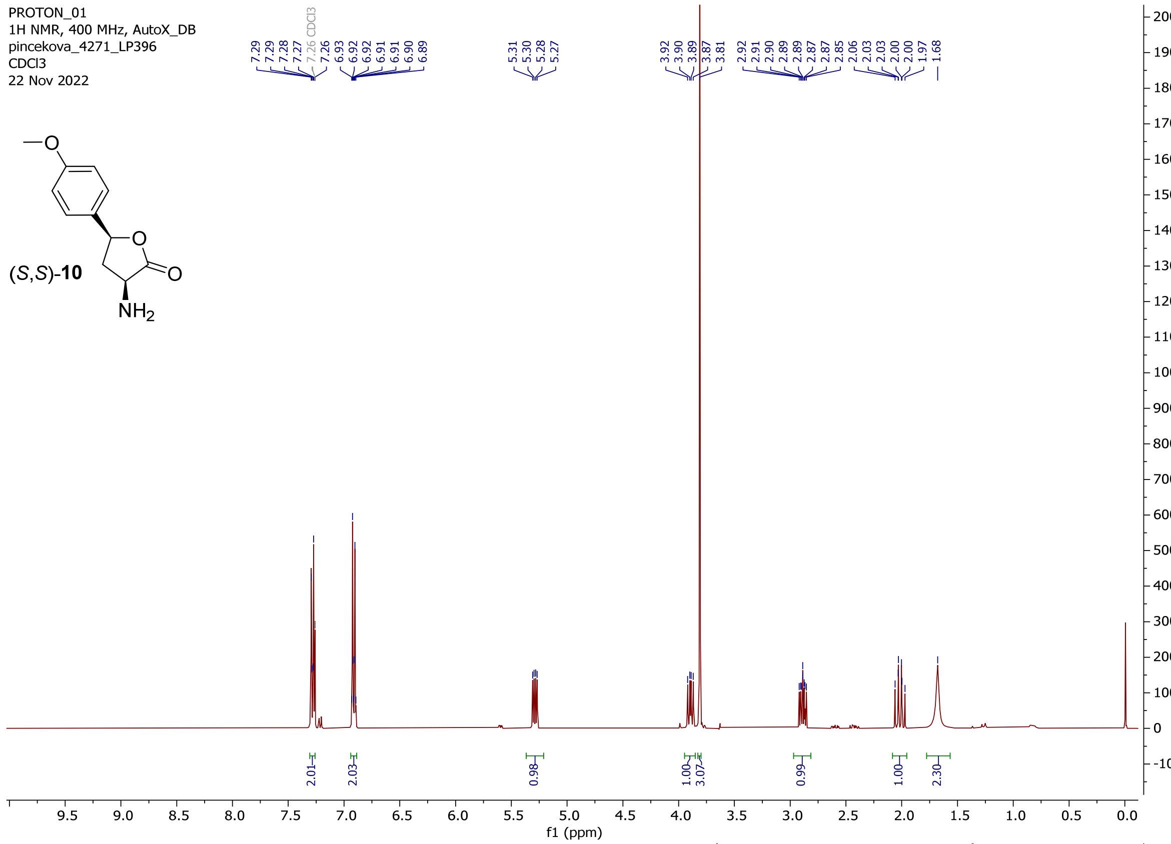
PROTON_01
1H NMR, 400 MHz, AutoX_DB
pincekova_4271_LP396
CDCl3
22 Nov 2022
(S,S)-10
NH2
f1 (ppm)

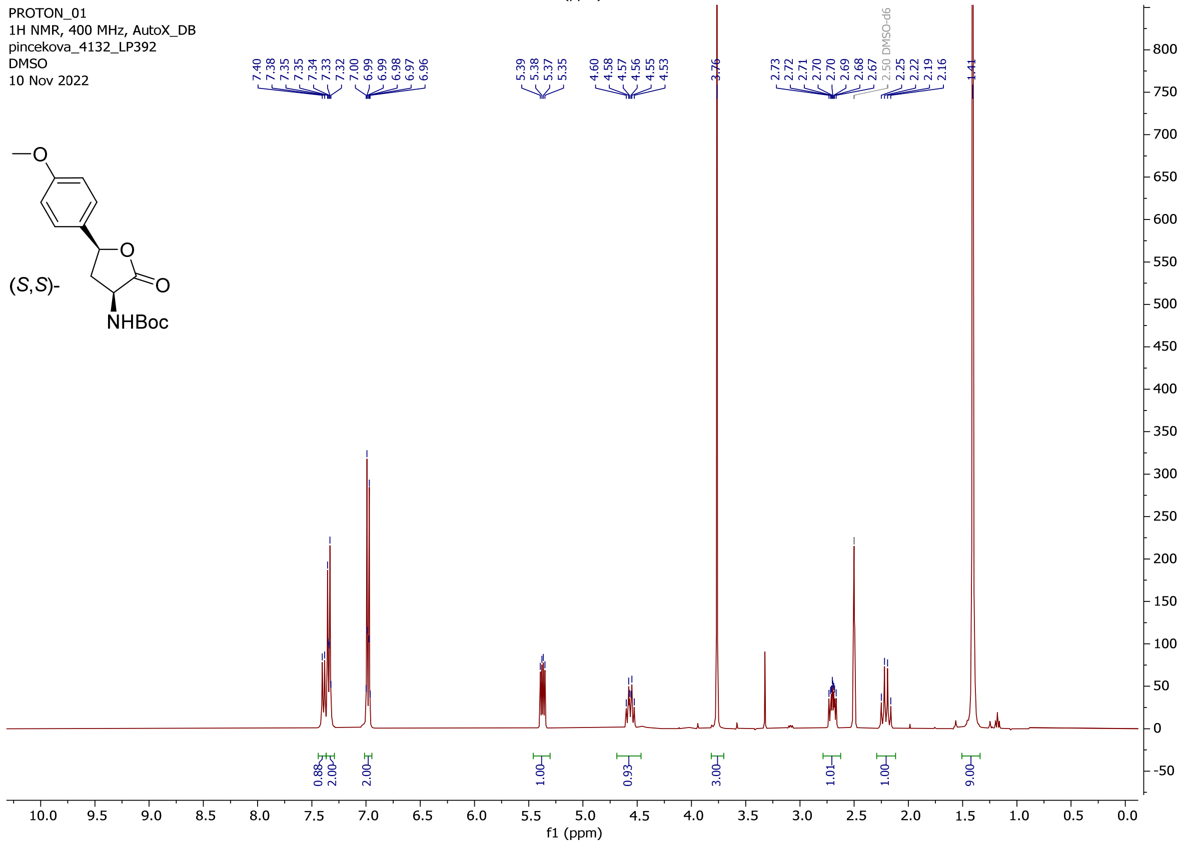
PROTON_01
1H NMR, 400 MHz, AutoX_DB
pincekova_4132_LP392
DMSO
10 Nov 2022
(S,S)-
NHBoc
f1 (ppm)

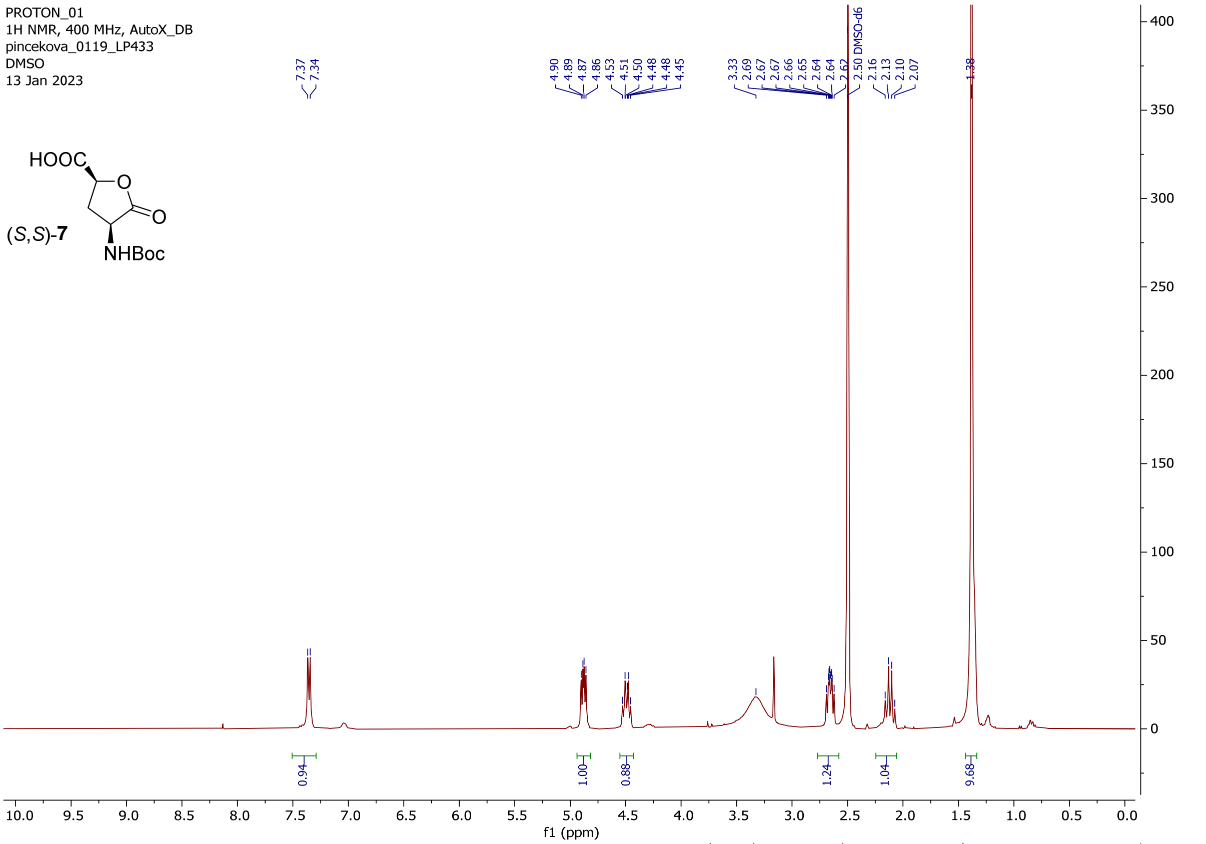
PROTON_01
1H NMR, 400 MHz, AutoX_DB
pincekova_0119_LP433
DMSO
13 Jan 2023
7.37
7.34
4.90
4.89
4.87
4.86
4.53
4.51
4.50
4.48
4.48
4.45
3.33
2.69
2.67
2.67
2.66
2.65
2.64
2.64
2.62
2.50 DMSO-d6
2.16
2.13
2.10
2.07
1.38
HOOC
O
O
(S,S)-7
NHBoc
0.94
1.00
0.88
1.24
1.04
9.68
f1 (ppm)

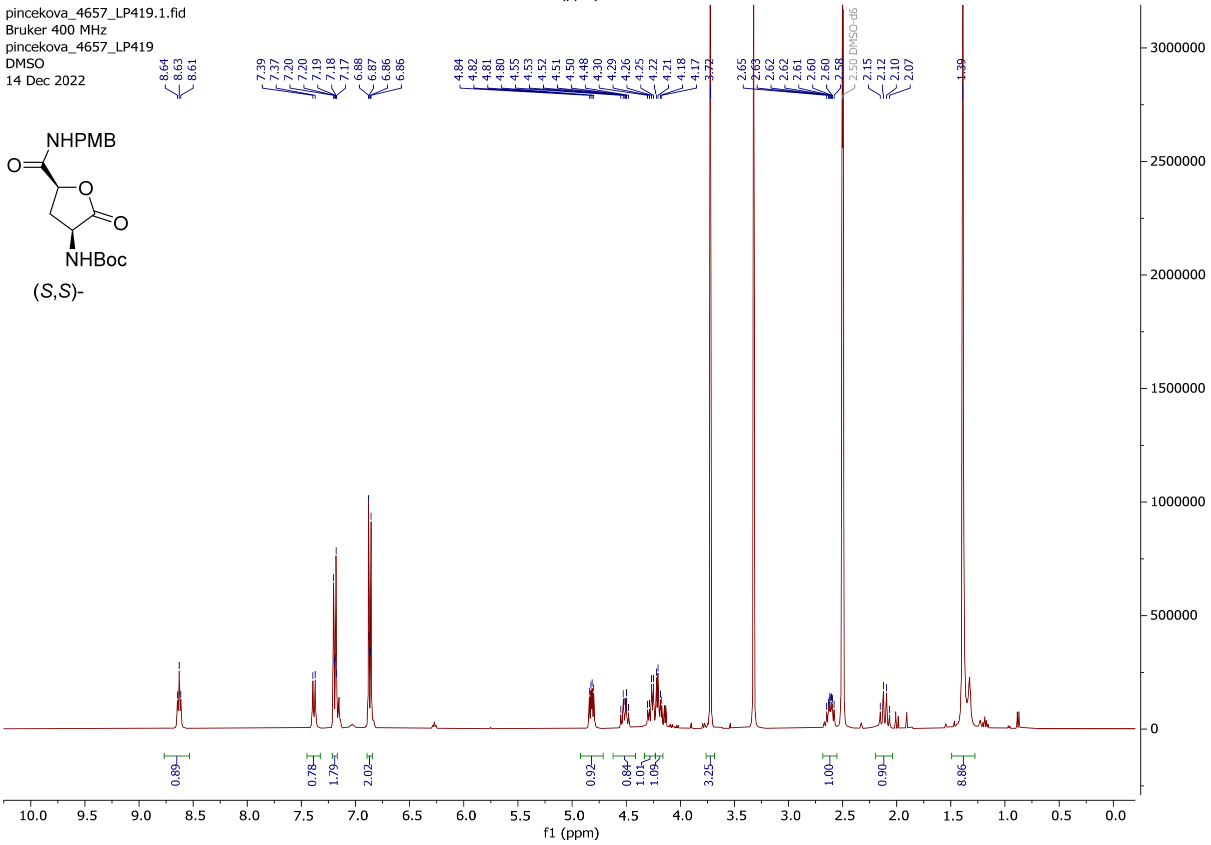
pincekova_4657_LP419.1.fid
Bruker 400 MHz
pincekova_4657_LP419
DMSO
14 Dec 2022
8.64
8.63
8.61
7.39
7.37
7.20
7.20
7.19
7.18
7.17
6.88
6.87
6.86
6.86
4.84
4.82
4.81
4.80
4.55
4.53
4.52
4.51
4.50
4.48
4.30
4.29
4.26
4.25
4.22
4.21
4.18
4.17
3.72
2.65
2.63
2.62
2.61
2.60
2.60
2.58
2.50 DMSO-d6
2.15
2.12
2.10
2.07
1.39
NHPMB
O
O
O
NHBoc
(S,S)-
0.89
0.78
1.79
2.02
0.92
0.84
1.01
1.09
3.25
1.00
0.90
8.86
f1 (ppm)

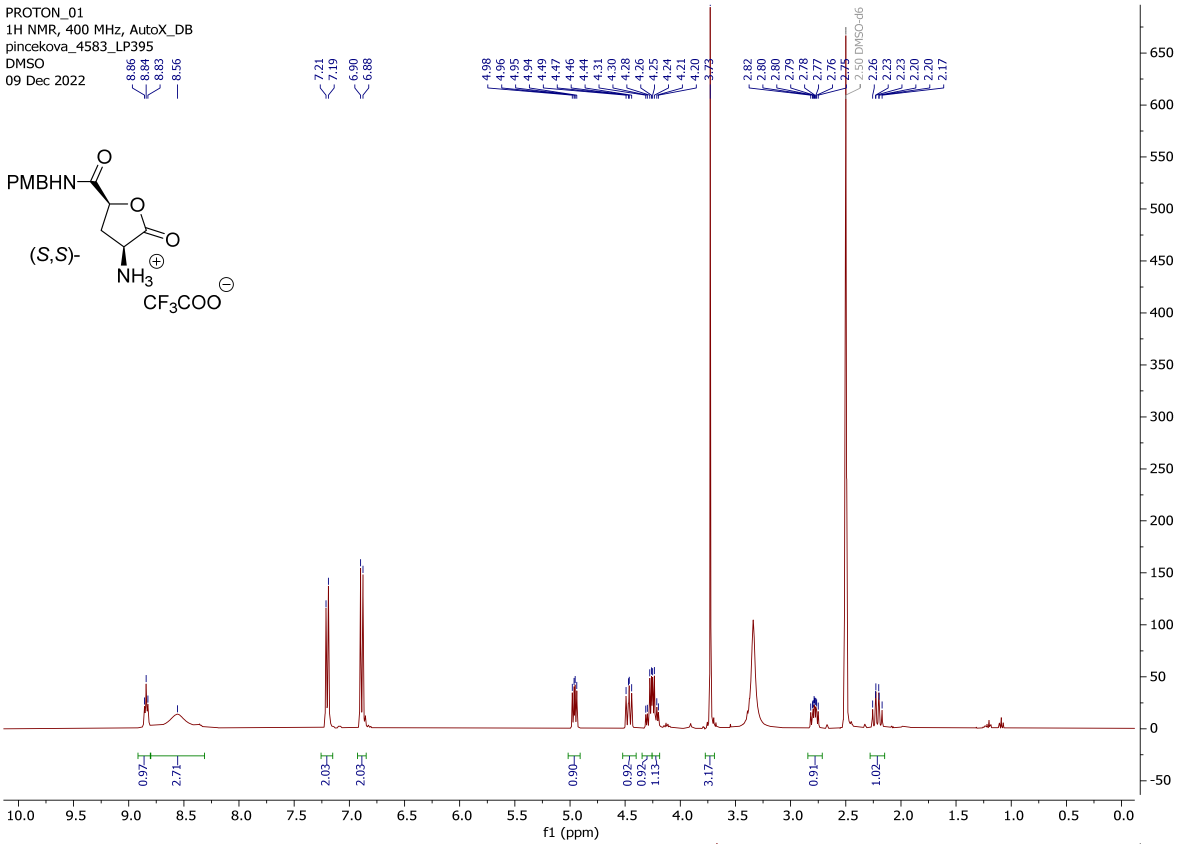
PROTON_01
1H NMR, 400 MHz, AutoX_DB
pincekova_4583_LP395
DMSO
09 Dec 2022
8.86
8.84
8.83
8.56
7.21
7.19
6.90
6.88
4.98
4.96
4.95
4.94
4.49
4.47
4.46
4.44
4.31
4.30
4.28
4.26
4.25
4.24
4.21
4.20
3.73
2.82
2.80
2.80
2.79
2.78
2.77
2.76
2.75
2.50 DMSO-d6
2.26
2.23
2.23
2.20
2.20
2.17
PMBHN
O
O
O
(S,S)-
NH3
CF3COO
0.97
2.71
2.03
2.03
0.90
0.92
0.92
1.13
3.17
0.91
1.02
f1 (ppm)

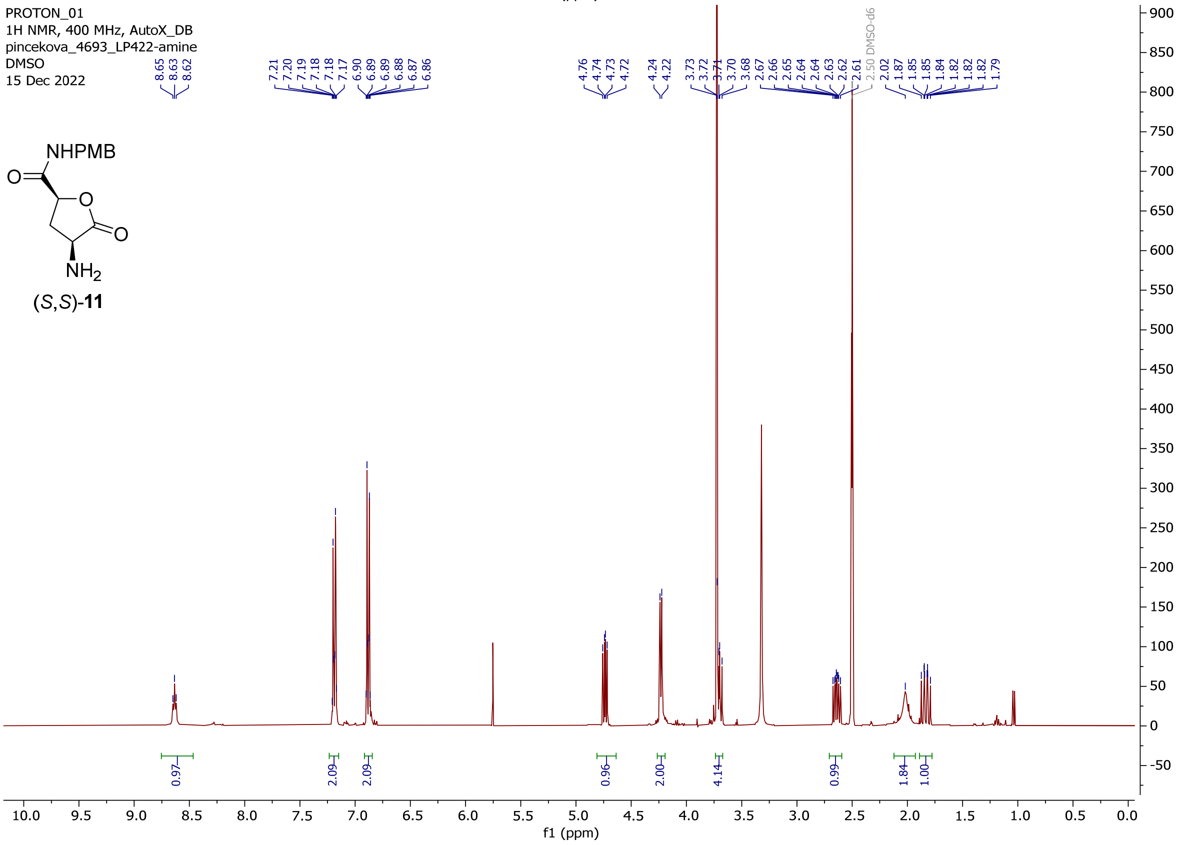
PROTON_01
1H NMR, 400 MHz, AutoX_DB
pincekova_4693_LP422-amine
DMSO
15 Dec 2022
8.65
8.63
8.62
7.21
7.20
7.19
7.18
7.18
7.17
6.90
6.89
6.89
6.88
6.87
6.86
4.76
4.74
4.73
4.72
4.24
4.22
3.73
3.72
3.71
3.70
3.68
2.67
2.66
2.65
2.64
2.64
2.63
2.62
2.61
2.50 DMSO-d6
2.02
1.87
1.85
1.85
1.84
1.82
1.82
1.82
1.79
NHPMB
O
O
O
NH2
(S,S)-11
0.97
2.09
2.09
0.96
2.00
4.14
0.99
1.84
1.00
f1 (ppm)

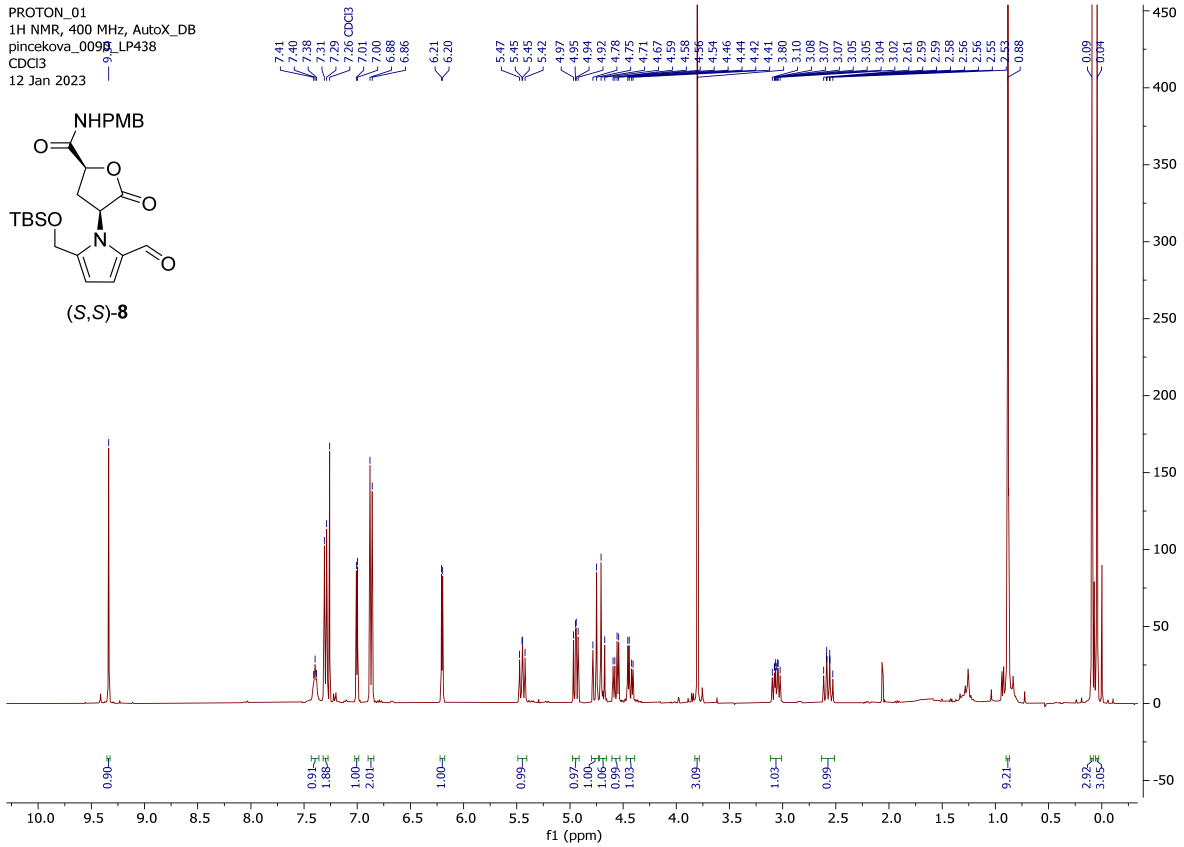
PROTON_01
1H NMR, 400 MHz, AutoX_DB
pincekova_0096_LP438
CDCl3
12 Jan 2023
NHPMB
TBSO
(S,S)-8
f1 (ppm)

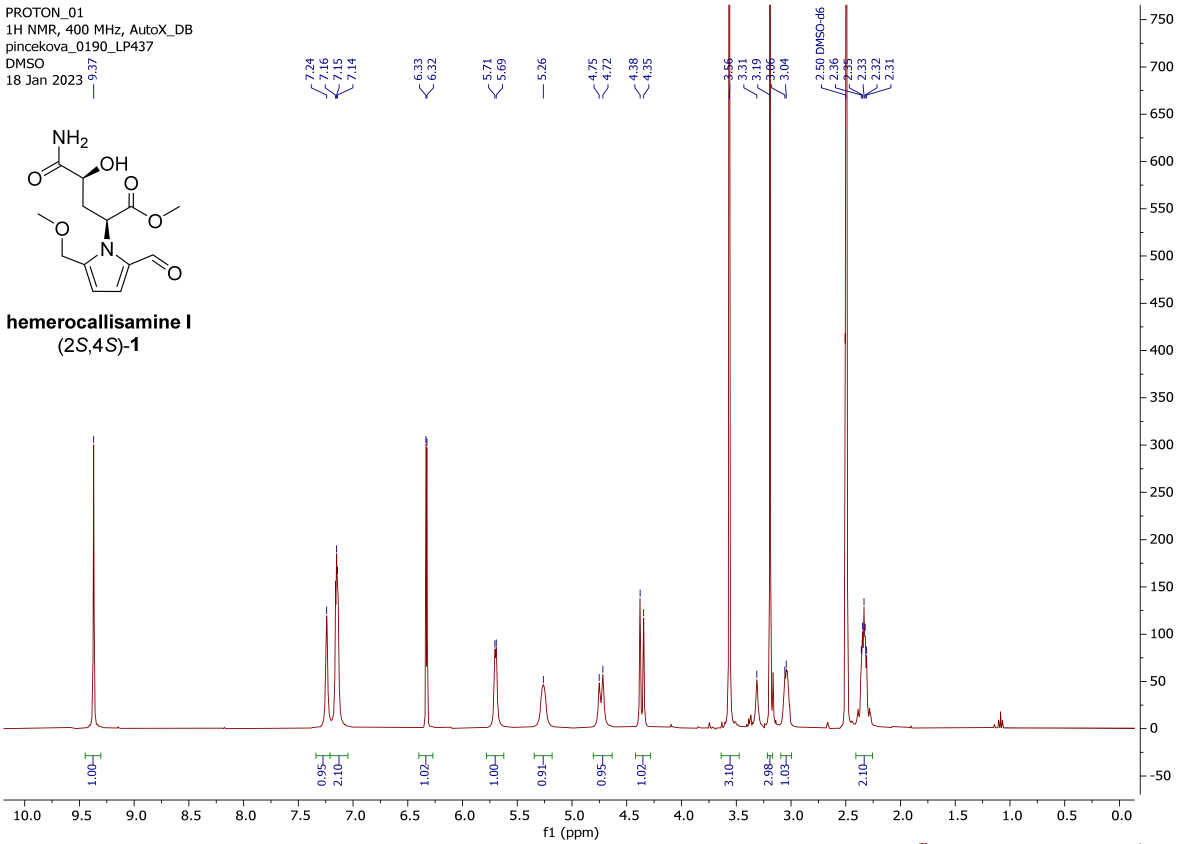
PROTON_01
1H NMR, 400 MHz, AutoX_DB
pincekova_0190_LP437
DMSO
18 Jan 2023
NH2
OH
O
O
O
O
N
O
hemerocallisamine I
(2S,4S)-1
9.37
7.24
7.16
7.15
7.14
6.33
6.32
5.71
5.69
5.26
4.75
4.72
4.38
4.35
3.56
3.31
3.19
3.06
3.04
2.50 DMSO-d6
2.36
2.35
2.33
2.32
2.31
1.00
0.95
2.10
1.02
1.00
0.91
0.95
1.02
3.10
2.98
1.03
2.10
f1 (ppm)

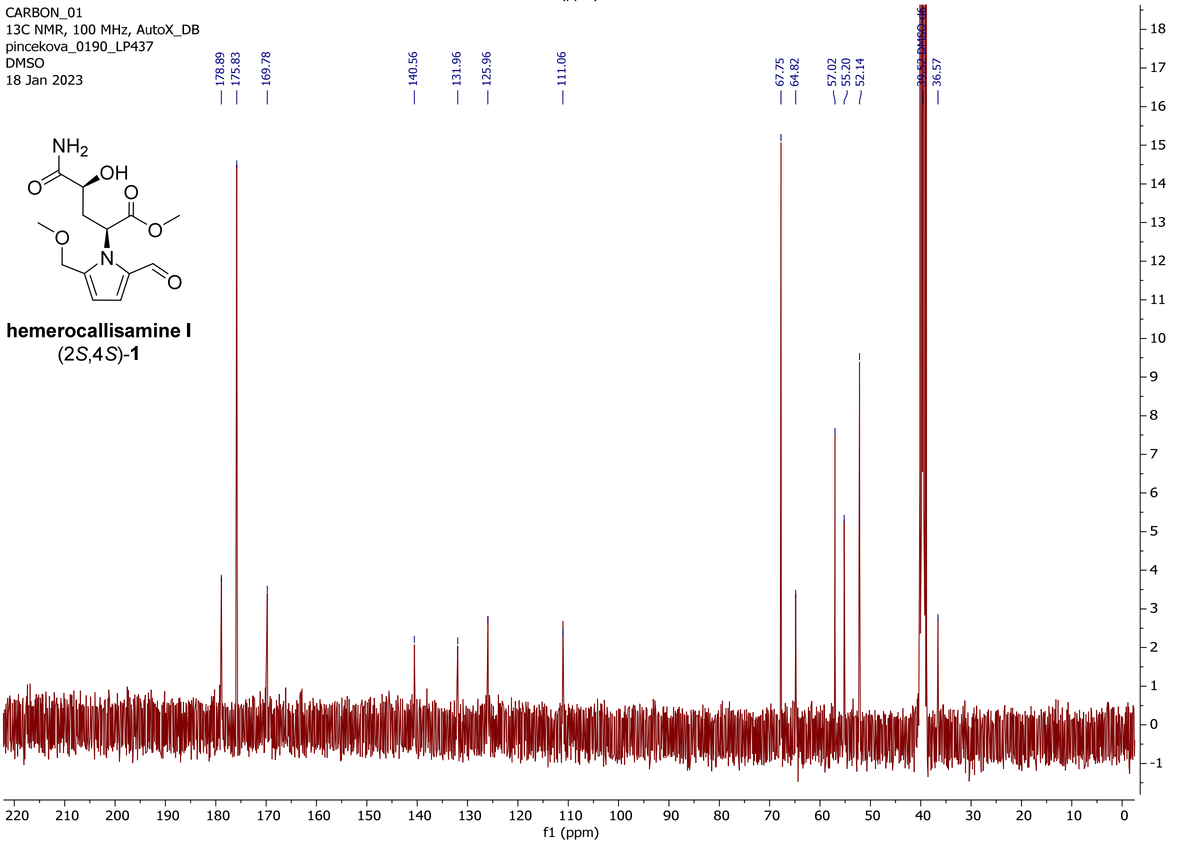
CARBON_01
13C NMR, 100 MHz, AutoX_DB
pincekova_0190_LP437
DMSO
18 Jan 2023
NH2
OH
O
O
O
O
N
O
hemerocallisamine I
(2S,4S)-1
178.89
175.83
169.78
140.56
131.96
125.96
111.06
67.75
64.82
57.02
55.20
52.14
39.52 DMSO-d6
36.57
f1 (ppm)

1. Sarkar, S. K.; Upul Ranaweera, R. A.; Merugu, R.; Abdelaziz, N. M.; Robinson, J.; Day, H. A.; Krause, J. A.; Gudmundsdottir, A. D., Comparison of the Photochemistry of Acyclic and Cyclic 4-(4-Methoxy-phenyl)-4-oxo-but-2-enoate Ester Derivatives. *The Journal of Physical Chemistry A* **2020,** *124* (37), 7346-7354.
2. Subbiah, S.; Simeonov, S. P.; Esperança, J. M.; Rebelo, L. P. N.; Afonso, C. A., Direct transformation of 5-hydroxymethylfurfural to the building blocks 2, 5-dihydroxymethylfurfural (DHMF) and 5-hydroxymethyl furanoic acid (HMFA) via Cannizzaro reaction. *Green chemistry* **2013,** *15* (10), 2849-2853.
3. Geng, H. M.; Stubbing, L. A.; Li-yang Chen, J.; Furkert, D. P.; Brimble, M. A., Synthesis of the revised structure of acortatarin A. *European Journal of Organic Chemistry* **2014,** *2014* (28), 6227-6241.
4. Sheldrick, G. M., SHELXT–Integrated space-group and crystal-structure determination. *Acta Crystallographica Section A: Foundations and Advances* **2015,** *71* (1), 3-8.
5. Sheldrick, G. M., Crystal structure refinement with SHELXL. *Acta Crystallographica Section C: Structural Chemistry* **2015,** *71* (1), 3-8.
